# Supplementary material for: Optimal low-depth quantum signal-processing phase estimation
Source: Nat Commun. 2025 Feb 10;16:1504. doi: 10.1038/s41467-025-56724-x (PMC11811185; doi:10.1038/s41467-025-56724-x)
Supplement: Supplementary file 1 — Supplementary Information [file 41467_2025_56724_MOESM1_ESM.pdf]

Supplementary Information for Optimal Low-Depth Quantum Signal-Processing Phase Estimation

Yulong Dong,[1,2,*] Jonathan A. Gross,[1] and Murphy Yuezhen Niu[1,3,†]

[1] *Google Quantum AI, Venice, California 90291, USA.*
[2] *Department of Mathematics, University of California, Berkeley, California 94720, USA.*
[3] *Department of Computer Science, University of California, Santa Barbara, California, 93106, USA.*

Contents

Supplementary Information 1. Preliminaries 2
- A Prior art in quantum gate calibration . . . 2
- B Quantum signal processing and polynomial analysis . . . 3
- C Fermionic simulation gate (FsimGate) . . . 4
- D Notation . . . 5

Supplementary Information 2. Details on QSPE in general cases 5

Supplementary Information 3. Analytical structure of periodic circuit 6
- A Exact representation of the periodic circuit . . . 6
- B Approximate Fourier coefficients . . . 9

Supplementary Information 4. Robust estimator against Monte Carlo sampling error 11
- A Modeling the Monte Carlo sampling error . . . 12
- B Statistical estimator against Monte Carlo sampling error . . . 14
- C Improving the estimator of swap angle using the peak information provided by $\hat{\varphi}$ . . . 17
- D Peak regression and peak fitting . . . 21
- E Numerical performance of QSPE on FsimGate against Monte Carlo sampling error . . . 22

Supplementary Information 5. Solving QSPE with arbitrary swap-angle value 22

Supplementary Information 6. Lower bounding the performance of QSPE 24
- A Pre-asymptotic regime $d \ll 1/\theta$ . . . 25
- B Asymptotic regime $d \to \infty$ . . . 28
- C Numerical results . . . 29
- D Comparing with quantum Cramér-Rao lower bound . . . 30

Supplementary Information 7. Analysis of realistic error 32
- A Depolarizing error . . . 33
- B Time-dependent error . . . 33
- C Numerical performance of the estimation against depolarizing error and time-dependent drift error . . . 34
- D Readout error . . . 37
- E Initial state preparation error . . . 38

Supplementary Information 8. Computing the polynomial representation on a special set of points 39

---

* Electronic address: dongyl@berkeley.edu
† Electronic address: murphyniu@ucsb.edu

Supplementary Information 9. Analysis of periodic calibration: variance lower bound and shortcomings 41
A Overview of the methodology of periodic calibration 41
B Optimality analysis using Fisher information and Cramér-Rao bound 42
C Violation of phase-matching condition implies exponentially worse estimation variance 42
D Practical challenges due to the complex optimization landscape 44
Supplementary References 46

Supplementary Information 1. Preliminaries

A. Prior art in quantum gate calibration

A widely used gate calibration technique is called Periodic or Floquet calibration [2, 17], which is an extension of robust phase estimation [12] to multi-parameter regime. It leverages the excitation-preserving structure of the FsimGate to measure the parameters using a restricted set of circuits (compared to full-process tomography). This technique amplifies unitary errors in the gate through repeated applications between measurements. When phase-matching condition is attained, it leads to variance in the estimated parameters that scales inversely with the square of the number of gate applications, thus achieving the Heisenberg limit. In [17], two calibration circuit types are discussed, each utilizing different initialization and measurement techniques. They are referred to as the phase method and the population method. The phase method is able to calibrate the phase accumulation angle. However, given the simpler parametric expression, the population method is more frequently used for calibrating swap angles and phase differences [2, 17]. Consequently, in this paper, we focus on the population method for periodic calibration. The quantum circuit is depicted in Figure 1.

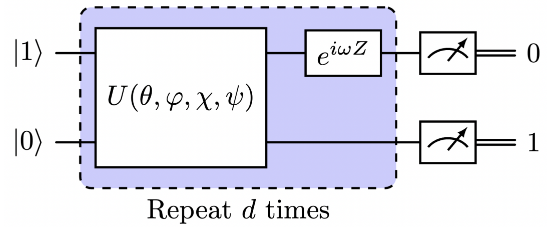

Supplementary Figure 1. Quantum circuit for periodic calibration using the population method.

One difficulty with these techniques is that small values of the swap angle θ are difficult to amplify in the presence of larger single-qubit phases. This can be addressed adaptively, by first measuring the unwanted single-qubit phases and applying compensating pulses, but this strategy is limited by the precision with which one can compensate, and the speed with which these single-qubit phases drift relative to the experiment time. For these reasons, in practice estimation of the swap angle is often done with a depth-1 circuit, commonly referred to as unitary tomography [7].

In Section Supplementary Information 9, we provide a comprehensive analysis of periodic calibration. By bounding the Fisher information, we show that the optimal estimation variance depends heavily on the satisfaction of the phase-matching condition. Though Heisenberg-limit scaling is achieved with perfect phase-matching, realistic errors, e.g. time-dependent drift errors, render the perfect satisfaction of phase-matching condition challenging. According to the analysis, when the swap angle is small, a slight violation of the phase-matching condition can completely undermine the optimal Heisenberg-limit scaling and render the estimation variance exponentially worse in terms of depth dependency. These make the use of periodic calibration impractical when the swap angle is small.

In Figure 2, we demonstrate the experimental estimation of swap angles across multiple runs on a Google Quantum AI superconducting device. The results show that periodic calibration fails to achieve the accuracy needed to reliably distinguish such small gate angles from zero. Moreover, the run-to-run standard deviation in swap-angle estimations varies widely, ranging from 10^{-4} to 10^{-1} radians, with a median of approximately 4×10^{-3} radians. This instability in calibration outcomes stems from the periodic calibration's high sensitivity to time-dependent errors. Linking this to the theoretical analysis, the presence of time-dependent errors also impedes the precise fulfillment of the phase-matching condition, thereby significantly restricting the accuracy of periodic calibration estimations.

An alternative characterization scheme using cross-entropy-benchmarking (XEB) circuits was described in Sec. C. 2. of the supplemental material for [3]. This characterization tool randomizes various noise sources into an effective

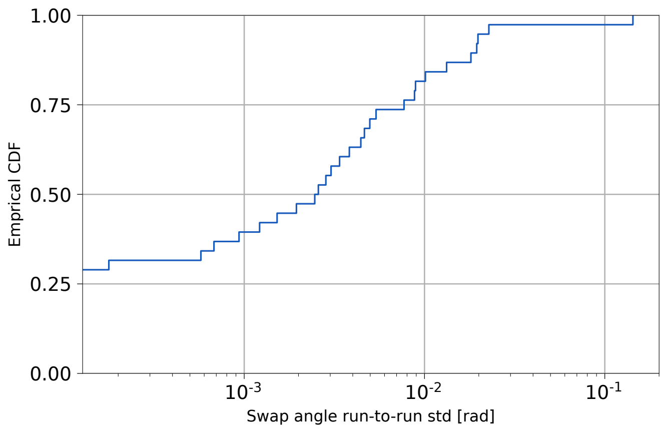

Supplementary Figure 2. Distribution of run-to-run variation of swap-angle estimation across a device. The swap angles were estimated using periodic calibration on four independent datasets for each CZ gate, with 10,000 samples per circuit and a maximum depth of 30. Due to the behavior of the periodic-calibration estimator for particularly small swap angles, a substantial fraction of swap angles were estimated to be identically 0, leading to the portion of the cumulative distribution function that extends off the plot to the left. Discarding these instances leaves us with a median run-to-run standard deviation of close to 4×10^{-3} radians.

depolarizing channel, allowing noise to be simply characterized along with unitary parameters. Randomization comes at a cost, though, requiring a large number of random circuits to get a representative sample of the distribution. Also, randomization interferes with the ability of unitary errors to build up coherently, keeping this method from achieving the Heisenberg limit. This makes it difficult for XEB characterization to resolve angles below 10^{-2} radians in practice.

B. Quantum signal processing and polynomial analysis

The quantum circuit used in QSPE (see Figure 1 in the main text) contains a periodic circuit structure in which the U-gate and a Z-rotation are interleaved. This circuit structure coincides with a quantum algorithm called quantum signal processing (QSP) [8, 14]. QSP is a useful quantum algorithm for solving numerical linear algebra problems such as quantum linear system problems and Hamiltonian simulation by properly choosing a set of phase factors [6, 16]. Specifically, in this paper, we will use the polynomial structure induced by the theory of QSP [8, 14, 21]. Though the quantum circuit used in the paper is a special case of general QSP circuit by fixing all phase modulation angles, the general QSP structure may provide a more robust paradigm against stochastic phase drift errors by relaxing the fixed angle constraint. Hence, for completeness, we provide a concise overview of the theory of QSP structure in this subsection.

The following theorem is a simplified version of [21, Theorem 1].

Theorem 1 (Polynomial structure of symmetric QSP)**.** *Let $d \in \mathbb{N}$ and $\Omega := (\omega_0, \cdots, \omega_d) \in \mathbb{R}^{d+1}$ be a set of phase factors. Then, for any $x \in [-1, 1]$, the following product of* $\mathrm{SU}(2)$*-matrices admits a representation*

$$U(x, \Omega) = e^{\mathrm{i}\omega_0 Z} \prod_{j=1}^{d} \left(e^{\mathrm{i}\arccos(x) X} e^{\mathrm{i}\omega_j Z} \right) = \begin{pmatrix} P(x) & \mathrm{i}Q(x)\sqrt{1-x^2} \\ \mathrm{i}Q^*(x)\sqrt{1-x^2} & P^*(x) \end{pmatrix} \tag{1}$$

for some $P, Q \in \mathbb{C}[x]$ satisfying that

(1) $\deg(P) \leq d, \deg(Q) \leq d-1$,

(2) $P(x)$ *has parity* $(d \mod 2)$ *and* $Q(x)$ *has parity* $(d-1 \mod 2)$,

(3) $|P(x)|^2 + (1-x^2)|Q(x)|^2 = 1, \forall x \in [-1, 1]$.

Here, the superscript $*$ denotes the complex conjugate of a polynomial, namely $P^*(x) = \sum_i \overline{p_i} x^i$ if $P(x) = \sum_i p_i x^i$ with $p_i \in \mathbb{C}$. Furthermore, if Ω is chosen to be symmetric, namely $\omega_j = \omega_{d-j}$ for any j, then $Q \in \mathbb{R}[x]$ is a real polynomial.

Proof. We give a concise proof for completeness.

"Condition (1)": Note that SU(2) matrices satisfy

$$e^{\mathrm{i}\arccos(x)X} = xI + \mathrm{i}\sqrt{1-x^2}X \quad \text{and} \quad Xe^{\mathrm{i}\omega Z} = e^{-\mathrm{i}\omega Z}X.$$

The polynomial representation in Equation (1) follows the expansion and rearranging Pauli X matrices. The condition (1) follows the observation that the leading term is at most x^d when there are even number of Pauli X matrices in the expansion while it is at most x^{d-1} when the number of Pauli X matrices is odd.

"Condition (2)": To see condition (2), we note that under the transformation $x \mapsto -x$, we have

$$e^{\mathrm{i}\arccos(-x)X} = e^{(\pi - \arccos(x))X} = -e^{-\mathrm{i}\arccos(x)X} = -Ze^{\mathrm{i}\arccos(x)X}Z.$$

Therefore

$$U(-x,\mathit{\Omega}) = (-1)^d ZU(x,\mathit{\Omega})Z = \begin{pmatrix} (-1)^d P(x) & \mathrm{i}(-1)^{d-1}Q(x)\sqrt{1-x^2} \\ \mathrm{i}(-1)^{d-1}Q^*(x)\sqrt{1-x^2} & (-1)^d P^*(x) \end{pmatrix}$$

which implies that

$$P(-x) = (-1)^d P(x) \quad \text{and} \quad Q(-x) = (-1)^{d-1}Q(x)$$

which is the parity condition.

"Condition (3)": Condition (3), which is equivalent to $\det U(x,\mathit{\Omega}) = 1$, directly follows the special unitarity.

"Symmetric QSP": Note that when Φ is symmetric, $U(x,\Phi)$ is invariant under the matrix transpose which reverses the order of phase factors. Using $U(x,\Phi) = U(x,\Phi)^\top$, the condition on the polynomial $Q(x) = Q^*(x)$ follows the transformation of the off-diagonal element. Therefore, $Q \in \mathbb{R}[x]$ is a real polynomial. □

The previous theorem bridges the gap between the periodic circuits and the analysis of polynomials. In the paper, we will frequently invoke an important inequality of polynomials to bound error, which is stated as follows.

Theorem 2 (Markov brothers' inequality [15])**.** *Let $P \in \mathbb{R}_d[x]$ be any algebraic polynomial of degree at most d. For any nonnegative integer k, it holds that*

$$\max_{x\in[-1,1]} \left|P^{(k)}(x)\right| \le \max_{x\in[-1,1]} |P(x)| \prod_{j=0}^{k-1} \frac{d^2-j^2}{2j+1}. \tag{2}$$

The equality is attained for Chebyshev polynomial of the first kind $T_d(x)$.

C. Fermionic simulation gate (FsimGate)

A special instance of U-gate is a fermionic simulation gate (FsimGate). It is a class of two-qubit quantum gates preserving the excitation and describes all two-qubit gates realizable in Google's superconducting qubit system. Acting on two qubits A_0 and A_1, the FsimGate is parametrized by a few parameters. Ordering the basis as $\mathcal{B} := \{|00\rangle, |01\rangle, |10\rangle, |11\rangle\}$ where the qubits are ordered as $|a_0a_1\rangle := |a_0\rangle_{A_0}|a_1\rangle_{A_1}$, the unitary matrix representation of the FsimGate is given by

$$U_{\mathrm{FsimGate}}(\theta,\varphi,\chi,\psi,\phi) = \begin{pmatrix} 1 & 0 & 0 & 0 \\ 0 & e^{-\mathrm{i}\varphi-\mathrm{i}\psi}\cos\theta & -\mathrm{i}e^{\mathrm{i}\chi-\mathrm{i}\psi}\sin\theta & 0 \\ 0 & -\mathrm{i}e^{-\mathrm{i}\chi-\mathrm{i}\psi}\sin\theta & e^{\mathrm{i}\varphi-\mathrm{i}\psi}\cos\theta & 0 \\ 0 & 0 & 0 & e^{-\mathrm{i}(\phi+2\psi)} \end{pmatrix}. \tag{3}$$

As a consequence of the preservation of excitation, there is a two-dimensional invariant subspace of the FsimGate, which is referred to as the single-excitation subspace spanned by basis states $\mathcal{B} = \{|01\rangle, |10\rangle\}$. Restricted on the single-excitation subspace $\mathcal{E} := \operatorname{span}\mathcal{B}$, the matrix representation of the FsimGate is (up to a global phase)

$$\begin{aligned} &[U_{\mathrm{FsimGate}}(\theta,\varphi,\chi,\phi,\psi)]_{\mathcal{B}} =: U^{\mathcal{B}}_{\mathrm{FsimGate}}(\theta,\varphi,\chi) \\ &= \begin{pmatrix} e^{-\mathrm{i}\varphi}\cos\theta & -\mathrm{i}e^{\mathrm{i}\chi}\sin\theta \\ -\mathrm{i}e^{-\mathrm{i}\chi}\sin\theta & e^{\mathrm{i}\varphi}\cos\theta \end{pmatrix} = e^{-\mathrm{i}\frac{\varphi-\chi-\pi}{2}Z}e^{\mathrm{i}\theta X}e^{-\mathrm{i}\frac{\varphi+\chi+\pi}{2}Z}. \end{aligned} \tag{4}$$

Here, X and Z are logical Pauli operators by identifying logical quantum states $|0\rangle_\ell := |01\rangle$ and $|1\rangle_\ell := |10\rangle$. As a remark, it provides a parametrization of any general SU(2) matrix.

One of the most important two-qubit quantum gates is controlled-Z gate (CZ). It forms universal gate sets with several single-qubit gates and it is a pivotal building block for demonstrating surface code [1]. CZ is in the gate class of FsimGate's, which can be generated by setting $\theta = \varphi = \chi = \psi = 0$ and $\phi = \pi$. Due to the noisy implementation of CZ, the resulting quantum gate is an FsimGate slightly deviating from the perfect CZ. In order to perform high-fidelity quantum computation, one has to characterize the erroneous parameters of an FsimGate which include CZ as a special case. The characterization of gate parameters relies on quantum phase estimation techniques.

D. Notation

Throughout the paper, M refers to the number of measurement samples unless otherwise noted. For a matrix $A \in \mathbb{C}^{m\times n}$, the transpose, Hermitian conjugate and complex conjugate are denoted by $A^\top$, $A^\dagger$, $\overline{A}$, respectively. The same notations are also used for the operations on a vector. The complex conjugate of a complex number a is denoted as $\overline{a}$. We define the basis kets of the state space of a qubit as follows

$$|0\rangle := \begin{pmatrix} 1 \\ 0 \end{pmatrix}, \quad |1\rangle := \begin{pmatrix} 0 \\ 1 \end{pmatrix}.$$

Supplementary Information 2. Details on QSPE in general cases

In the main text, we provide a quantum circuit for QSPE exemplified by two-qubit U-gates. However, QSPE can be applied to general U-gate with a two-level invariant subspace spanned by logical basis states $\mathcal{B} := \{|0_\ell\rangle, |1_\ell\rangle\}$. Intuitively, we apply a logical Z-rotation to modulate the phase angles in the invariant subspace to form the desired functional form of the output signal. Consequently, to perform the modulation in the invariant subspace, the rotation gate is a logical Z-rotation $e^{\mathrm{i}\omega Z_\ell}$ with the logical Pauli operator defined on the logical basis $Z_\ell = |0_\ell\rangle\langle 0_\ell| - |1_\ell\rangle\langle 1_\ell|$. Then, the matrix representation under the two-level basis states coincides with the conventional Z-ration, namely $[e^{\mathrm{i}\omega Z_\ell}]_{\mathcal{B}} = e^{\mathrm{i}\omega Z}$. The initial state is prepared to two superposition states of logical basis states, which are referred to as logical Bell states: $|+_\ell\rangle := \frac{1}{\sqrt{2}}(|0_\ell\rangle + |1_\ell\rangle)$ and $|\mathrm{i}_\ell\rangle := \frac{1}{\sqrt{2}}(|0_\ell\rangle + \mathrm{i}\,|1_\ell\rangle)$. To read the output signal, we perform a measurement onto the logical state $|0_\ell\rangle$ to measure the transition probability for further analysis. To summarize, the quantum circuit for QSPE in general cases is given in Figure 3.

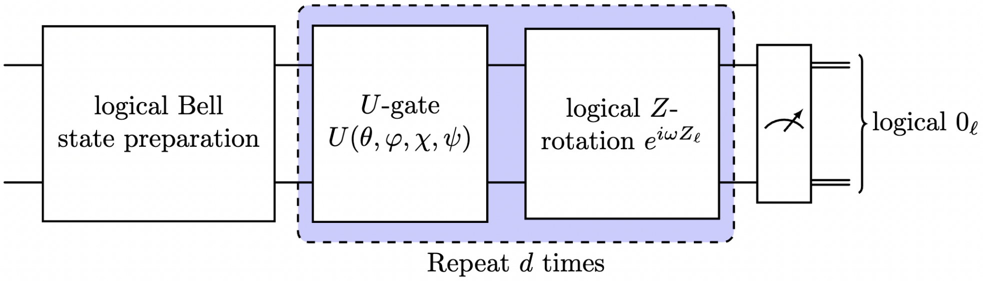

Supplementary Figure 3. Quantum circuit for QSPE in general cases. The input quantum state is prepared to be logical Bell states in either $|+_\ell\rangle$ or $|\mathrm{i}_\ell\rangle$ according to the type of experiment. The quantum circuit enjoys a periodic structure of the unknown (multi-qubit) U-gate and a tunable logical Z-rotation. The measurement is performed to measure the transition probability onto the logical basis state $|0_\ell\rangle$.

In the invariant subspace spanned by $\mathcal{B}$, the matrix representation of the periodic part of the circuit is

$$\mathcal{U}^{(d)}(\omega;\theta,\varphi,\chi) = \left[\left(e^{\mathrm{i}\omega Z_\ell} U(\theta,\varphi,\chi,\psi)\right)^d\right]_{\mathcal{B}} = e^{-\mathrm{i}d\psi}\left(e^{\mathrm{i}\omega Z} e^{-\mathrm{i}\frac{\varphi-\chi-\pi}{2}Z} e^{\mathrm{i}\theta X} e^{-\mathrm{i}\frac{\varphi+\chi+\pi}{2}Z}\right)^d. \tag{5}$$

Here, the Euler-angle decomposition of the two-dimensional unitary $[U(\theta,\varphi,\chi,\psi)]_{\mathcal{B}}$ is used. We also remark that the ψ-dependency in the unitary representation is hidden for simplicity because the global phase $e^{-\mathrm{i}d\psi}$ does not affect measurement probability. In the later presentation, we will drop the global phase or mark it as $\psi \leftarrow *$ for simplicity.

Note that the adjacent Z-rotations can be combined to simplify the expression. Hence, the unitary representation is

$$\mathcal{U}^{(d)}(\omega;\theta,\varphi,\chi)=e^{\mathrm{i}\frac{\chi+\pi+\varphi}{2}Z}\left(e^{\mathrm{i}(\omega-\varphi)Z}e^{\mathrm{i}\theta X}\right)^{d}e^{-\mathrm{i}\frac{\chi+\pi+\varphi}{2}Z}. \tag{6}$$

It is worth noting that the periodic part is independent of the phase accumulation angle χ. Consequently, despite the amplification of θ and φ, the angle χ is not amplified on this basis. Hence, the estimation accuracy of χ is not significantly affected by increasing the depth parameter d in the quantum circuit outlined in Figure 3. To conclude, Equation (6) will be the starting point of our analysis in the later section.

Supplementary Information 3. Analytical structure of periodic circuit

The QSPE circuit in Figure 1 in the main text (or Figure 3) enjoys a periodic structure by interleaving U-gate and Z-rotation. This periodic structure is studied by the theory of QSP (Theorem 1). Consequentially, the QSPE circuit admits some polynomial representation. In this section, we will derive the analytical form of the structure of the QSPE circuit. We start from the exact closed-form results of the QSPE circuit in Section Supplementary Information 3 A. In Section Supplementary Information 3 B, we derive a good approximation to the closed-form exact results. The analysis in this section proves the following theorem which summarizes findings about the structure of QSPE.

Theorem 3 (Structure of QSPE). *Let $d\in\mathbb{N}$ be the number of U-gate applications in the QSPE circuit, and*

$$\mathfrak{h}(\omega;\theta,\varphi,\chi):=p_X(\omega;\theta,\varphi,\chi)-\frac{1}{2}+\mathrm{i}\left(p_Y(\omega;\theta,\varphi,\chi)-\frac{1}{2}\right) \tag{7}$$

be the reconstructed function derived from the measurement probability. Then, it admits a finite Fourier series expansion

$$\mathfrak{h}(\omega;\theta,\varphi,\chi)=\sum_{-d+1}^{d-1}c_k(\theta,\varphi,\chi)e^{2\mathrm{i}k\omega}. \tag{8}$$

Furthermore, for nonnegative indices $k=0,1,\cdots,d-1$, the Fourier coefficients take the form

$$c_k(\theta,\chi,\varphi)=\mathrm{i}e^{-\mathrm{i}\chi}e^{-\mathrm{i}(2k+1)\varphi}\theta+\max\left\{\mathcal{O}\left(\theta^3\right),\mathcal{O}\left((d\theta)^5\right)\right\}. \tag{9}$$

A. Exact representation of the periodic circuit

We abstract a simple SU(2)-product model which can be shown as the building block of the QSPE circuit in Figure 1 in the main text (or Figure 3). It turns out that the model admits a polynomial representation.

Definition 4 (Building block of QSPE). *Let $\theta,\omega\in\mathbb{R}$ be any angles, $d\in\mathbb{N}$ by any positive integer. Then, the matrix representation of a periodic circuit with d repetitions and Z-phase modulation angle ω is*

$$U^{(d)}(\omega,\theta)=\left(e^{\mathrm{i}\omega Z}e^{\mathrm{i}\theta X}\right)^{d}e^{\mathrm{i}\omega Z}. \tag{10}$$

In the quantum circuit defined above, the X- and Z-rotations are interleaved, which agrees with the structure of QSP in Theorem 1. The theory of QSP implies that the SU(2)-product model enjoys a structure represented by polynomials which is given by the following lemma.

Lemma 5. *Let $x=\cos(\theta)\in[-1,1]$. There exists a complex polynomial $P_\omega^{(d)}\in\mathbb{C}_d[x]$ and a real polynomial $Q_\omega^{(d)}\in\mathbb{R}_{d-1}[x]$ so that*

$$U^{(d)}\left(\omega,\arccos(x)\right)=\begin{pmatrix}P_\omega^{(d)}(x) & \mathrm{i}\sqrt{1-x^2}Q_\omega^{(d)}(x)\\ \mathrm{i}\sqrt{1-x^2}Q_\omega^{(d)*}(x) & P_\omega^{(d)*}(x)\end{pmatrix}. \tag{11}$$

Furthermore, the special unitarity of $U^{(d)}(\omega,\arccos(x))$ yields

$$P_\omega^{(d)}(x)P_\omega^{(d)*}(x)+(1-x^2)\left(Q_\omega^{(d)}(x)\right)^2=1. \tag{12}$$

Proof. Following [8, Theorem 4], there exists two polynomials $P_\omega^{(d)}, Q_\omega^{(d)} \in \mathbb{C}[x]$ so that Equation (11) holds. Because $U^{(d)}(\omega, \arccos(x))$ is a QSP unitary with a set of symmetric phase factors, $Q_\omega^{(d)} \in \mathbb{R}_{d-1}[x]$ is a real polynomial according to [6, Theorem 2]. Equation (12) holds by taking the determinant of Equation (11). □

The exact presentation of the pair of polynomials $(P_\omega^{(d)}, Q_\omega^{(d)})$ can be determined via recurrence on a special set of points $d = 2^j, j \in \mathbb{N}$ (see Lemma 20 in Appendix). Based on it, we prove the generalized result to any positive integer d by using induction. This gives a complete characterization of the structure of the SU(2)-product model in Definition 4.

Theorem 6. *Let $d = 1, 2, \ldots$ be any positive integer. Then*

$$P_\omega^{(d)}(x) = e^{\mathrm{i}\omega}\left(\cos(d\sigma) + \mathrm{i}\frac{\sin(d\sigma)}{\sin\sigma}(\sin\omega)\, x\right) \text{ and } Q_\omega^{(d)}(x) = \frac{\sin(d\sigma)}{\sin\sigma} \tag{13}$$

where $\sigma = \arccos((\cos\omega)\, x)$.

Proof. Let us prove the theorem by induction. The base case is $d = 1$, where $P_\omega^{(1)}(x) = e^{2\mathrm{i}\omega}x$ and $Q_\omega^{(1)}(x) = 1$. Assuming that the induction hypothesis holds for d, we will prove it also holds for $d+1$. Using Definition 4 and Lemma 5, the polynomials can be determined by a recurrence relation

$$U^{(d+1)}(\omega,\theta) = U^{(d)}(\omega,\theta)e^{\mathrm{i}\theta X}e^{\mathrm{i}\omega Z} \Rightarrow \begin{cases} P_\omega^{(d+1)}(x) = e^{\mathrm{i}\omega}\left(xP_\omega^{(d)}(x) - (1-x^2)Q_\omega^{(d)}(x)\right), \\ Q_\omega^{(d+1)}(x) = e^{-\mathrm{i}\omega}\left(P_\omega^{(d)}(x) + xQ_\omega^{(d)}(x)\right). \end{cases} \tag{14}$$

Using the induction hypothesis, we have

$$\begin{aligned} P_\omega^{(d+1)}(x) &= e^{\mathrm{i}\omega}\Bigg(\cos\sigma\cos(d\sigma) - \left(1 - \left(1-\sin^2\omega\right)x^2\right)\frac{\sin(d\sigma)}{\sin\sigma} \\ &\qquad + \mathrm{i}(\sin\omega)\, x\frac{\sin\sigma\cos(d\sigma) + \cos\sigma\sin(d\sigma)}{\sin\sigma}\Bigg) \\ &= e^{\mathrm{i}\omega}\left(\cos((d+1)\sigma) + \mathrm{i}\frac{\sin((d+1)\sigma)}{\sin\sigma}(\sin\omega)\, x\right) \end{aligned} \tag{15}$$

and

$$Q_\omega^{(d+1)}(x) = \cos(d\sigma) + \cos\sigma\frac{\sin(d\sigma)}{\sin\sigma} = \frac{\sin((d+1)\sigma)}{\sin\sigma}. \tag{16}$$

Therefore, the theorem follows induction. □

The closed-form results above help us to analyze the dynamics of Figure 1 in the main text (or Figure 3) where we apply a Pauli Z modulation $e^{\mathrm{i}\omega Z_{A_0}}$ to the periodic circuit. Restricted to the single-excitation subspace, the matrix representation of the QSPE circuit in Figure 1 in the main text (or Figure 3) is

$$\mathcal{U}^{(d)}(\omega;\theta,\varphi,\chi) = \left[\left(e^{\mathrm{i}\omega Z_\ell}U(\theta,\varphi,\chi,*)\right)^d\right]_{\mathcal{B}} = e^{\mathrm{i}\frac{\chi+\pi+\varphi}{2}Z}U^{(d)}(\omega-\varphi,\theta)e^{-\mathrm{i}\left(\omega+\frac{\chi+\pi-\varphi}{2}\right)Z}. \tag{17}$$

The initial two-qubit state of the QSP circuit can be prepared as Bell states $|+_\ell\rangle$ or $|\mathrm{i}_\ell\rangle$ by using Hadamard gate, phase gate and CNOT gate. Recall that we denote the probability by measuring qubits A_0A_1 with 01 as

$$p_X(\omega;\theta,\varphi,\chi) = \left|\langle 0_\ell|\,\mathcal{U}^{(d)}(\omega;\theta,\varphi,\chi)\,|+_\ell\rangle\right|^2 \tag{18}$$

when the initial state is $|+_\ell\rangle$, and

$$p_Y(\omega;\theta,\varphi,\chi) = \left|\langle 0_\ell|\,\mathcal{U}^{(d)}(\omega;\theta,\varphi,\chi)\,|\mathrm{i}_\ell\rangle\right|^2 \tag{19}$$

when the initial state is $|\mathrm{i}_\ell\rangle$ respectively. These bridge the gap between the analytical results derived based on Definition 4 and the measurement probabilities from the experimental setting. We are ready to prove the first half of Theorem 3.

Theorem 7. *The function reconstructed from the measurement probability admits the following Fourier series expansion:*

$$\mathfrak{h}(\omega;\theta,\varphi,\chi) := p_X(\omega;\theta,\varphi,\chi) + \mathrm{i}p_Y(\omega;\theta,\varphi,\chi) - \frac{1+\mathrm{i}}{2} = \sum_{k=-d+1}^{d-1} c_k(\theta,\chi,\varphi)e^{2\mathrm{i}k\omega} \tag{20}$$

where

$$c_k(\theta,\chi,\varphi) = \mathrm{i}e^{-\mathrm{i}\chi}e^{-\mathrm{i}(2k+1)\varphi}\widetilde{c}_k(\theta) \quad \textit{and} \quad \widetilde{c}_k(\theta) \in \mathbb{R}. \tag{21}$$

Proof. For simplicity, let $\beta \in \mathrm{U}(1)$ and $|\beta\rangle := \frac{1}{\sqrt{2}}\left(|0_\ell\rangle + \beta\,|1_\ell\rangle\right)$. Then, $|\beta = 1\rangle = |+_\ell\rangle$ and $|\beta = \mathrm{i}\rangle = |\mathrm{i}_\ell\rangle$. Given the input quantum state is $|\beta\rangle$, we have the measurement probability

$$\begin{aligned} p_\beta(\omega;\theta,\varphi) &= \left|\langle 0_\ell|\,\mathcal{U}^{(d)}(\omega;\theta,\varphi,\chi)\,|\beta\rangle\right|^2 \\ &= \left|\frac{1}{\sqrt{2}}e^{\mathrm{i}\frac{\varphi+\chi+\pi}{2}}\langle 0_\ell|\,U^{(d)}(\omega-\varphi,\theta)\left(e^{-\mathrm{i}\left(\omega+\frac{\chi+\pi-\varphi}{2}\right)}|0_\ell\rangle + \beta e^{\mathrm{i}\left(\omega+\frac{\chi+\pi-\varphi}{2}\right)}|1_\ell\rangle\right)\right|^2 \\ &= \frac{1}{2} + \mathrm{Re}\left(\overline{\beta}e^{\mathrm{i}(\varphi-\chi-2\omega)}P^{(d)}_{\omega-\varphi}(\cos\theta)\mathrm{i}\sin\theta Q^{(d)}_{\omega-\varphi}(\cos\theta)\right). \end{aligned} \tag{22}$$

Then, $p_X = p_{\beta=1}$ and $p_Y = p_{\beta=\mathrm{i}}$. Furthermore, it holds that

$$\begin{aligned} p_X(\omega;\theta,\varphi,\chi) - \frac{1}{2} &= \mathrm{Re}\left(e^{\mathrm{i}(\varphi-\chi-2\omega)}P^{(d)}_{\omega-\varphi}(\cos\theta)\mathrm{i}\sin\theta Q^{(d)}_{\omega-\varphi}(\cos\theta)\right), \\ p_Y(\omega;\theta,\varphi,\chi) - \frac{1}{2} &= \mathrm{Im}\left(e^{\mathrm{i}(\varphi-\chi-2\omega)}P^{(d)}_{\omega-\varphi}(\cos\theta)\mathrm{i}\sin\theta Q^{(d)}_{\omega-\varphi}(\cos\theta)\right). \end{aligned} \tag{23}$$

Therefore, the reconstructed function is

$$\mathfrak{h}(\omega;\theta,\varphi,\chi) = \mathrm{i}e^{-\mathrm{i}(\chi+\varphi)}\sin\theta e^{-2\mathrm{i}(\omega-\varphi)}P^{(d)}_{\omega-\varphi}(\cos\theta)Q^{(d)}_{\omega-\varphi}(\cos\theta) =: \mathrm{i}e^{-\mathrm{i}(\chi+\varphi)}\widetilde{\mathfrak{h}}(\omega-\varphi,\theta). \tag{24}$$

Note that following Theorem 6

$$\begin{aligned} &P^{(d)}_{\omega+\pi-\varphi}(\cos\theta) = (-1)^{d+1}P^{(d)}_{\omega-\varphi}(\cos\theta),\ Q^{(d)}_{\omega+\pi-\varphi}(\cos\theta) = (-1)^{d-1}Q^{(d)}_{\omega-\varphi}(\cos\theta) \\ &\Rightarrow\ \widetilde{\mathfrak{h}}(\omega+\pi-\varphi,\theta) = \widetilde{\mathfrak{h}}(\omega-\varphi,\theta). \end{aligned} \tag{25}$$

That means $\widetilde{\mathfrak{h}}(\omega-\varphi,\theta)$ is π-periodic in the first argument. Furthermore, $\widetilde{\mathfrak{h}}(\omega-\varphi,\theta)$ is a trigonometric polynomial in $(\omega-\varphi)$. Thus, it admits the Fourier series expansion:

$$\widetilde{\mathfrak{h}}(\omega-\varphi,\theta) = \sum_{k=-d+1}^{d-1}\widetilde{c}_k(\theta)e^{2\mathrm{i}k(\omega-\varphi)} \tag{26}$$

with coefficients

$$\widetilde{c}_k(\theta) = \frac{\sin\theta}{\pi}\int_0^\pi e^{-2\mathrm{i}(k+1)\omega}P^{(d)}_\omega(\cos\theta)Q^{(d)}_\omega(\cos\theta)\mathrm{d}\omega. \tag{27}$$

The upper limit and lower limit of the summation index $\pm(d-1)$ can be verified by straightforward computation. According to Theorem 6, we also have

$$P^{(d)}_{-\omega}(\cos\theta) = \overline{P^{(d)}_\omega(\cos\theta)},\ Q^{(d)}_\omega(\cos\theta) \in \mathbb{R}\ \Rightarrow\ \widetilde{\mathfrak{h}}(\omega,\theta) = \overline{\widetilde{\mathfrak{h}}(-\omega,\theta)}\ \Rightarrow\ \widetilde{c}_k(\theta) \in \mathbb{R}. \tag{28}$$

The proof is completed. □

It is also useful to study the magnitude of the reconstructed function. It gives the intuition of the distribution of the magnitude over different modulation angle ω. The following corollary indicated that the magnitude of the reconstructed function attains its maximum $d\theta$ when the phase matching condition $\omega = \varphi$ is achieved.

Corollary 8. *The magnitude of* $p_X(\omega;\theta,\varphi,\chi)-\frac{1}{2}$ *and* $p_Y(\omega;\theta,\varphi,\chi)-\frac{1}{2}$ *are of order* $\sin\theta$*. Furthermore*

$$\begin{aligned}\mathfrak{p}(\omega-\varphi,\theta) &:= |\mathfrak{h}(\omega;\theta,\varphi,\chi)|^2 = \left(p_X(\omega;\theta,\varphi,\chi)-\frac{1}{2}\right)^2+\left(p_Y(\omega;\theta,\varphi,\chi)-\frac{1}{2}\right)^2\\ &= \sin^2(\theta)\frac{\sin^2(d\sigma)}{\sin^2(\sigma)}\left(1-\sin^2(\theta)\frac{\sin^2(d\sigma)}{\sin^2(\sigma)}\right).\end{aligned} \tag{29}$$

Here $\sigma=\arccos\left(\cos(\omega-\varphi)\cos(\theta)\right)$.

Proof. Using Theorem 6 and Equations (12) and (23) as intermediate steps, we have

$$\begin{aligned}\mathfrak{p}(\omega-\varphi,\theta) &= \left|e^{\mathrm{i}(\varphi-\chi-2\omega)}P^{(d)}_{\omega-\varphi}(\cos\theta)\mathrm{i}\sin\theta Q^{(d)}_{\omega-\varphi}(\cos\theta)\right|^2\\ &= \sin^2(\theta)\left|Q^{(d)}_{\omega-\varphi}(\cos\theta)\right|^2\left|P^{(d)}_{\omega-\varphi}(\cos\theta)\right|^2\\ &= \sin^2(\theta)\left|Q^{(d)}_{\omega-\varphi}(\cos\theta)\right|^2\left(1-\sin^2(\theta)\left|Q^{(d)}_{\omega-\varphi}(\cos\theta)\right|^2\right)\\ &= \sin^2(\theta)\frac{\sin^2(d\sigma)}{\sin^2(\sigma)}\left(1-\sin^2(\theta)\frac{\sin^2(d\sigma)}{\sin^2(\sigma)}\right)\end{aligned} \tag{30}$$

which completes the proof. □

Notice that if the transition probability between tensor-product states is measured, the magnitude of the signal (the nontrivial θ dependence in the transition probability) is $\mathcal{O}\left(\sin^2\theta\right)$. Nonetheless, by preparing the input quantum state as Bell states, Corollary 8 reveals that the magnitude of the signal is lifted to $\mathcal{O}(\sin\theta)$ instead. Therefore, when θ is small, it is a significant improvement of the SNR especially in the presence of realistic errors.

B. Approximate Fourier coefficients

Theorem 7 shows that the θ and φ dependence are factored completely in the amplitude and the phase of the Fourier coefficients of the reconstructed function $\mathfrak{h}$ respectively. Given the angle ω of the Z-rotation is tunable, we can sample the data point by performing the QSPE circuit in Figure 1 in the main text (or Figure 3) with equally spaced angles $\omega_j=\frac{j}{2d-1}\pi$ where $j=0,\cdots,2d-2$. These $2(2d-1)$ quantum experiments yield two sequences of measurement probabilities $\mathbf{p}_X^{\mathrm{exp}}:=(p_X^{\mathrm{exp}}(\omega_0),p_X^{\mathrm{exp}}(\omega_1),\cdots,p_X^{\mathrm{exp}}(\omega_{2d-2}))$ and $\mathbf{p}_Y^{\mathrm{exp}}:=(p_Y^{\mathrm{exp}}(\omega_0),p_Y^{\mathrm{exp}}(\omega_1),\cdots,p_Y^{\mathrm{exp}}(\omega_{2d-2}))$. Therefore, we can compute $\mathfrak{h}^{\mathrm{exp}}=\mathbf{p}_X^{\mathrm{exp}}+\mathbf{p}_Y^{\mathrm{exp}}-\frac{1+\mathrm{i}}{2}$ from experimental data. The Fourier coefficients of $\mathfrak{h}$ can be computed by fast Fourier transform (FFT). The Fourier coefficients $\mathbf{c}^{\mathrm{exp}}:=\left(c^{(\mathrm{exp})}_{-d+1},c^{(\mathrm{exp})}_{-d+2},\cdots,c^{(\mathrm{exp})}_{d-1}\right)=\mathsf{FFT}(\mathfrak{h}^{\mathrm{exp}})$ can be computed efficiently using FFT. In order to infer θ and φ accurately and efficiently from the data, we need to study the approximate structure of the Fourier coefficients first.

Theorem 9. *Let* $\hat{\mathfrak{h}}(\omega,\cos\theta):=\widetilde{\mathfrak{h}}(\omega,\theta)/(\sin\theta e^{-\mathrm{i}\omega})$*. There is an approximation to it:*

$$\begin{aligned}&\hat{\mathfrak{h}}^\star(\omega,\cos\theta)=\sum_{k=-d+1}^{d-1}\hat{c}^\star_k(\theta)e^{\mathrm{i}(2k+1)\omega}, \quad \textit{where}\\ &\hat{c}^\star_k(\theta)=\begin{cases}1-\frac{1}{2}\left(3d^2-k^2-(k+1)^2-(d-(2k+1))^2\right)(1-\cos\theta) & \textit{if } 0\le k\le d-1,\\ -\frac{1}{2}\left(d^2+(d+2k+1)^2-k^2-(k+1)^2\right)(1-\cos\theta) & \textit{if } -d+1\le k\le -1.\end{cases}\end{aligned} \tag{31}$$

The approximation error is upper bounded as

$$\max_{\omega\in[0,\pi]}\left|\hat{\mathfrak{h}}(\omega,\cos\theta)-\hat{\mathfrak{h}}^\star(\omega,\cos\theta)\right|\le 2d^5\theta^4 \tag{32}$$

and for any k

$$|\widetilde{c}_k(\theta)-\sin\theta\hat{c}^\star_k(\theta)|\le 2(d\theta)^5. \tag{33}$$

Proof. Following Theorem 6, we have

$$\begin{aligned}\widetilde{\mathfrak{h}}(\omega,\theta) &= \sin\theta e^{-2\mathrm{i}\omega} P_\omega^{(d)}(\cos\theta) Q_\omega^{(d)}(\cos\theta) = \sin\theta e^{-\mathrm{i}\omega}\left(\cos(d\sigma) + \mathrm{i}\sin\omega\cos\theta\frac{\sin(d\sigma)}{\sin\sigma}\right)\frac{\sin(d\sigma)}{\sin\sigma} \\ &= \sin\theta e^{-\mathrm{i}\omega}\left(T_d(\cos\sigma) + \mathrm{i}\sin\omega\cos\theta U_{d-1}(\cos\sigma)\right) U_{d-1}(\cos\sigma)\end{aligned} \tag{34}$$

where $\cos\sigma = \cos\omega\cos\theta$ and $T_d \in \mathbb{R}_d[x], U_{d-1} \in \mathbb{R}_{d-1}[x]$ are Chebyshev polynomials of the first and second kind respectively. Then

$$\begin{aligned}\hat{\mathfrak{h}}(\omega,\cos\theta) &:= \left(T_d(\cos\sigma) + \mathrm{i}\sin\omega\cos\theta U_{d-1}(\cos\sigma)\right) U_{d-1}(\cos\sigma) \\ &= \frac{1}{2}U_{2d-1}(\cos\sigma) + \mathrm{i}\sin\omega\cos\theta U_{d-1}^2(\cos\sigma).\end{aligned} \tag{35}$$

Therefore, for a given ω, $\hat{\mathfrak{h}}(\omega,\cos\theta)$ is a polynomial in $\cos\theta$ of degree at most $2d-1$. According to Corollary 8, we have for any ω

$$\max_{\theta\in[0,\pi]} \max\left\{\left|\mathrm{Re}\left(\hat{\mathfrak{h}}(\omega,\cos\theta)\right)\right|, \left|\mathrm{Im}\left(\hat{\mathfrak{h}}(\omega,\cos\theta)\right)\right|\right\} \le \max_{\theta\in[0,\pi]}\left|\hat{\mathfrak{h}}(\omega,\cos\theta)\right| \le \max_{\theta\in[0,\pi]}\left|\frac{\sin(d\sigma)}{\sin\sigma}\right| \le d. \tag{36}$$

Applying Taylor's theorem and expanding $\hat{\mathfrak{h}}(\omega,\cos\theta)$ with respect to $1-\cos\theta$, there exists $\xi\in(\cos\theta,1)$ so that

$$\hat{\mathfrak{h}}(\omega,\cos\theta) = \hat{\mathfrak{h}}(\omega,1) + \left.\frac{\partial\hat{\mathfrak{h}}(\omega,x)}{\partial x}\right|_{x=1}(\cos\theta-1) + \frac{1}{2}\left.\frac{\partial^2\hat{\mathfrak{h}}(\omega,x)}{\partial x^2}\right|_{x=\xi}(\cos\theta-1)^2. \tag{37}$$

Here

$$\hat{\mathfrak{h}}(\omega,1) = e^{\mathrm{i}d\omega}U_{d-1}(\cos\omega) = e^{\mathrm{i}\omega}\sum_{k=0}^{d-1} e^{2\mathrm{i}k\omega}. \tag{38}$$

Furthermore,

$$\begin{aligned}\mathrm{Re}\left(\left.\frac{\partial\hat{\mathfrak{h}}(\omega,x)}{\partial x}\right|_{x=1}\right) &= \frac{1}{2}\left.\frac{\partial U_{2d-1}(x\cos\omega)}{\partial x}\right|_{x=1} = \frac{\cos\omega}{2}U'_{2d-1}(\cos\omega) \\ &= \cos\omega\sum_{j=0}^{d-1}(2j+1)U_{2j}(\cos\omega) = \cos\omega\sum_{j=0}^{d-1}(2j+1)\sum_{k=-j}^{j} e^{2\mathrm{i}k\omega} \\ &= \cos\omega\sum_{k=-(d-1)}^{d-1}(d^2-k^2)e^{2\mathrm{i}k\omega}\end{aligned} \tag{39}$$

and

$$\begin{aligned}\mathrm{Im}\left(\left.\frac{\partial\hat{\mathfrak{h}}(\omega,x)}{\partial x}\right|_{x=1}\right) &= \sin\omega U_{d-1}(\cos\omega)\left(U_{d-1}(\cos\omega) + 2\cos\omega U'_{d-1}(\cos\omega)\right) \\ &= \sin(d\omega)\left(\sum_{\substack{k=-(d-1)\\ \text{stepsize } 2}}^{d-1} e^{\mathrm{i}k\omega} + \frac{1}{2}\left(e^{\mathrm{i}\omega}+e^{-\mathrm{i}\omega}\right)\sum_{\substack{k=-(d-2)\\ \text{stepsize } 2}}^{d-2}(d^2-k^2)e^{\mathrm{i}k\omega}\right) \\ &= \sin(d\omega)\sum_{\substack{k=-(d-1)\\ \text{stepsize } 2}}^{d-1}(d^2-k^2)e^{\mathrm{i}k\omega} = \frac{1}{2\mathrm{i}}\left(\sum_{\substack{k=-2d+1\\ \text{stepsize } 2}}^{-1}(2d+k)ke^{\mathrm{i}k\omega} + \sum_{\substack{k=1\\ \text{stepsize } 2}}^{2d-1}(2d-k)ke^{\mathrm{i}k\omega}\right).\end{aligned} \tag{40}$$

Let the approximation of $\hat{\mathfrak{h}}(\omega,\cos\theta)$ be

$$\hat{\mathfrak{h}}^\star(\omega,\cos\theta) := \hat{\mathfrak{h}}(\omega,1) + \left.\frac{\partial\hat{\mathfrak{h}}(\omega,x)}{\partial x}\right|_{x=1}(\cos\theta-1). \tag{41}$$

Then, the previous computation shows it admits a Fourier series expansion:

$$\hat{\mathfrak{h}}^\star(\omega,\cos\theta) = \sum_{k=-d+1}^{d-1} \hat{c}_k^\star(\theta) e^{\mathrm{i}(2k+1)\omega}, \text{ where}$$
$$\hat{c}_k^\star(\theta) = \begin{cases} 1+\frac{1}{2}\left(3d^2-k^2-(k+1)^2-(d-(2k+1))^2\right)(\cos\theta-1) & \text{if } 0\le k\le d-1, \\ \frac{1}{2}\left(d^2+(d+2k+1)^2-k^2-(k+1)^2\right)(\cos\theta-1) & \text{if } -d+1\le k\le -1. \end{cases} \tag{42}$$

The approximation error can be bounded by using Equation (37). For any $\omega\in[0,\pi]$, we have

$$\begin{aligned} \left|\mathfrak{h}(\omega,\cos\theta)-\hat{\mathfrak{h}}^\star(\omega,\cos\theta)\right| &\le \frac{(1-\cos\theta)^2}{2}\max_{x\in[-1,1]}\left|\frac{\partial^2\hat{\mathfrak{h}}(\omega,x)}{\partial x^2}\right| \\ &\le \frac{\theta^4}{8}\sqrt{\left(\max_{x\in[-1,1]}\left|\frac{\partial^2 \mathrm{Re}\left(\hat{\mathfrak{h}}(\omega,x)\right)}{\partial x^2}\right|\right)^2+\left(\max_{x\in[-1,1]}\left|\frac{\partial^2 \mathrm{Im}\left(\hat{\mathfrak{h}}(\omega,x)\right)}{\partial x^2}\right|\right)^2}. \end{aligned} \tag{43}$$

Note that $\mathrm{Re}\left(\hat{\mathfrak{h}}(\omega,x)\right)$ and $\mathrm{Im}\left(\hat{\mathfrak{h}}(\omega,x)\right)$ are real polynomials in x of degree at most $2d-1$. Invoking the Markov brothers' inequality (Theorem 2), we further get

$$\begin{aligned} &\left|\mathfrak{h}(\omega,\cos\theta)-\hat{\mathfrak{h}}^\star(\omega,\cos\theta)\right| \\ &\le \frac{\sqrt{2}(2d-1)^2 d(d-1)}{6}\theta^4 \max_{x\in[-1,1]}\max\left\{\left|\mathrm{Re}\left(\hat{\mathfrak{h}}(\omega,x)\right)\right|,\left|\mathrm{Im}\left(\hat{\mathfrak{h}}(\omega,x)\right)\right|\right\} \\ &\le \frac{\sqrt{2}(2d-1)^2 d^2(d-1)}{6}\theta^4 \le 2d^5\theta^4, \end{aligned} \tag{44}$$

where Equation (36) is used. The error bound can be transferred to that of the Fourier coefficients. Using the previous result and triangle inequality, one has

$$\begin{aligned} \left|\widetilde{c}_k(\theta)-\sin\theta\hat{c}_k^\star(\theta)\right| &= \left|\frac{\sin\theta}{\pi}\int_0^\pi e^{-\mathrm{i}(2k+1)\omega}\left(\hat{\mathfrak{h}}(\omega,\cos\theta)-\hat{\mathfrak{h}}^\star(\omega,\cos\theta)\right)\mathrm{d}\omega\right| \\ &\le \sin\theta\frac{1}{\pi}\int_0^\pi\left|\hat{\mathfrak{h}}(\omega,\cos\theta)-\hat{\mathfrak{h}}^\star(\omega,\cos\theta)\right|\mathrm{d}\omega \le 2\,(d\theta)^5. \end{aligned} \tag{45}$$

The proof is completed. □

There are two implications of the previous theorem. First, it suggests that the magnitude of the Fourier coefficients of negative indices are $\mathcal{O}\left(\sin^3\theta\right)$. If they are included in the formalism of the inference problem in the Fourier space, the accuracy of inference may be heavily contaminated because of the nearly vanishing SNR when $\theta\ll 1$. On the other hand, the amplitude of the Fourier coefficients of nonnegative indices tightly concentrates at $\sin\theta$ when $\theta\ll 1$. Therefore, a nice linear approximation of the Fourier coefficients holds in the case of small swap angle: for any $k=0,\cdots,d-1$

$$c_k(\theta,\chi,\varphi) = \mathrm{i}e^{-\mathrm{i}\chi}e^{-\mathrm{i}(2k+1)\varphi}\theta + \max\left\{\mathcal{O}\left(\theta^3\right),\mathcal{O}\left((d\theta)^5\right)\right\}. \tag{46}$$

This proves the second half of Theorem 3.

To numerically demonstrate the previous theorem, we depicted the exactly computed Fourier coefficients and their approximation in Figure 4. The numerical results support that the approximated coefficients are close to the exact ones when $d\theta\le 1$. Furthermore, the vanishing values of negatively indexed Fourier coefficients and the approximately linear growth of nonnegatively indexed Fourier coefficients are visualized in the figure.

Supplementary Information 4. Robust estimator against Monte Carlo sampling error

The estimation problem of QSPE is formalized as follows.

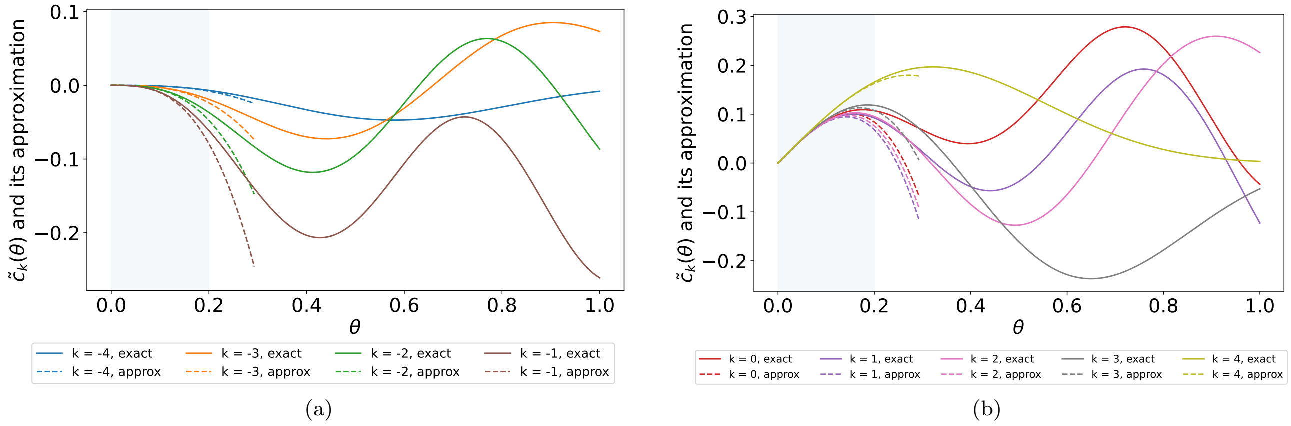

Supplementary Figure 4. Numerical justification of the approximated Fourier coefficients. We set $d = 5$. The solid curve is computed by integrating the Fourier transformation. The dashed curve is derived from the approximation in the previous theorem. The shaded area stands for $d\theta \leq 1$. (a) Fourier coefficients of negative indices. (b) Fourier coefficient of nonnegative indices.

Problem 10 (Calibrating U-gate using QSPE)**.** *(1) QSPE: Given experimentally measured probabilities of QSPE circuits in Figure 1 in the main text (or Figure 3) on $\{\omega_j : j = 0, \cdots, 2d-2\}$, infer θ and φ accurately.*

(2) QSPE in Fourier space: Given experimentally measured Fourier coefficients of nonnegative indices, infer θ and φ accurately.

A dominant and unavoidable source of errors in quantum metrology is Monte Carlo sampling error due to the finite sample size in quantum measurements. Such limitation derives from both practical concerns of the efficiency of quantum metrology, and realistic constraints where some system parameters can drift over time and can only be monitored by sufficiently fast protocols. In this section, we analyze the effect of Monte Carlo sampling error in our proposed metrology algorithm by characterizing the sampling error as a function of quantum circuit depth, FsimGate parameters and sample size. The result will also be used in Section Supplementary Information 6 to prove that our estimator based on QSPE is optimal. In the following analysis, we annotate with superscript "exp" to represent experimentally measured probability as opposed to expected probability from theory.

A. Modeling the Monte Carlo sampling error

We start the analysis by statistically modeling the Monte Carlo sampling error on the measurement probabilities. Furthermore, we also derive the sampling error induced on the Fourier coefficients derived from experimental data. The result is summarized in the following lemma.

Lemma 11. *Let M be the number of measurement samples in each experiment. When M is large enough, the measurement probability $p_X^{\mathrm{exp}}(\omega_j)$ is approximately normal distributed*

$$p_X^{\mathrm{exp}}(\omega_j) = p_X(\omega_j; \theta, \varphi, \chi) + \Sigma_{X,j} u_{X,j}, \text{ where } u_{X,j} \sim N(0,1) \text{ and } \frac{1-4(d\theta)^2}{4M} \leq \Sigma_{X,j}^2 \leq \frac{1}{4M}. \tag{47}$$

The same conclusion holds for $p_Y^{\mathrm{exp}}(\omega_j)$. Furthermore, by computing the Fourier coefficients via FFT, the Fourier coefficients are approximately complex normal distributed

$$c_k^{\mathrm{exp}} = c_k(\theta, \varphi, \chi) + \begin{cases} v_k & , k = 0, \cdots, d-1, \\ v_{2d-1+k} & , k = -d+1, \cdots, -1. \end{cases} \tag{48}$$

where v_k's are complex normal distributed random variables so that

$$\begin{aligned} &\text{for any } k: \ \mathbb{E}(v_k) = 0, \ \frac{1-2(d\theta)^2}{2M(2d-1)} \leq \mathbb{E}\left(|v_k|^2\right) \leq \frac{1}{2M(2d-1)}, \\ &\text{and for any } k \neq k': \ |\mathbb{E}(v_k \overline{v_{k'}})| \leq \frac{(d\theta)^2}{M(2d-1)}. \end{aligned} \tag{49}$$

Consequentially, when $d\theta \ll 1$, these random variables v_k's can be approximately assumed to be uncorrelated.

Proof. Given a quantum experiment with angle ω_j, the measurement generates i.i.d. Bernoulli distributed outcomes b_i's, namely $\mathbb{P}(b_i = 0) = 1 - \mathbb{P}(b_i = 1) = p_X(\omega_j; \theta, \varphi, \chi)$. Then, the measurement probability is estimated by $p_X^{\exp}(\omega_j) = \frac{1}{M}\sum_{i=1}^{M}(1 - b_i)$. When the sample size M is large enough, $p_X^{\exp}(\omega_j)$ is approximately normal distributed following the central limit theorem where the mean is $\mathbb{E}\left(p_X^{\exp}(\omega_j)\right) = p_X(\omega_j; \theta, \varphi, \chi)$ and the variance is

$$\begin{aligned}\Sigma_{X,j}^2 := \delta\left(p_X^{\exp}(\omega_j)\right) &= \frac{p_X(\omega_j;\theta,\varphi,\chi)\left(1 - p_X(\omega_j;\theta,\varphi,\chi)\right)}{M} \\ &= \frac{1}{4M} - \frac{\left(p_X(\omega_j;\theta,\varphi,\chi) - \frac{1}{2}\right)^2}{M} \leq \frac{1}{4M}.\end{aligned} \tag{50}$$

The other side of the inequality $\Sigma_{X,j}^2 \geq \frac{1-4(d\theta)^2}{4M}$ follows that $(p_X - \frac{1}{2})^2 \leq (d\theta)^2$ from Corollary 8. The same analysis is applicable to $p_Y^{\exp}(\omega_j)$.

To compute the Fourier coefficients from the experimental data, we perform FFT on $\mathfrak{h}_j^{\exp} := p_X^{\exp}(\omega_j) + \mathrm{i} p_Y^{\exp}(\omega_j) - \frac{1+\mathrm{i}}{2}$ reconstructed from the experimental data. We have $\mathbb{E}\left(\mathfrak{h}_j^{\exp}\right) = \mathfrak{h}(\omega_j;\theta,\varphi,\chi)$. Furthermore, let $\widetilde{u}_j = \mathfrak{h}_j^{\exp} - \mathbb{E}\left(\mathfrak{h}_j^{\exp}\right) = \Sigma_{X,j} u_{X,j} + \mathrm{i}\Sigma_{Y,j} u_{Y,j}$, then it holds that

$$\begin{aligned}&\text{for any } j,\ \mathbb{E}\left(\widetilde{u}_j\right) = 0,\ \mathbb{E}\left(\left|\widetilde{u}_j\right|^2\right) = \Sigma_{X,j}^2 + \Sigma_{Y,j}^2 = \frac{1}{2M} - \frac{\mathfrak{p}(\omega_j - \varphi, \theta)}{M}, \\ &\text{and for any } j \neq j', \mathbb{E}\left(\widetilde{u}_j \overline{\widetilde{u}_{j'}}\right) = 0.\end{aligned} \tag{51}$$

The FFT gives the Fourier coefficients as

$$\begin{pmatrix} c_0^{\exp} \\ \vdots \\ c_{d-1}^{\exp} \\ c_{-d+1}^{\exp} \\ \vdots \\ c_{-1}^{\exp} \end{pmatrix} = \frac{1}{2d-1}\Omega^\dagger \begin{pmatrix} \mathfrak{h}_0^{\exp} \\ \vdots \\ \mathfrak{h}_{2d-2}^{\exp} \end{pmatrix}, \text{ where } \Omega_{jk} = e^{\mathrm{i}\frac{2\pi jk}{2d-1}}. \tag{52}$$

Using the linearity, we get

$$v_k = \frac{1}{2d-1}\left(\Omega^\dagger \mathfrak{h}^{\exp}\right)_k = \frac{1}{2d-1}\sum_{j=0}^{2d-2} \overline{\Omega_{kj}} \widetilde{u}_j. \tag{53}$$

The mean is $\mathbb{E}\left(v_k\right) = \frac{1}{2d-1}\sum_{j=0}^{2d-2}\overline{\Omega_{kj}}\mathbb{E}\left(\widetilde{u}_j\right) = 0$. The covariance is

$$\mathbb{E}\left(v_k \overline{v_{k'}}\right) = \frac{1}{(2d-1)^2}\sum_{j,j'=0}^{2d-2}\overline{\Omega_{kj}}\Omega_{k'j'}\mathbb{E}\left(\widetilde{u}_j\overline{\widetilde{u}_{j'}}\right) = \frac{1}{(2d-1)^2}\sum_{j=0}^{2d-2} e^{\mathrm{i}\frac{2\pi}{2d-1}(k'-k)j}\mathbb{E}\left(\left|\widetilde{u}_j\right|^2\right). \tag{54}$$

When $k = k'$, it gives

$$\begin{aligned}&\mathbb{E}\left(\left|v_k\right|^2\right) = \frac{1}{2M(2d-1)} - \frac{1}{M(2d-1)^2}\sum_{j=0}^{2d-2}\mathfrak{p}(\omega_j - \varphi, \theta) \\ &\Rightarrow \frac{1 - 2(d\theta)^2}{2M(2d-1)} \leq \mathbb{E}\left(\left|v_k\right|^2\right) \leq \frac{1}{2M(2d-1)}\end{aligned} \tag{55}$$

where $0 \leq \mathfrak{p}(\omega_j - \varphi, \theta) \leq (d\theta)^2$ is used which follows Corollary 8.

On the other hand, when $k \neq k'$, the constant term $\frac{1}{2M}$ in $\mathbb{E}\left(\left|\widetilde{u}_j\right|^2\right)$ vanishes because $\sum_{j=0}^{2d-2} e^{\mathrm{i}\frac{2\pi}{2d-1}(k'-k)j} = (2d-1)\delta_{kk'}$. Then, using triangle inequality and Corollary 8, we get

$$\begin{aligned}\left|\mathbb{E}\left(v_k\overline{v_{k'}}\right)\right| &= \left|\frac{1}{(2d-1)^2}\sum_{j=0}^{2d-2} e^{\mathrm{i}\frac{2\pi}{2d-1}(k'-k)j}\mathfrak{p}(\omega_j - \varphi, \theta)\right| \\ &\leq \frac{1}{M(2d-1)^2}\sum_{j=0}^{2d-2}\left|\mathfrak{p}(\omega_j - \varphi, \theta)\right| \leq \frac{(d\theta)^2}{M(2d-1)}.\end{aligned} \tag{56}$$

The proof is completed. □

With a characterization of Monte Carlo sampling error, we are able to measure the robustness of the signal against error by the signal-to-noise ratio (SNR). The SNR of each Fourier coefficient is defined as the ratio between the squared Fourier coefficient and the variance of its associated additive sampling error. We define the SNR of QSPE in Problem 10 by the minimal component-wise SNR. The following theorem gives a characterization of the SNR.

Theorem 12. *When $d^{\frac{5}{4}}\theta \ll 1$, the signal-to-noise ratio satisfies*

$$\mathrm{SNR}_k := \frac{|c_k(\theta,\varphi,\chi)|^2}{\mathbb{E}\left(|v_k|^2\right)} \ge \mathrm{SNR} := 2(2d-1)M\sin^2\theta\left(1-\frac{4}{3}(d\theta)^2\left(1+3d^3\theta^2\right)\right). \tag{57}$$

Proof. According to Equation (31), for any $k = 0,\cdots,d-1$

$$1-\frac{2}{3}(d\theta)^2 \le \hat{c}_k^\star(\theta) \le 1. \tag{58}$$

Applying Theorem 9, we have

$$c_k(\theta,\varphi,\chi) \ge \sin\theta\left(\hat{c}_k^\star(\theta) - 2d^5\theta^4\right) \ge \sin\theta\left(1-\frac{2}{3}(d\theta)^2\left(1+3d^3\theta^2\right)\right). \tag{59}$$

Furthermore, by Bernoulli's inequality,

$$|c_k(\theta,\varphi,\chi)|^2 \ge \sin^2\theta\left(1-\frac{4}{3}(d\theta)^2\left(1+3d^3\theta^2\right)\right). \tag{60}$$

Combing the derived inequality with Lemma 11, it gives

$$\mathrm{SNR}_k = \frac{|c_k(\theta,\varphi,\chi)|^2}{\mathbb{E}\left(|v_k|^2\right)} \ge 2(2d-1)M\sin^2\theta\left(1-\frac{4}{3}(d\theta)^2\left(1+3d^3\theta^2\right)\right), \tag{61}$$

which completes the proof. □

B. Statistical estimator against Monte Carlo sampling error

As a consequence of Theorem 12, when SNR is high, namely $dM\theta^2 \gg 1$, the noise modeling in Ref. [20] suggests that a linear model with normal distributed noise can well approximate the problem in which the θ- and (φ,χ)-dependence are decoupled following Theorem 7. When $k = 0,\cdots,d-1$ and $d^5\theta^4 \ll 1$, we have

$$\begin{aligned}
&\mathsf{amplitude}\,(c_k^{\exp}) = \widetilde{c}_k(\theta) + v_k^{(\mathrm{amp})} \approx \theta + v_k^{(\mathrm{amp})},\\
&\mathsf{phase}(c_k^{\exp}) = \frac{\pi}{2} - \chi - (2k+1)\varphi + v_k^{(\mathrm{pha})} \text{ (up to } 2\pi\text{-periodicity)}
\end{aligned} \tag{62}$$

where $v_k^{(\mathrm{amp})}$ and $v_k^{(\mathrm{pha})}$ are normal distributed and are approximately $v_k^{(\mathrm{amp})} = \mathrm{Re}(v_k)$, $v_k^{(\mathrm{pha})} = \mathrm{Im}(v_k)/\widetilde{c}_k(\theta)$ according to Ref. [20]. Let the covariance matrices be $\mathcal{C}^{(\mathrm{amp})}$ and $\mathcal{C}^{(\mathrm{pha})}$. For any k and k'

$$\mathcal{C}_{k,k'}^{(\mathrm{amp})} := \mathbb{E}\left(v_k^{(\mathrm{amp})}v_{k'}^{(\mathrm{amp})}\right) \text{ and } \mathcal{C}_{k,k'}^{(\mathrm{pha})} := \mathbb{E}\left(v_k^{(\mathrm{pha})}v_{k'}^{(\mathrm{pha})}\right). \tag{63}$$

Let the data vectors be

$$|\mathbf{c}^{\exp}| := \left(\mathsf{amplitude}(c_0^{\exp}),\cdots,\mathsf{amplitude}(c_{d-1}^{\exp})\right)^\top,\ \widetilde{\mathbf{1}} = (\underbrace{1,\cdots,1}_{d})^\top. \tag{64}$$

The maximum likelihood estimator (MLE) is found by minimizing the negated log-likelihood function

$$\hat{\theta} = \underset{\theta}{\operatorname{argmin}}\left(|\mathbf{c}^{\exp}| - \theta\widetilde{\mathbf{1}}\right)^\top\left(\mathcal{C}^{(\mathrm{amp})}\right)^{-1}\left(|\mathbf{c}^{\exp}| - \theta\widetilde{\mathbf{1}}\right). \tag{65}$$

which follows the normality in Lemma 11.

In order to estimate φ, we can apply the Kay's phase unwrapping estimator in Ref.[10], a.k.a. weighted phase average estimator (WPA). The estimator is based on the sequential phase difference of the successive coefficients:

$$\mathsf{phase}\left(c_k^{\exp}\overline{c_{k+1}^{\exp}}\right) = 2\varphi + v_k^{(\mathrm{pha})} - v_{k+1}^{(\mathrm{pha})},\ k = 0, 1, \cdots, d-2. \tag{66}$$

Remarkably, by computing the sequential phase difference, the troublesome (2π)-periodicity in Equation (62) can be overcome. According to this equation, the noise is turned into a colored noise process. Let the covariance be

$$\mathcal{D}_{k,k'} := \mathbb{E}\left(\left(v_k^{(\mathrm{pha})} - v_{k+1}^{(\mathrm{pha})}\right)\left(v_{k'}^{(\mathrm{pha})} - v_{k'+1}^{(\mathrm{pha})}\right)\right) = \mathcal{C}_{k,k'}^{(\mathrm{pha})} + \mathcal{C}_{k+1,k'+1}^{(\mathrm{pha})} - \mathcal{C}_{k,k'+1}^{(\mathrm{pha})} - \mathcal{C}_{k+1,k'}^{(\mathrm{pha})}. \tag{67}$$

Then, the WPA estimator is derived by the following MLE:

$$\hat{\varphi} = \operatorname*{argmin}_{\varphi} \left(\boldsymbol{\Delta} - 2\varphi\mathbf{1}\right)^\top \mathcal{D}^{-1}\left(\boldsymbol{\Delta} - 2\varphi\mathbf{1}\right) \tag{68}$$

where the data vectors are

$$\boldsymbol{\Delta} = \left(\mathsf{phase}\left(c_0^{\exp}\overline{c_1^{\exp}}\right), \cdots, \mathsf{phase}\left(c_{d-2}^{\exp}\overline{c_{d-1}^{\exp}}\right)\right)^\top,\ \text{and } \mathbf{1} = (\underbrace{1, \cdots, 1}_{d-1})^\top. \tag{69}$$

To solve the MLE, we need to study the structure of covariance matrices, which is given by the following lemma.

Lemma 13. *When $d\theta \leq \frac{1}{5}$ and $d^3\theta^2 \leq 1$, for any $k \neq k'$*

$$\left|\mathcal{C}_{k,k'}^{(\mathrm{amp})}\right| \leq \frac{(d\sin\theta)^2}{M(2d-1)},\ \text{and } \left|\mathcal{C}_{k,k'}^{(\mathrm{pha})}\right| \leq \frac{4d^2}{3M(2d-1)}. \tag{70}$$

For any k

$$\left|\mathcal{C}_{k,k}^{(\mathrm{amp})} - \frac{1}{4M(2d-1)}\right| \leq \frac{2(d\sin\theta)^2}{M(2d-1)},\ \text{and } \left|\mathcal{C}_{k,k}^{(\mathrm{pha})} - \frac{1}{4M(2d-1)\sin^2\theta}\right| \leq \frac{10d^2}{3M(2d-1)}. \tag{71}$$

Proof. We first estimate the covariance of the real and imaginary components of the Monte Carlo sampling error in Fourier coefficients. Following Equation (53) and Lemma 11, for any $k \neq k'$,

$$\left|\mathbb{E}\left(\mathrm{Re}(v_k)\mathrm{Re}(v_{k'}) + \mathrm{Im}(v_k)\mathrm{Im}(v_{k'})\right)\right| \leq \left|\mathbb{E}\left(v_k\overline{v_{k'}}\right)\right| \leq \frac{(d\sin\theta)^2}{M(2d-1)}, \tag{72}$$

and for any k,

$$\frac{1-2(d\sin\theta)^2}{2M(2d-1)} \leq \mathbb{E}\left(\mathrm{Re}^2(v_k) + \mathrm{Im}^2(v_k)\right) = \mathbb{E}\left(|v_k|^2\right) \leq \frac{1}{2M(2d-1)}. \tag{73}$$

For any $k \neq k'$,

$$\begin{aligned}\left|\mathbb{E}\left(\mathrm{Re}(v_k)\mathrm{Re}(v_{k'}) - \mathrm{Im}(v_k)\mathrm{Im}(v_{k'})\right)\right| &\leq \left|\mathbb{E}\left(v_k v_{k'}\right)\right| = \left|\frac{1}{(2d-1)^2}\sum_{j,j'=0}^{2d-2}\overline{\Omega_{kj}\Omega_{k'j'}}\,\mathbb{E}\left(\widetilde{u}_j\widetilde{u}_{j'}\right)\right| \\ &\leq \frac{1}{(2d-1)^2}\sum_{j=0}^{2d-2}\left|\mathbb{E}\left(\widetilde{u}_j^2\right)\right| = \frac{1}{(2d-1)^2}\sum_{j=0}^{2d-2}\left|\Sigma_{X,j}^2 - \Sigma_{Y,j}^2\right| \\ &\leq \frac{1}{M(2d-1)^2}\sum_{j=0}^{2d-2}\mathfrak{p}(\omega_j - \varphi, \theta) \leq \frac{(d\sin\theta)^2}{M(2d-1)}.\end{aligned} \tag{74}$$

Similarly for any k,

$$\begin{aligned}\left|\mathbb{E}\left(\mathrm{Re}^2(v_k) - \mathrm{Im}^2(v_k)\right)\right| &\leq \left|\mathbb{E}\left(\mathrm{Re}^2(v_k) - \mathrm{Im}^2(v_k) + 2\mathrm{i}\mathrm{Re}(v_k)\mathrm{Im}(v_k)\right)\right| \\ &= \left|\mathbb{E}\left(v_k^2\right)\right| \leq \frac{1}{(2d-1)^2}\sum_{j=0}^{2d-2}\left|\mathbb{E}\left(\widetilde{u}_j^2\right)\right| \leq \frac{(d\sin\theta)^2}{M(2d-1)}.\end{aligned} \tag{75}$$

Using triangle inequality and the derived results, we have for any k

$$\begin{aligned}\left|\mathbb{E}\left(\mathrm{Re}^2(v_k)\right)-\frac{1}{4M(2d-1)}\right| \leq & \frac{1}{2}\left|\mathbb{E}\left(\mathrm{Re}^2(v_k)+\mathrm{Im}^2(v_k)\right)-\frac{1}{2M(2d-1)}\right| \\ & +\frac{1}{2}\left|\mathbb{E}\left(\mathrm{Re}^2(v_k)-\mathrm{Im}^2(v_k)\right)\right| \leq \frac{(d\sin\theta)^2}{M(2d-1)}.\end{aligned} \tag{76}$$

The same argument is applicable to the imaginary component

$$\left|\mathbb{E}\left(\mathrm{Im}^2(v_k)\right)-\frac{1}{4M(2d-1)}\right| \leq \frac{(d\sin\theta)^2}{M(2d-1)}. \tag{77}$$

When $k \neq k'$,

$$\begin{aligned}\left|\mathbb{E}\left(\mathrm{Re}(v_k)\mathrm{Re}(v_{k'})\right)\right| \leq & \frac{1}{2}\left|\mathbb{E}\left(\mathrm{Re}(v_k)\mathrm{Re}(v_{k'})+\mathrm{Im}(v_k)\mathrm{Im}(v_{k'})\right)\right| \\ & +\frac{1}{2}\left|\mathbb{E}\left(\mathrm{Re}(v_k)\mathrm{Re}(v_{k'})-\mathrm{Im}(v_k)\mathrm{Im}(v_{k'})\right)\right| \leq \frac{(d\sin\theta)^2}{M(2d-1)}\end{aligned} \tag{78}$$

and

$$\begin{aligned}\left|\mathbb{E}\left(\mathrm{Im}(v_k)\mathrm{Im}(v_{k'})\right)\right| \leq & \frac{1}{2}\left|\mathbb{E}\left(\mathrm{Re}(v_k)\mathrm{Re}(v_{k'})+\mathrm{Im}(v_k)\mathrm{Im}(v_{k'})\right)\right| \\ & +\frac{1}{2}\left|\mathbb{E}\left(\mathrm{Re}(v_k)\mathrm{Re}(v_{k'})-\mathrm{Im}(v_k)\mathrm{Im}(v_{k'})\right)\right| \leq \frac{(d\sin\theta)^2}{M(2d-1)}.\end{aligned} \tag{79}$$

Estimating Equation (31) gives for any $k \geq 0$

$$1-\frac{2}{3}(d\theta)^2 \leq \hat{c}_k^\star(\theta) \leq 1. \tag{80}$$

Assuming that $d^3\theta^2 \leq 1$, and applying Theorem 9, it holds that for any k

$$\sin\theta\left(1-\frac{8}{3}(d\theta)^2\right) \leq \widetilde{c}_k(\theta) \leq \sin\theta+2(d\theta)^5. \tag{81}$$

Furthermore, if $d\theta \leq \frac{1}{5}$, it holds that

$$\left|\frac{\sin\theta}{\widetilde{c}_k(\theta)}\right| \leq \frac{1}{1-\frac{8}{3}(d\theta)^2} < \sqrt{\frac{4}{3}}. \tag{82}$$

Then, for any $k \neq k'$

$$\left|\mathbb{E}\left(v_k^{(\mathrm{pha})}v_{k'}^{(\mathrm{pha})}\right)\right| = \frac{1}{|\widetilde{c}_k(\theta)\widetilde{c}_{k'}(\theta)|}\left|\mathbb{E}\left(\mathrm{Im}(v_k)\mathrm{Im}(v_{k'})\right)\right| \leq \frac{4d^2}{3M(2d-1)}. \tag{83}$$

For any k, applying triangle inequality, it yields that

$$\begin{aligned}&\left|\mathbb{E}\left(\left(v_k^{(\mathrm{pha})}\right)^2\right)-\frac{1}{4M(2d-1)\sin^2\theta}\right| \\ &\leq \frac{1}{\widetilde{c}_k^2(\theta)}\left|\mathbb{E}\left(\mathrm{Im}^2(v_k)\right)-\frac{1}{4M(2d-1)}\right|+\frac{1}{4M(2d-1)\sin^2\theta}\frac{\left|\widetilde{c}_k^2(\theta)-\sin^2\theta\right|}{\widetilde{c}_k^2(\theta)} \\ &\leq \frac{\sin^2\theta}{\widetilde{c}_k^2(\theta)}\frac{d^2}{M(2d-1)}\left(1+\frac{4}{3}\frac{\theta^2}{\sin^2\theta}\left(1+d^5\theta^4\right)\right) \leq \frac{5}{2}\frac{\sin^2\theta}{\widetilde{c}_k^2(\theta)}\frac{d^2}{M(2d-1)} \leq \frac{10d^2}{3M(2d-1)},\end{aligned} \tag{84}$$

where the inequality $\frac{\theta^2}{\sin^2\theta} \leq \frac{1}{25\sin^2(1/5)} < \frac{9}{8}$ when $\theta \leq \frac{1}{5d} \leq \frac{1}{5}$ is used to simplify the constant. The proof is completed. □

Because of the sequential phase difference, we also need to study the structure of the covariance matrix of the colored noise in Equation (66). It is given by the following corollary.

Corollary 14. *Let*

$$\widetilde{D} := \frac{1}{4M(2d-1)\sin^2\theta}\mathfrak{D}, \text{ where } \mathfrak{D}_{k,k'} = \begin{cases} 2 & , \ k = k', \\ -1 & , \ |k-k'| = 1, \\ 0 & , \ \text{otherwise}. \end{cases} \tag{85}$$

Then, when $d\theta \leq \frac{1}{5}$ *and* $d^3\theta^2 \leq 1$,

$$\left| D_{k,k'} - \widetilde{D}_{k,k'} \right| \leq \frac{d^2}{M(2d-1)} \times \begin{cases} \frac{28}{3} & , k = k', \\ \frac{22}{3} & , |k-k'| = 1, \\ \frac{16}{3} & , \text{otherwise}. \end{cases} \tag{86}$$

Proof. The element-wise bound Equation (86) follows immediately by applying triangle inequality with Lemma 13 and the defining equation Equation (67). □

Consequentially, the log-likelihood functions are well approximated by quadratic forms in terms constant matrices. The approximate forms yield the maximum likelihood estimators of QSPE.

Definition 15 (QSPE estimators)**.** *For any* $k = 0, \ldots, d-2$, *the sequential phase difference is defined as*

$$\begin{aligned} \Delta_k &:= \mathsf{phase}\left(c_k^{\exp}\overline{c_{k+1}^{\exp}}\right), \\ \text{and} \quad \mathbf{\Delta} &:= (\Delta_0, \Delta_1, \ldots, \Delta_{d-2})^\top. \end{aligned} \tag{87}$$

Let the all-one vector be $\mathbf{1} = (\underbrace{1, \ldots, 1}_{d-1})^\top$ *and the discrete Laplacian matrix be*

$$\mathfrak{D} = \begin{pmatrix} 2 & -1 & 0 & \cdots & 0 \\ -1 & 2 & -1 & \cdots & 0 \\ 0 & -1 & 2 & \cdots & 0 \\ \vdots & \vdots & \vdots & & \vdots \\ 0 & 0 & 0 & \cdots & 2 \end{pmatrix} \in \mathbb{R}^{(d-1)\times(d-1)}.$$

The statistical estimators solving QSPE are

$$\hat{\theta} = \frac{1}{d}\sum_{k=0}^{d-1} |c_k^{\exp}| \quad \text{and} \quad \hat{\varphi} = \frac{1}{2}\frac{\mathbf{1}^\top \mathfrak{D}^{-1}\mathbf{\Delta}}{\mathbf{1}^\top \mathfrak{D}^{-1}\mathbf{1}}. \tag{88}$$

Their variances can also be computed by using approximate covariance matrices, which gives

$$\mathrm{Var}\left(\hat{\theta}\right) \approx \frac{1}{4Md(2d-1)} \approx \frac{1}{8Md^2} \quad \text{and} \quad \mathrm{Var}\left(\hat{\varphi}\right) \approx \frac{3}{4Md(2d-1)(d^2-1)\theta^2} \approx \frac{3}{8Md^4\theta^2}. \tag{89}$$

In practice, an additional moving average filter in Ref. [19] can be applied to the data to further numerically boost the SNR. For completeness, we exactly compute the optimal variance from Cramér-Rao lower bound and discuss the optimality of the estimators in Section Supplementary Information 6.

C. Improving the estimator of swap angle using the peak information provided by $\hat{\varphi}$

In this subsection, we explicitly write down the dependence on d as the subscript of relevant functions because d is variable in the analysis.

Once we have a priori $\hat{\varphi}_{\mathrm{pri}}$, it gives an accurate estimation of phase making $|\mathfrak{h}|$ attain its maximum, which is often referred to as the phase matching condition. The a priori phase $\hat{\varphi}_{\mathrm{pri}}$ can be some statistical estimator from other subroutines. For example, it can be the QSPE φ-estimator. By setting the phase modulation angle to $\omega = \hat{\varphi}_{\mathrm{pri}}$ in the QSPE circuit, we compute the amplitude of the reconstructed function for variable degrees and compute the differential signal by

$$\begin{aligned} &\{|\mathfrak{h}_j^{\exp}| : j = d, d+2, d+4, \cdots, 3d\} \\ &\Rightarrow \mathbf{\Gamma} := \left(|\mathfrak{h}_{d+2}^{\exp}| - |\mathfrak{h}_d^{\exp}|, |\mathfrak{h}_{d+4}^{\exp}| - |\mathfrak{h}_{d+2}^{\exp}|, \cdots, |\mathfrak{h}_{3d}^{\exp}| - |\mathfrak{h}_{3d-2}^{\exp}|\right)^\top \in \mathbb{R}^d. \end{aligned} \tag{90}$$

Let $\mathfrak{D}$ be the d-by-d discrete Laplacian matrix and $\mathbf{1} := (1, 1, \cdots, 1) \in \mathbb{R}^d$. The swap angle can be estimated by the statistical estimator

$$\hat{\theta}_{\mathrm{pd}} = \frac{1}{2} \frac{\mathbf{1}^\top \mathfrak{D}^{-1} \mathbf{\Gamma}}{\mathbf{1}^\top \mathfrak{D}^{-1} \mathbf{1}}. \tag{91}$$

The performance guarantee of this estimator is given in the following theorem. We also discuss the case that the a priori is given by the QSPE estimator in the next corollary.

Theorem 16. *Assume an unbiased estimator $\hat{\varphi}_{\mathrm{pri}}$ with variance $\mathrm{Var}(\hat{\varphi}_{\mathrm{pri}})$ is used as a priori. When $d\theta \leq \frac{1}{9}$, the estimator $\hat{\theta}_{\mathrm{pd}}$ is a biased estimator with bounded bias*

$$|\mathrm{Bias}_{\mathrm{pd}}| := \left| \mathbb{E}\left(\hat{\theta}_{\mathrm{pd}}\right) - \theta \right| \leq \frac{13}{2} d^2 \theta \mathrm{Var}(\hat{\varphi}) + 37 (d\theta)^3 \tag{92}$$

and variance

$$\mathrm{Var}\left(\hat{\theta}_{\mathrm{pd}}\right) = \frac{3}{4Md(d+1)(d+2)} \approx \frac{3}{4d^3 M}. \tag{93}$$

Proof. Let the amplitude of the reconstructed function be

$$\mathfrak{f}_d(\omega - \varphi, \theta) := |\mathfrak{h}_d(\omega; \theta, \varphi, \chi)| = \sin\theta \left| \frac{\sin(d\sigma)}{\sin\sigma} \right| \sqrt{1 - \sin^2(\theta) \frac{\sin^2(d\sigma)}{\sin^2(\sigma)}}, \tag{94}$$

which follows Corollary 8 and $\sigma := \arccos(\cos(\theta)\cos(\omega - \varphi))$. Furthermore, let

$$\widetilde{\mathfrak{f}}^\circ_d(\omega - \varphi, \theta) := \sin\theta \frac{\sin(d(\omega - \varphi))}{\sin(\omega - \varphi)}, \quad \mathfrak{f}^\circ_d(\omega - \varphi, \theta) := \left| \widetilde{\mathfrak{f}}^\circ_d(\omega - \varphi, \theta) \right|. \tag{95}$$

Note that when $|\omega - \varphi| \leq \frac{\pi}{d}$, the defined function agrees with the amplitude of itself $\mathfrak{f}^\circ_d(\omega - \varphi, \theta) = \widetilde{\mathfrak{f}}^\circ_d(\omega - \varphi, \theta)$. Furthermore, for any ω, we have the following bound by using triangle inequality

$$\begin{aligned} |\mathfrak{f}^\circ_d(\omega - \varphi, \theta) - \mathfrak{f}_d(\omega - \varphi, \theta)| &\leq \left| \sin\theta \frac{\sin(d\sigma)}{\sin\sigma} \sqrt{1 - \sin^2(\theta) \frac{\sin^2(d\sigma)}{\sin^2(\sigma)}} - \sin\theta \frac{\sin(d(\omega - \varphi))}{\sin(\omega - \varphi)} \right| \\ &\leq \sin\theta \left| \frac{\sin(d\sigma)}{\sin\sigma} \right| \left(1 - \sqrt{1 - \sin^2(\theta) \frac{\sin^2(d\sigma)}{\sin^2(\sigma)}} \right) + \sin\theta \left| \frac{\sin(d\sigma)}{\sin\sigma} - \frac{\sin(d(\omega - \varphi))}{\sin(\omega - \varphi)} \right| \\ &:= J_1(d) + J_2(d). \end{aligned} \tag{96}$$

The first term can be further upper bounded by using the fact that $\max_x \left| \frac{\sin(dx)}{\sin x} \right| = d$

$$J_1(d) = \frac{\sin^3\theta \left| \frac{\sin(d\sigma)}{\sin\sigma} \right|^3}{1 + \sqrt{1 - \sin^2(\theta) \frac{\sin^2(d\sigma)}{\sin^2(\sigma)}}} \leq \frac{(d\theta)^3}{1 + \sqrt{1 - (d\theta)^2}} \leq \frac{(d\theta)^3}{1 + 2\sqrt{2}/3} \tag{97}$$

where the last inequality uses the condition $3d\theta \leq \frac{1}{3}$. The last inequality is established so that it holds for any $J_1(d), \cdots, J_1(3d)$. Note that the Chebyshev polynomial of the second kind is $U_{d-1}(\cos\sigma) = \frac{\sin(d\sigma)}{\sin\sigma}$ and it is related to the derivative of the Chebyshev polynomial of the first kind as $U_{d-1} = \frac{1}{d-1} T'_{d-1}$. Using the intermediate value theorem, there exists ξ in between $\cos\theta\cos(\omega - \varphi)$ and $\cos(\omega - \varphi)$ so that

$$\begin{aligned} J_2(d) &= \sin\theta |U_{d-1}(\cos\theta\cos(\omega - \varphi)) - U_{d-1}(\cos(\omega - \varphi))| \\ &= \sin\theta \left| U'_{d-1}(\xi) \right| |\cos(\omega - \varphi)| (1 - \cos\theta) \leq \frac{\theta^3}{2(d-1)} \max_{-1 \leq x \leq 1} \left| T''_{d-1}(x) \right| \\ &\leq \frac{\theta^3}{2(d-1)} \frac{(d-1)^2 \left((d-1)^2 - 1 \right)}{3} \max_{-1 \leq x \leq 1} |T_{d-1}(x)| = \frac{d(d-1)(d-2)\theta^3}{6} \leq \frac{(d\theta)^3}{6}. \end{aligned} \tag{98}$$

Here, the Markov brothers' inequality (Theorem 2) is invoked to bound the second order derivative. Thus, the approximation error is

$$\max_{\omega\in[0,\pi]} |\mathfrak{f}^{\circ}_d(\omega-\varphi,\theta)-\mathfrak{f}_d(\omega-\varphi,\theta)| \leq C(d\theta)^3 \quad \text{where } C=\frac{1}{1+2\sqrt{2}/3}+\frac{1}{6}\approx 0.6814. \tag{99}$$

When $|\omega-\varphi|\leq \frac{\pi}{d}$, the absolute value can be discarded and we can consider $\widetilde{\mathfrak{f}}^{\circ}_d$ instead. Taking the difference of the function, it yields

$$\widetilde{\mathfrak{f}}^{\circ}_{d+2}(\omega-\varphi,\theta)-\widetilde{\mathfrak{f}}^{\circ}_d(\omega-\varphi,\theta)=2\sin\theta\cos\left((d+1)(\omega-\varphi)\right). \tag{100}$$

Let the differential signal be

$$\Gamma_d(\omega-\varphi,\theta):=\mathfrak{f}_{d+2}(\omega-\varphi,\theta)-\mathfrak{f}_d(\omega-\varphi,\theta)=2\theta+\delta_d(\omega-\varphi,\theta) \tag{101}$$

where $\delta_d(\omega-\varphi,\theta)$ is the systematic error raising in the linearization of the model. Using Equations (99) and (100), when $|\omega-\varphi|\leq\frac{\pi}{d+2}$, the systematic error is bounded as

$$\begin{aligned}|\delta_d(\omega-\varphi,\theta)| &\leq \left|\widetilde{\mathfrak{f}}^{\circ}_{d+2}(\omega-\varphi,\theta)-\widetilde{\mathfrak{f}}^{\circ}_d(\omega-\varphi,\theta)-2\theta\right|+C\theta^3\left(d^3+(d+2)^3\right)\\ &\leq 2|\cos\left((d+1)(\omega-\varphi)\right)|\left(\theta-\sin\theta\right)+2\theta\left(1-\cos\left((d+1)(\omega-\varphi)\right)\right)+C\theta^3\left(d^3+(d+2)^3\right)\\ &\leq \theta\left(d+1\right)^2\left(\omega-\varphi\right)^2+C\theta^3\left(d^3+(d+2)^3\right)+2\theta^3.\end{aligned} \tag{102}$$

Furthermore, the differential signal is also bounded

$$\begin{aligned}|\Gamma_d(\omega-\varphi,\theta)| &\leq \left|\widetilde{\mathfrak{f}}^{\circ}_{d+2}(\omega-\varphi,\theta)-\widetilde{\mathfrak{f}}^{\circ}_d(\omega-\varphi,\theta)\right|+C\theta^3\left(d^3+(d+2)^3\right)\\ &=2\sin\theta|\cos\left((d+1)(\omega-\varphi)\right)|+C\theta^3\left(d^3+(d+2)^3\right)\leq 2\theta+C\theta^3\left(d^3+(d+2)^3\right).\end{aligned} \tag{103}$$

In the experimental implementation, we perform the QSPE circuit with $\omega=\hat{\varphi}_{\mathrm{pri}}$ and degree $d, d+2, d+4,\cdots,3d$. The resulted dataset contains $\left\{\mathfrak{f}^{\exp}_j:=\left|\mathfrak{h}^{\exp}_j\right|: j=d,d+2,\cdots,3d\right\}$ and the differential signal can be computed respectively

$$\Gamma^{\exp}_j:=\mathfrak{f}^{\exp}_{j+2}-\mathfrak{f}^{\exp}_j=\Gamma_j(\hat{\varphi}-\varphi,\theta)+w_{j+2}-w_j=2\theta+\delta_j(\hat{\varphi}-\varphi,\theta)+w_{j+2}-w_j \tag{104}$$

where $w_j:=\mathfrak{f}^{\exp}_j-\mathfrak{f}_j(\hat{\varphi}-\varphi)$ is the noise of the sampled data. When the SNR is large, Ref. [20] suggests the noise can be approximated by the real component of the noise on the complex-valued data $\mathfrak{h}^{\exp}_j$. Analyzed in the proof of Lemma 11, the variance of the noise concentrates around a constant

$$\mathbb{E}\left(w_j\right)=0 \quad \text{and} \quad \frac{1}{4M}-\frac{(j\theta)^2}{M}\leq \mathrm{Var}(w_j)\leq\frac{1}{4M}. \tag{105}$$

Assume $3d\theta\ll 1$, the covariance matrix of the colored noise $w_{j+2}-w_j$ is well approximated by a constant matrix

$$\mathbb{E}\left(\left(w_{d+2(j+1)}-w_{d+2j}\right)\left(w_{d+2(k+1)}-w_{d+2k}\right)\right)\approx\frac{1}{4M}\mathfrak{D}_{j,k}. \tag{106}$$

Let the data vector be

$$\mathbf{\Gamma}=\left(\Gamma^{\exp}_d,\Gamma^{\exp}_{d+2},\cdots,\Gamma^{\exp}_{3d-2}\right)^{\top}\in\mathbb{R}^d \tag{107}$$

and the systematic error vector be

$$\boldsymbol{\delta}(\hat{\varphi}-\varphi,\theta)=\left(\delta_d(\hat{\varphi}-\varphi,\theta),\delta_{d+2}(\hat{\varphi}-\varphi,\theta),\cdots,\delta_{3d-2}(\hat{\varphi}-\varphi,\theta)\right)^{\top}\in\mathbb{R}^d. \tag{108}$$

The statistical estimator solving the linearized problem of Equation (104) is

$$\hat{\theta}_{\mathrm{pd}}=\frac{1}{2}\frac{\mathbf{1}^{\top}\mathfrak{D}^{-1}\mathbf{\Gamma}}{\mathbf{1}^{\top}\mathfrak{D}^{-1}\mathbf{1}}. \tag{109}$$

According to Ref. [10], the matrix-multiplication form can be exactly represented as a convex combination: for any d-dimensional vector $\mathbf{X}=(X_0,\cdots,X_{d-1})^{\top}$

$$\frac{\mathbf{1}^{\top}\mathfrak{D}^{-1}\mathbf{X}}{\mathbf{1}^{\top}\mathfrak{D}^{-1}\mathbf{1}}=\sum_{k=0}^{d-1}\mu_k X_k \tag{110}$$

where

$$\mu_k := \frac{\frac{3}{2}(d+1)}{(d+1)^2-1}\left(1-\left(\frac{k-\frac{d-1}{2}}{\frac{d+1}{2}}\right)^2\right) > 0 \text{ and } \sum_{k=0}^{d-1}\mu_k = 1. \tag{111}$$

The variance of the estimator is

$$\mathrm{Var}\left(\hat{\theta}_{\mathrm{pd}}\right) = \frac{1}{4}\frac{1}{4M}\frac{1}{\mathbf{1}^\top\mathfrak{D}^{-1}\mathbf{1}} = \frac{3}{4Md(d+1)(d+2)} \approx \frac{3}{4d^3M}. \tag{112}$$

The conditional mean of the estimator is bounded as

$$\left|\mathbb{E}\left(\hat{\theta}_{\mathrm{pd}}\middle|\hat{\varphi}_{\mathrm{pri}}\right)\right| = \left|\frac{1}{2}\sum_{k=0}^{d-1}\mu_k\Gamma_{d+2k}(\hat{\varphi}_{\mathrm{pri}}-\varphi,\theta)\right| \leq \frac{1}{2}\max_{k=0,\cdots,d-1}\left|\Gamma_{d+2k}(\hat{\varphi}_{\mathrm{pri}}-\varphi,\theta)\right| \leq \theta + C(3d\theta)^3. \tag{113}$$

To make the bound in Equation (102) justified, we first assume that $|\hat{\varphi}_{\mathrm{pri}}-\varphi| \leq \frac{\pi}{3d}$. Invoking Chebyshev's inequality, the assumption fails with probability

$$\mathbb{P}\left(|\hat{\varphi}_{\mathrm{pri}}-\varphi| > \frac{\pi}{3d}\right) \leq \frac{9d^2}{\pi^2}\mathrm{Var}\left(\hat{\varphi}_{\mathrm{pri}}\right) \leq d^2\mathrm{Var}\left(\hat{\varphi}_{\mathrm{pri}}\right). \tag{114}$$

When $|\hat{\varphi}_{\mathrm{pri}}-\varphi| \leq \frac{\pi}{3d}$, the conditional expectation of the estimator is

$$\mathbb{E}\left(\hat{\theta}_{\mathrm{pd}}\mathbb{I}_{|\hat{\varphi}_{\mathrm{pri}}-\varphi|\leq\frac{\pi}{3d}}\middle|\hat{\varphi}_{\mathrm{pri}}\right) = \left(\theta + \frac{1}{2}\frac{\mathbf{1}^\top\mathfrak{D}^{-1}\boldsymbol{\delta}(\hat{\varphi}_{\mathrm{pri}}-\varphi,\theta)}{\mathbf{1}^\top\mathfrak{D}^{-1}\mathbf{1}}\right)\mathbb{I}_{|\hat{\varphi}_{\mathrm{pri}}-\varphi|\leq\frac{\pi}{3d}}. \tag{115}$$

Invoking Equation (102), when $|\hat{\varphi}_{\mathrm{pri}}-\varphi| \leq \frac{\pi}{3d}$, the bias of the estimator is bounded as

$$\begin{aligned}
&\left|\mathbb{E}\left(\left(\hat{\theta}_{\mathrm{pd}}-\theta\right)\mathbb{I}_{|\hat{\varphi}_{\mathrm{pri}}-\varphi|\leq\frac{\pi}{3d}}\right)\right| = \left|\mathbb{E}\left(\mathbb{E}\left(\left(\hat{\theta}_{\mathrm{pd}}-\theta\right)\mathbb{I}_{|\hat{\varphi}_{\mathrm{pri}}-\varphi|\leq\frac{\pi}{3d}}\middle|\hat{\varphi}_{\mathrm{pri}}\right)\right)\right| \\
&= \frac{1}{2}\left|\sum_{k=0}^{d-1}\mu_k\mathbb{E}\left(\delta_{d+2k}(\hat{\varphi}_{\mathrm{pri}}-\varphi,\theta)\mathbb{I}_{|\hat{\varphi}_{\mathrm{pri}}-\varphi|\leq\frac{\pi}{3d}}\right)\right| \leq \frac{1}{2}\max_{k=0,\cdots,d-1}\mathbb{E}\left(|\delta_{d+2k}(\hat{\varphi}_{\mathrm{pri}}-\varphi,\theta)|\right) \\
&\leq \frac{1}{2}\theta\left(3d-1\right)^2\mathrm{Var}\left(\hat{\varphi}_{\mathrm{pri}}\right) + C\theta^3\left(\frac{1}{C}+\frac{(3d-2)^3+(3d)^3}{2}\right) \leq \frac{1}{2}\theta(3d)^2\mathrm{Var}\left(\hat{\varphi}_{\mathrm{pri}}\right) + C(3d\theta)^3.
\end{aligned} \tag{116}$$

On the other hand, when $|\hat{\varphi}_{\mathrm{pri}}-\varphi| > \frac{\pi}{3d}$, the bias of the estimator is bounded as

$$\begin{aligned}
&\left|\mathbb{E}\left(\left(\hat{\theta}_{\mathrm{pd}}-\theta\right)\mathbb{I}_{|\hat{\varphi}_{\mathrm{pri}}-\varphi|>\frac{\pi}{3d}}\right)\right| = \left|\mathbb{E}\left(\left(\mathbb{E}\left(\hat{\theta}_{\mathrm{pd}}\middle|\hat{\varphi}_{\mathrm{pri}}\right)-\theta\right)\mathbb{I}_{|\hat{\varphi}_{\mathrm{pri}}-\varphi|>\frac{\pi}{3d}}\right)\right| \\
&\leq \left(2\theta + C(3d\theta)^3\right)\mathbb{P}\left(|\hat{\varphi}_{\mathrm{pri}}-\varphi| > \frac{\pi}{3d}\right) \leq 2d^2\theta\mathrm{Var}\left(\hat{\varphi}_{\mathrm{pri}}\right) + C(3d\theta)^3.
\end{aligned} \tag{117}$$

Combining these two cases and using triangle inequality, the bias is bounded as

$$\begin{aligned}
|\mathrm{Bias}_{\mathrm{pd}}| &\leq \left|\mathbb{E}\left(\left(\hat{\theta}_{\mathrm{pd}}-\theta\right)\mathbb{I}_{|\hat{\varphi}_{\mathrm{pri}}-\varphi|\leq\frac{\pi}{3d}}\right)\right| + \left|\mathbb{E}\left(\left(\hat{\theta}_{\mathrm{pd}}-\theta\right)\mathbb{I}_{|\hat{\varphi}_{\mathrm{pri}}-\varphi|>\frac{\pi}{3d}}\right)\right| \\
&\leq \frac{13}{2}d^2\theta\mathrm{Var}\left(\hat{\varphi}_{\mathrm{pri}}\right) + 37(d\theta)^3.
\end{aligned} \tag{118}$$

Here, we use $54C \leq 37$ to simplify the preconstant. The proof is completed. □

Corollary 17. *When $\hat{\varphi}_{\mathrm{pri}} = \hat{\varphi}$ is the QSPE φ-estimator in Definition 15, the bias of the estimator is bounded as*

$$|\mathrm{Bias}_{\mathrm{pd}}| \leq \frac{39}{16d^2M\theta} + \frac{7d\theta}{M} + 19\left(d\theta\right)^3. \tag{119}$$

Proof. The upper bound follows the substitution $\mathrm{Var}\left(\hat{\varphi}\right) \approx \frac{3}{8d^4\theta^2M}$. Furthermore, the second term comes from the refinement in the upper bound in Equation (117)

$$C(3d\theta)^3\mathbb{P}\left(|\hat{\varphi}-\varphi| > \frac{\pi}{3d}\right) \leq C(3d\theta)^3d^2\mathrm{Var}\left(\hat{\varphi}\right) \leq \frac{81Cd\theta}{8M} \leq \frac{7d\theta}{M}. \tag{120}$$

□

The analysis in this section indicates that trusting the a priori phase as the "peak" location and estimating θ from the differential signal at the "peak" will unavoidably introduce bias to the θ-estimator. Unless the a priori is deterministic and is exactly equal to φ, the "peak" is not the exact peak even subjected to the controllable statistical fluctuation of $\hat{\varphi}_{\mathrm{pri}}$. Hence, it suggests that we need to interpret the a priori $\hat{\varphi}_{\mathrm{pri}}$ as an estimated peak location which is close to the exact peak location φ. This gives rise to the regression-based methods in the next subsection.

D. Peak regression and peak fitting

In order to circumvent the over-confident reliance on the a priori guess of φ, the method can be improved by regressing distinct samples with respect to analytical expressions on the unknown angle parameters. Suppose n samples are made with $\{(\omega_j, d_j, \mathfrak{h}^{\exp,j}) : j = 1, \cdots, n\}$. One can consider performing a nonlinear regression on the data to infer the unknown parameters, which is given by the following minimization problem

$$\hat{\theta}_{\mathrm{pr}}, \hat{\varphi}_{\mathrm{pr}}, \hat{\chi}_{\mathrm{pr}} = \underset{\theta,\varphi,\chi}{\operatorname{argmin}} \sum_{j=1}^{n} \left| \mathfrak{h}_{d_j}(\omega_j; \theta, \varphi, \chi) - \mathfrak{h}^{\exp,j} \right|^2. \tag{121}$$

When the number of additional samples n is large enough, the estimator derived from the minimization problem is expected to be unbiased and the variance scales as $\mathcal{O}(1/(d^2 nM))$ according to the M-estimation theory [11]. However, the practical implementation of these estimators is easily affected by the complex landscape of the minimization problem. Meanwhile, the sub-optimality and the run time of black-box optimization algorithms also limit the use of these estimators.

To overcome the difficulty due to the complex landscape of nonlinear regression, we propose another technique to improve the accuracy of the swap-angle estimator by fitting the peak of the amplitude function $\mathfrak{f}_d(\omega - \varphi, \theta)$. We observe that the amplitude function is well captured by a parabola on the interval $\mathcal{I} := \left[\varphi - \frac{\pi}{2d}, \varphi + \frac{\pi}{2d}\right]$. Consider n_{pf} equally spaced sample points on the interval $\mathcal{I}$: $\omega_j^{(\mathrm{pf})} = \hat{\varphi}_{\mathrm{pri}} + \frac{\pi}{d}\left(\frac{j}{n_{\mathrm{pf}}-1} - \frac{1}{2}\right)$ where $j = 0, 1, \cdots, n_{\mathrm{pf}} - 1$. We find the best parabola fitting the sampled data $\mathfrak{f}_d^{\exp}\left(\omega_j^{(\mathrm{pf})}\right)$ whose maximum $\mathfrak{f}_d^{(\mathrm{pf\ max})}$ attains at $\omega^{(\mathrm{pf\ max})}$. Given that $\hat{\varphi}_{\mathrm{pri}}$ is an accurate estimator of the angle φ, we accept the parabolic fitting result if the peak location does not deviate $\hat{\varphi}_{\mathrm{pri}}$ beyond some threshold $\varepsilon^{\mathrm{thr}}$, namely, the fitting is accepted if $\left|\omega^{(\mathrm{pf\ max})} - \hat{\varphi}_{\mathrm{pri}}\right| < \varepsilon^{\mathrm{thr}}$. Upon the acceptance, the estimator is $\hat{\theta}_{\mathrm{pf}} := \mathfrak{f}_d^{(\mathrm{pf\ max})}/d$. Ignoring the systematic bias caused by the overshooting of $\hat{\varphi} \neq \varphi$, the variance of the estimator is approximately $\mathcal{O}\left(\frac{1}{d^2 n_{\mathrm{pf}}}\right)$. The detailed procedure is given in Algorithm 1.

Algorithm1 Improving θ estimation using peak fitting

Input: A U-gate $U(\theta, \varphi, \chi, *)$, an integer d (the number of applications of U-gate), an integer n (the number of sampled angles), a priori $\hat{\varphi}_{\mathrm{pri}}$ (can be generated by QSPE), a threshold $\beta^{\mathrm{thr}} \in [0, 1]$.
Output: Estimators $\hat{\theta}_{\mathrm{pf}}$

Initiate real-valued data vectors $\mathfrak{p}^{\exp}, \mathfrak{w} \in \mathbb{R}^n$.
for $j = 0, 1, \cdots, n-1$ **do**
  Set the tunable Z-phase modulation angle as $\omega_j = \hat{\varphi}_{\mathrm{pri}} + \frac{\pi}{d}\left(\frac{j}{n-1} - \frac{1}{2}\right)$.
  Peform the quantum circuit in Figure 1 in the main text (or Figure 3) and measure the transition probabilities $p_X^{\exp}(\omega_j)$ and $p_Y^{\exp}(\omega_j)$.
  Set $\mathfrak{p}_j^{\exp} \leftarrow \sqrt{\left(p_X^{\exp}(\omega_j) - \frac{1}{2}\right)^2 + \left(p_Y^{\exp}(\omega_j) - \frac{1}{2}\right)^2}$ and $\mathfrak{w}_j \leftarrow \omega_j$.
end for
Fit $(\mathfrak{w}, \mathfrak{p}^{\exp})$ with respect to to parabolic model $\mathfrak{p} = \beta_0 (\mathfrak{w} - \beta_1)^2 + \beta_2$.
if $\beta_0 < 0$ (concavity) and $|\beta_1 - \hat{\varphi}_{\mathrm{pri}}| < \beta^{\mathrm{thr}}$ (small deviation from a priori) **then**
  Set $\hat{\theta}_{\mathrm{pf}} \leftarrow \beta_2/d$. The improvement is accepted.
else
  Set $\hat{\theta}_{\mathrm{pf}} \leftarrow$ None. The improvement is rejected.
end if

E. Numerical performance of QSPE on FsimGate against Monte Carlo sampling error

To numerically test the performance of QSPE and validate the analysis in the presence of Monte Carlo sampling error, we simulate the quantum circuit and perform the inference. In Figure 5, we plot the squared error of each estimator as a function of the number of FsimGates d in each quantum circuit. Consequentially, each data point is the mean squared error (MSE), which is a metric of the performance according to the bias-variance decomposition $\mathrm{MSE} = \mathrm{Var} + \mathrm{bias}^2$. The numerical results in Figure 5 indicate that although $\theta = 1 \times 10^{-3}$ is small, QSPE estimators achieve an accurate estimation with a very small d. The numerical results also show that the performance of the estimator does not significantly depend on the value of the single-qubit phase φ. Meanwhile, using the peak fitting in Algorithm 1, the variance in θ-estimation is improved so that the MSE curve is lowered. Zooming the MSE curve in log-log scale, the curve scales as a function of d as the theoretically derived variance scaling in Theorem 2 in the main text. We will discuss the scaling of the variance in Section Supplementary Information 6 in more detail.

In Figure 6, we perform the numerical simulation with variable swap angle θ and the number of measurement samples M. The numerical results show that the accuracy of φ-estimation is more vulnerable to decreasing θ. This is explainable from the theoretically derived variance in Theorem 2 in the main text which depends on the swap angle as $1/\theta^2$. Although the theoretical variance of θ is expected to be invariant for different θ values, the numerical results show that the MSE of θ-estimation gets larger when smaller θ is used, and the scaling of the curve differs from the classical scaling $1/M$. The reason is that when $\theta \leq 5 \times 10^{-4}$, the SNR is not large enough so that the theoretical derivation can be justified. When using a bigger d or M, the curve will converge to the theoretical derivation. When $\theta = 1 \times 10^{-3}$, the setting of the experiments is enough to get a large enough SNR. Hence, the scaling of the MSE curves in the bottom panels in Figure 6 agrees with the classical scaling $1/M$ of Monte Carlo sampling error.

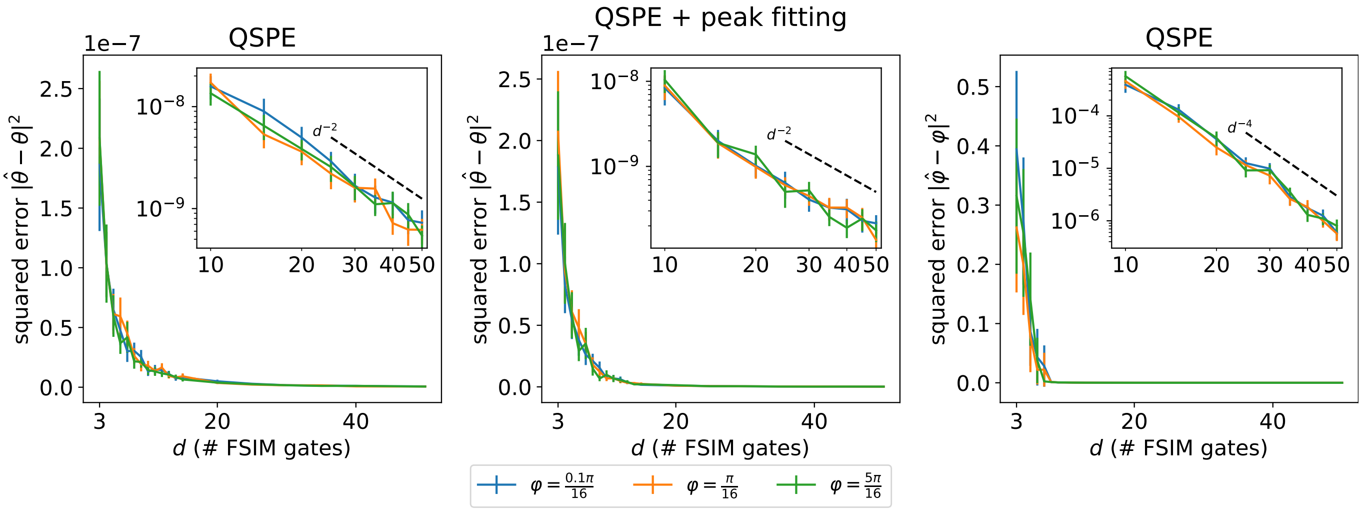

Supplementary Figure 5. Squared error of estimators as a function of the number of FsimGate's. The only source of noise in the numerical experiments is the Monte Carlo sampling error. The number of measurement samples is $M = 1 \times 10^5$, and $n_{\mathrm{pf}} = 15$ is used in the peak fitting. The swap angle is set to $\theta = 1 \times 10^{-3}$ and the phase parameter is set to $\chi = 5\pi/32$. The error bar of each point stands for the confidence interval derived from 96 independent repetitions.

Supplementary Information 5. Solving QSPE with arbitrary swap-angle value

In the early section, we derive robust statistical estimators when $d\theta \leq 1$. These estimators are based on linear statistical models which rely on the approximation of Fourier coefficients in the desired regime. In this section, we aim to generalize the solution to arbitrary swap-angle value which expands the use of QSPE to generic U-gates.

Recall that in Theorem 7, we show that by performing Fourier transformation, the swap angle and phase angles are fully decoupled in terms of the dependencies in amplitude and phase. Furthermore, the analysis in Section Supplementary Information 4 reveals that the noise magnitude, namely, the variance of the noise, in the Fourier space is reduced by a factor of d. Consequently, these results suggest the design of algorithms in Fourier space.

According to Theorem 7 and Equation (27), the amplitudes of Fourier coefficients are exactly computable by solving

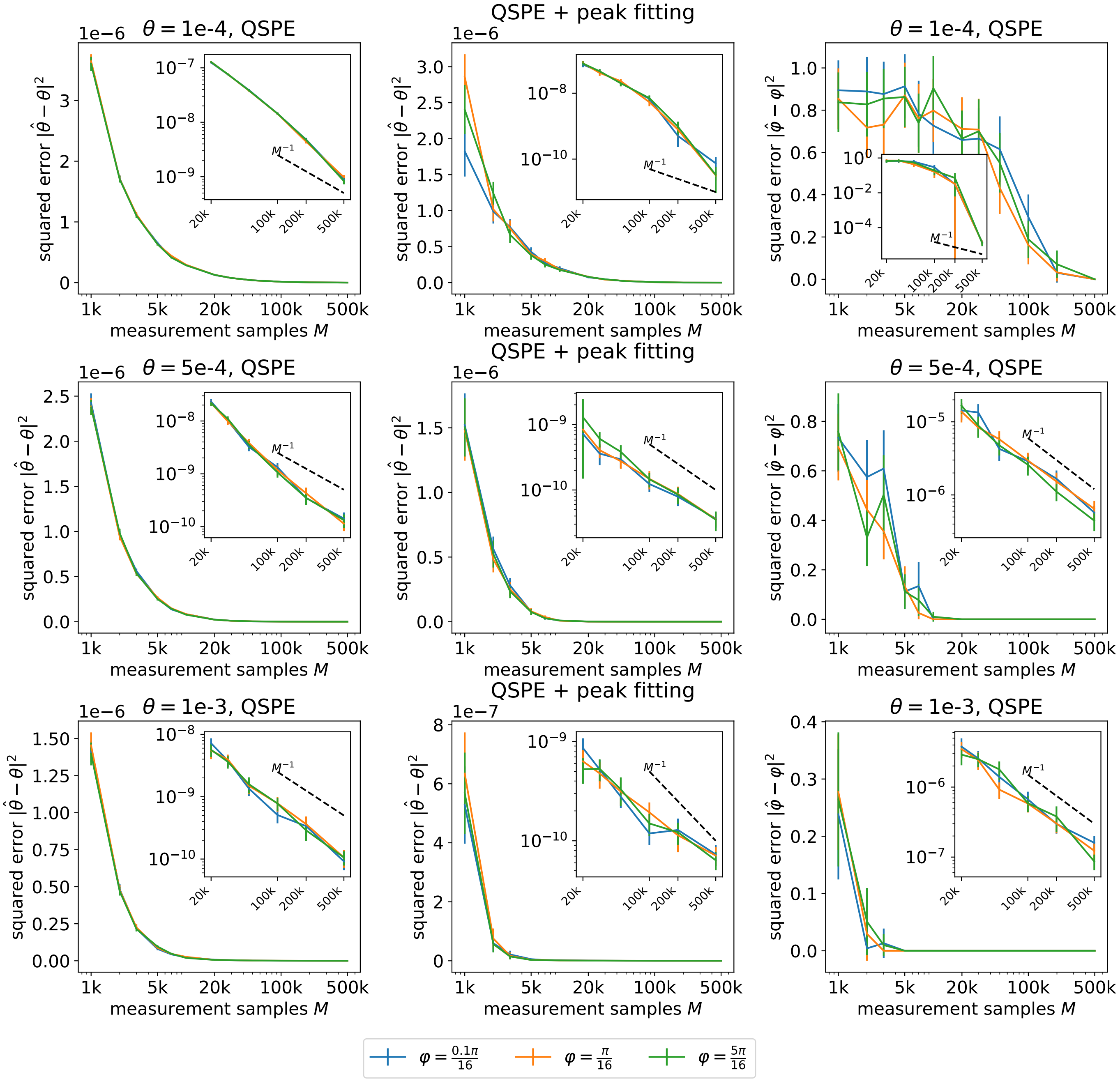

Supplementary Figure 6. Squared error of estimators as a function of the number of measurement samples. The only source of noise in the numerical experiments is the Monte Carlo sampling error. The circuit degree is set to $d = 50$ and the FsimGate phase parameter is set to $\chi = 5\pi/32$. $n_{\mathrm{pf}} = 15$ is used in the peak fitting. The error bar of each point stands for the confidence interval derived from 96 independent repetitions.

the integral:

$$A_k(\theta) := \widetilde{c}_k(\theta) = \frac{\sin\theta}{\pi} \int_0^{\pi} e^{-2\mathrm{i}(k+1)\omega} P_\omega^{(d)}(\cos\theta) Q_\omega^{(d)}(\cos\theta) \mathrm{d}\omega. \tag{122}$$

It is worth noting that the integrand is a finite-degree Laurent polynomial in terms of $e^{\pm \mathrm{i}\omega}$. Consequently, these coefficients can be exactly computed efficiently using $\mathcal{O}(d\log(d))$ floating-point operations. Given the experimental amplitudes $\{|c_k^{\exp}| : k = -d+1, \cdots, d-1\}$, the problem can be modeled as a system of nonlinear equations:

$$A_k(\theta) = \alpha_k \cdot |c_k^{\exp}|, \quad k = -d+1, \cdots, d-1. \tag{123}$$

Here, $\alpha_k \in \{-1, 1\}$ is an undetermined sign which is dropped when taking the amplitude. When formulating the nonlinear equation, we manually add it back. The set of candidate solutions of the k-th equation is $A_k^{-1}(\alpha_k \cdot |c_k^{\exp}|)$ which may contain more than one values as A_k may not be injective. However, the solution should satisfy all equations. Hence, the solution set is $\cup_{\{\alpha_k\}} \cap_{k=-d+1}^{d-1} A_k^{-1}(\alpha_k \cdot |c_k^{\exp}|)$. The difficulties of solving this problem lie in three major aspects:

1. Sign problem. The undetermined sign factor renders it different from classic nonlinear equation problems. There are seemingly 2^{2d-1} potential systems of nonlinear equations associated with $\{\alpha_k\}$ to be solved. However, most of them do not have solution, namely, $\cap_{k=-d+1}^{d-1} A_k^{-1}(\alpha_k \cdot |c_k^{\exp}|) = \emptyset$.

2. Nonconvexity. Each function A_k is nonconvex which contributes to the difficulty in solving nonlinear equation. Furthermore, the function is not injective which makes A_k^{-1} multi-valued. These render the numerical solution to the system of nonlinear equations challenging.

3. Imperfection due to noise and error. It is worth noting that the experimental values $\{|c_k^{\exp}|\}$ may not match exact values due to the presence of sampling error and noise. The deviation from the exact value might make taking intersection among solutions to each equation hard because they may not match exactly. To account for noise and error, and to make the solution robust, we may slightly relax the range of the solution $\mathcal{S}_{k,\gamma}(\alpha_k \cdot |c_k^{\exp}|) := \{A_k^{-1}(\alpha_k \cdot |c_k^{\exp}|) + x : |x| \leq \gamma\}$. Then, the set of relaxed solution is $\cup_{\{\alpha_k\}} \cap_{k=-d+1}^{d-1} \mathcal{S}_{k,\gamma}(\alpha_k \cdot |c_k^{\exp}|)$. The relaxation radius γ accounts for the uncertainty due to potential noise and error. It is worth noting that the choice of the relaxation radius γ is a tradeoff between noise magnitude and solution precision which is also referred to as bias-variance tradeoff. When γ is large, the variance of the solution is small which is robust, but the bias of the solution is large. Conversely, when γ is small, the variance of the solution is large as it is more vulnerable to noise but the bias is small.

It is worth noting that this hardness does not apply to the case when $d\theta \leq 1$. According to the analysis in the previous sections, $A_k(\theta) \approx \theta \mathbb{I}_{k \geq 0}$ which is linear and positive. Hence, the sign problem and the nonconvexity in general cases are not applicable. The third issue is resolved by taking average in the construction of the statistical estimator which is proven to be robust against sampling error.

As a consequence of the previous discussion, we can consider a simple interval-based solution to the general problem which approximately costs only $\mathcal{O}(d\epsilon^{-1})$ operations where ϵ is the target precision. The procedure of the algorithm is as follows. Let the full θ-range partitioned into small intervals $\{[l_i, r_i) : r_i - l_i \leq \epsilon\}$. Then, by continuity, we are able to determine whether there exists points in this interval satisfying certain nonlinear equation by examining $\min\{A_k(l_i), A_k(r_i)\} \leq \alpha_k \cdot |c_k^{\exp}| \leq \max\{A_k(l_i), A_k(r_i)\}$ for $\alpha_k = \pm 1$. We maintain a counter to count the number of satisfactions in each interval. The counter can be derived with $\mathcal{O}(d \log(d) \epsilon^{-1})$ operations. Finally, we scan all intervals to get all intervals with full $2d-1$ satisfactions of equations and set the potential solution to $\theta = (l_i + r_i)/2$.

This procedure is depicted in Figure 7 where each panel stands for an individual nonlinear equation with two distinct choices of $\alpha_k = \pm 1$. In each panel, all intersection points form the potential solution set $A_k^{-1}(\alpha_k \cdot |c_k^{\exp}|)$. As the degree parameter is set to $d = 5$, the system has nine nonlinear equations. By solving them using the previously discussed interval-based method, we obtain the counter which is visualized as a histogram in Figure 8. We see the full satisfaction is attained at $\theta = 1$ and $\theta = \pi - 1$. They form the final solution output of the algorithm. It is worth noting that these two θ-values are equivalent as the distinction in negating cosine can be accounted by redefining other phase angles.

To estimate phase angle φ, we note that Theorem 7 indicates that the φ-estimation procedure in the general case is identical to that when θ is small. Hence, we may still use the derived estimator to estimate φ by taking the sequential phase difference in the phases of Fourier coefficients. However, we note that the θ-value may affect the estimation accuracy of φ as the value of $A_k(\theta)$ modulates the magnitude of the Fourier mode and limits the signal-to-noise ratio as revealed in the analysis in Section Supplementary Information 4.

In Figure 9, we test the algorithm performance with variable θ-values. We see that the absolute error in θ-estimation remains well bounded below 5×10^{-4} except for the singularity near $\pi/2$. At this value, the variable part of the transition probabilities vanishes and $\mathfrak{h} = 0$. Hence, angle inference becomes increasingly challenging due to the absence of information. It is also worth noting that as θ gets close to $\pi/2$, the magnitude of $\cos(\theta)$ becomes more vanishing. Hence, the signal strength of the phase angle dependent part is increasingly weakened compared to noise. This low signal-to-noise ratio leads to the increase in the φ-estimation error in Figure 9.

Supplementary Information 6. Lower bounding the performance of QSPE

In the designed phase estimation algorithm, gate parameters are estimated from experimental data by running $2(2d-1)$ quantum circuits whose depths are $\Theta(d)$. If we simply think under the philosophy of the Heisenberg limit

Algorithm2 Inferring unknown angles in U-gate with general swap angle using QSPE procedure

Input: A U-gate $U(\theta,\varphi,\chi,*)$, an integer d (the number of applications of the U-gate), a precision parameter ϵ.
Output: Estimates $\hat{\theta},\hat{\varphi}$

Initiate a complex-valued data vector $\mathfrak{h}^{\exp}\in\mathbb{C}^{2d-1}$.
for $j=0,1,\cdots,2d-2$ **do**
  Set the tunable Z-phase modulation angle as $\omega_j=\frac{j}{2d-1}\pi$.
  Perform the quantum circuit in Figure 1 in the main text (or Figure 3) and measure the transition probabilities $p_X^{\exp}(\omega_j)$ and $p_Y^{\exp}(\omega_j)$.
  Set $\mathfrak{h}_j^{\exp}\leftarrow p_X^{\exp}(\omega_j)-\frac{1}{2}+\mathrm{i}\left(p_Y^{\exp}(\omega_j)-\frac{1}{2}\right)$.
end for
Compute the Fourier coefficients $\mathbf{c}^{\exp}=\mathsf{FFT}\left(\mathfrak{h}^{\exp}\right)$.
Compute estimates $\hat{\varphi}$ according to Definition 15 using $\mathsf{phase}(\mathbf{c}^{\exp})$.
Set number of intervals to $m=\lceil\pi/\epsilon\rceil$ and initiate a all-zero counter $z\in\mathbb{R}^m$.
Set $A_k^{(l)}=0$ for $k=-d+1,\cdots,d-1$
for $j=0,\cdots,m-1$ **do**
  Set $l=j\pi/m$ and $r=(j+1)\pi/m$.
  Set $A_k^{(r)}=A_k(r)$ which is derived by solving Equation (122) with FFT.
  for $\alpha_k=\pm 1$ **do**
    if $\min\{A_k^{(l)},A_k^{(r)}\}\le\alpha_k\cdot|c_k^{\exp}|\le\max\{A_k^{(l)},A_k^{(r)}\}$ **then**
      $z_j\leftarrow z_j+1$
    end if
  end for
  Set $A_k^{(r)}\leftarrow A_k^{(l)}$.
end for
Initiate a list $\hat{\theta}=[]$ and set $z_{\max}=\max_j z_j$.
for $j=0,\cdots,m-1$ **do**
  if $z_j=z_{\max}$ **then**
    Append $(j+1/2)\pi/m$ into $\hat{\theta}$.
  end if
end for

of quantum metrology in Ref. [9], we would expect the variance of statistical estimators bounded from below as

$$\Omega\left(1/\left((\text{classical repetition})\times(\text{quantum repetition})^2\right)\right)=\Omega(1/d^3)$$

when d is large enough. However, theoretical analysis in Theorem 2 in the main text and numerical simulation in Figure 5 show that the variance of the φ-estimator in QSPE depends on the parameter d as $\mathrm{Var}\left(\hat{\varphi}\right)\sim 1/d^4$. In this section, we will analyze this nontrivial counterintuitive result. In the end, we prove that for a fixed unknown FsimGate, the $1/d^4$-dependency only appears in the pre-asymptotic regime where the condition of the theorems holds, i.e., $d\theta\ll 1$. When passing to the limit of large enough d, the variances of statistical estimators agree with that suggested by the Heisenberg limit. Although such faster than Heisenberg limit scaling only applies in a finite range of circuit depth ($d\theta\ll 1$), it has drastically increased our metrology performance in practice against time-dependent errors, and thus deserves further investigation in its generalization to other domains of noise learning.

A. Pre-asymptotic regime $d\ll 1/\theta$

We derive the optimal variance scaling permitted using our metrology method in finite circuit depth, i.e. pre-asymptotic regime in this subsection. More particularly, we require that for a given range of gate parameter $\theta\in[\theta_{\min},\theta_{\max}]$, our metrology circuit depth obeys: $d\ll 1/\theta_{\min}$ in the pre-asymptotic regime. This also implies that for any θ under the consideration we have $d\theta\ll 1$.

The quantum circuits in QSPE form a class of parametrized quantum circuits whose measurement probabilities are trigonometric polynomials in a tunable variable ω. For simplicicty, the gate parameters of the unknown FsimGate is denoted as $\Xi=(\xi_k)=(\theta,\varphi,\chi)$. According to the modeling of Monte Carlo sampling error in Lemma 11, the experimentally estimated probabilities are approximately normal distributed. Given the normality and assuming the limit $M\gg 1$, the element of the Fisher information matrix is

$$I_{kk'}(\Xi)=\sum_{j=0}^{2d-2}\Sigma_{X,j}^{-2}\frac{\partial p_X(\omega_j;\Xi)}{\partial\xi_k}\frac{\partial p_X(\omega_j;\Xi)}{\partial\xi_{k'}}+\sum_{j=0}^{2d-2}\Sigma_{Y,j}^{-2}\frac{\partial p_Y(\omega_j;\Xi)}{\partial\xi_k}\frac{\partial p_Y(\omega_j;\Xi)}{\partial\xi_{k'}}.\tag{124}$$

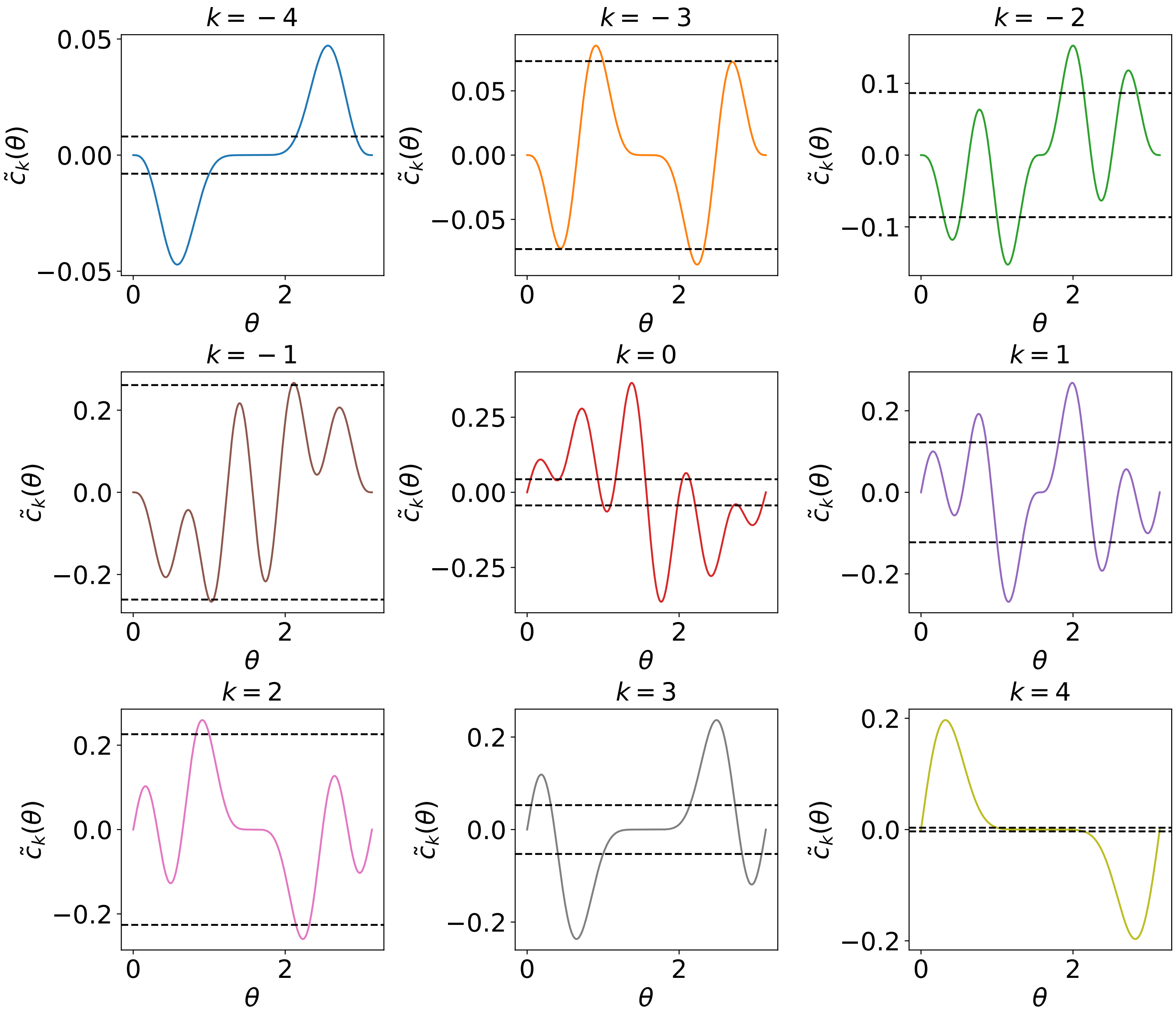

Supplementary Figure 7. An example of formulating QSPE as solving systems of nonlinear equations. We set $d = 5$ and $M = 1 \times 10^5$. The relevant angles are set to $\theta = 1, \varphi = \pi/16$. In each panel, the solid curve is the amplitude of the Fourier coefficients derived by solving the defining integral. The two horizontal dashed lines stand for the experimental amplitude with positive or negative signs ($\alpha_k = \pm 1$). The intersecting points are candidate solutions $A_k^{-1}(\alpha_k \cdot |c_k^{\mathrm{exp}}|)$.

According to Equation (50), the variance of the Monte Carlo sampling error concentrates near a constant. Hence

$$I_{kk'}(\Xi) = 4M\left(1 + \mathcal{O}(d^2\theta^2)\right) \sum_{j=0}^{2d-2} \left(\frac{\partial p_X(\omega_j;\Xi)}{\partial \xi_k} \frac{\partial p_X(\omega_j;\Xi)}{\partial \xi_{k'}} + \frac{\partial p_Y(\omega_j;\Xi)}{\partial \xi_k} \frac{\partial p_Y(\omega_j;\Xi)}{\partial \xi_{k'}} \right). \tag{125}$$

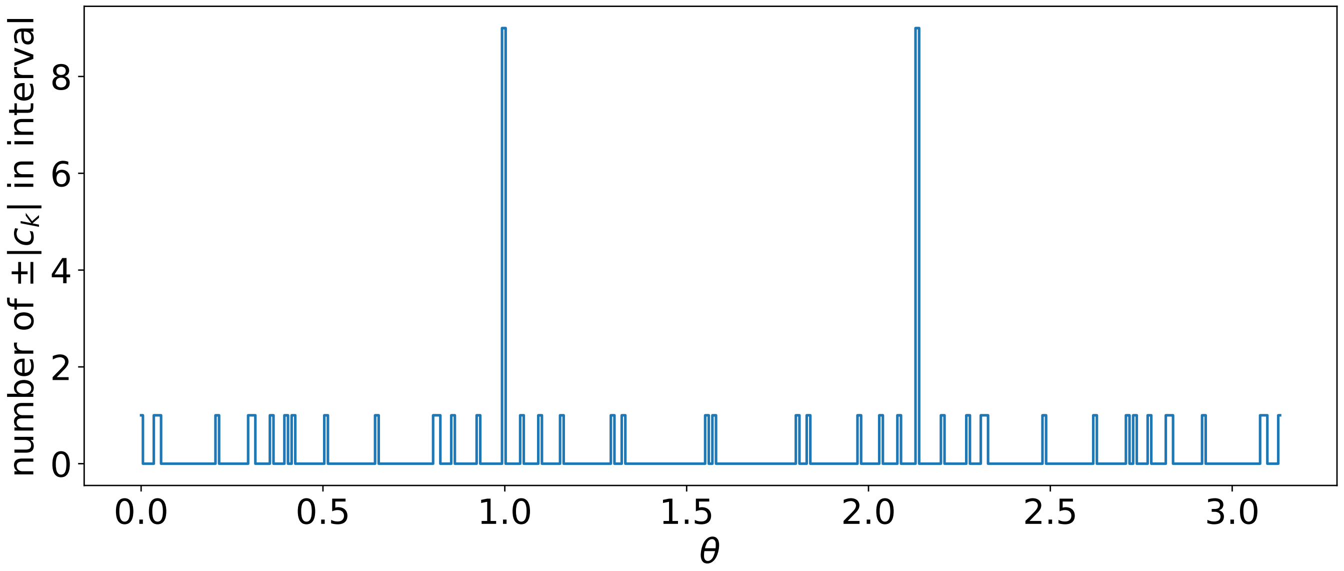

Supplementary Figure 8. An example of the counter of interval-based algorithm for solving QSPE. The setup is identical to that in Figure 7. The θ-values satisfy all nine nonlinear equations are the output of the algorithm, which are estimators of the swap angle.

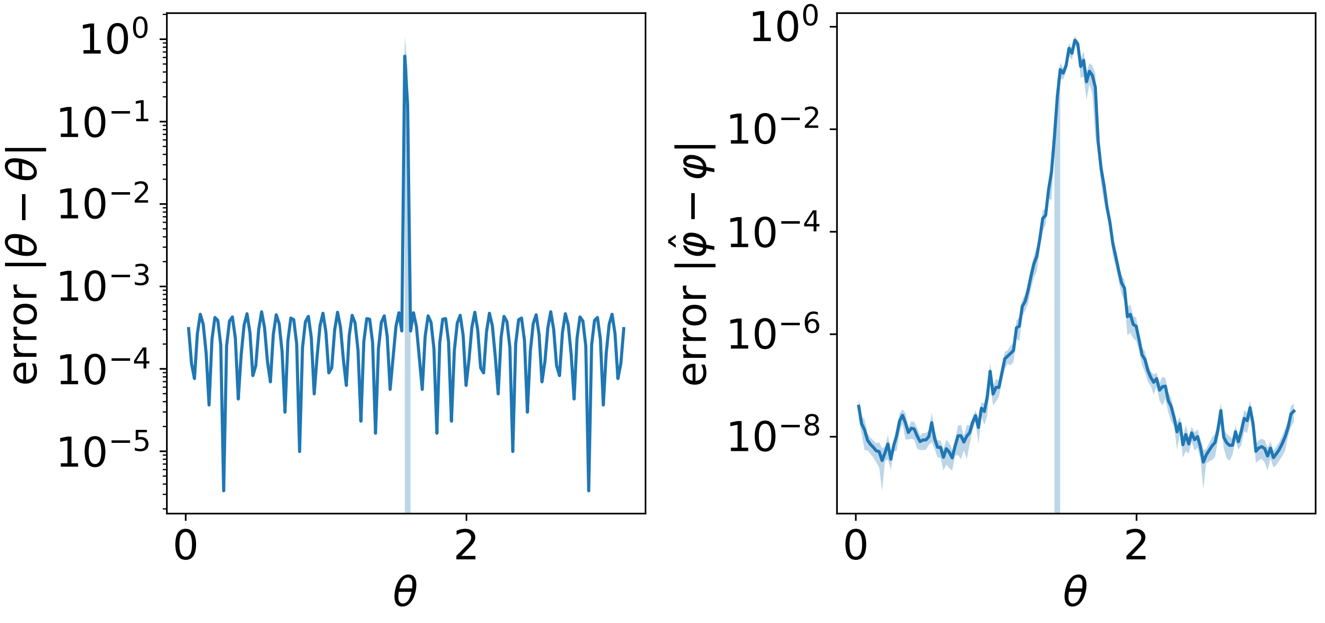

Supplementary Figure 9. Estimation errors of θ and φ using the generalized QSPE estimation algorithm for general swap angles. We set $d = 5$ and $M = 1 \times 10^5$. We fix $\varphi = \pi/16$ and make θ variable. The shaded area stands for the confidence interval (error bar) determined from ten independent repetitions.

Using the reconstructed function, the element of the Fisher information matrix can be expressed as

$$I_{kk'}(\Xi) = 4M\left(1+\mathcal{O}(d^2\theta^2)\right)\mathrm{Re}\left(\sum_{j=0}^{2d-2}\frac{\partial\mathfrak{h}(\omega_j;\Xi)}{\partial\xi_k}\frac{\partial\overline{\mathfrak{h}(\omega_j;\Xi)}}{\partial\xi_{k'}}\right) \tag{126}$$

$$= 4M(2d-1)\left(1+\mathcal{O}(d^2\theta^2)\right)\mathrm{Re}\left(\sum_{j=-d+1}^{d-1}\frac{\partial c_j(\Xi)}{\partial\xi_k}\frac{\partial\overline{c_j(\Xi)}}{\partial\xi_{k'}}\right) \tag{127}$$

$$= \frac{4M(2d-1)}{\pi}\left(1+\mathcal{O}(d^2\theta^2)\right)\mathrm{Re}\left(\int_{-\pi/2}^{\pi/2}\frac{\partial\mathfrak{h}(\omega;\Xi)}{\partial\xi_k}\frac{\partial\overline{\mathfrak{h}(\omega;\Xi)}}{\partial\xi_{k'}}\mathrm{d}\omega\right). \tag{128}$$

Here, we use the construction of QSPE in which the tunable angles are equally spaced in one period of the reconstructed function. The second equality (Equation (127)) invokes Theorem 3 and the discrete orthogonality of Fourier factors. The last equality (Equation (128)) is due to the Parseval's identity.

When $d\theta \ll 1$ and $\theta \ll 1$, the Fourier coefficients are well captured by the approximation in Theorem 3 which gives $c_j(\Xi) \approx \mathrm{i}e^{-\mathrm{i}\chi}e^{-\mathrm{i}(2j+1)\varphi}\theta\mathbb{I}_{j\geq 0}$. Consequentially, using Equation (127), in the pre-asymptotic regime $d \ll 1/\theta$, the Fisher information matrix is approximately

$$I(\Xi) \approx 4M(2d-1)\begin{pmatrix} d & 0 & 0 \\ 0 & \frac{d(4d^2-1)}{3}\theta^2 & d^2\theta^2 \\ 0 & d^2\theta^2 & d\theta^2 \end{pmatrix}. \tag{129}$$

Invoking Cramér-Rao bound, the covariance matrix of any statistical estimator is lower bounded as

$$\mathrm{Cov}\left(\hat{\theta}_{\mathrm{any}},\hat{\varphi}_{\mathrm{any}},\hat{\chi}_{\mathrm{any}}\right) \succeq I^{-1}(\Xi) \approx \frac{1}{4Md(2d-1)}\begin{pmatrix} 1 & 0 & 0 \\ 0 & \frac{3}{(d^2-1)\theta^2} & -\frac{3d}{(d^2-1)\theta^2} \\ 0 & -\frac{3d}{(d^2-1)\theta^2} & \frac{4d^2-1}{(d^2-1)\theta^2} \end{pmatrix}. \tag{130}$$

Consequentially, in the pre-asymptotic regime, the optimal variances of the statistical estimator are

$$\mathrm{Var}\left(\hat{\theta}_{\mathrm{opt}}\right) = \frac{1}{4Md(2d-1)} \approx \frac{1}{8Md^2}, \tag{131}$$

$$\mathrm{Var}\left(\hat{\varphi}_{\mathrm{opt}}\right) = \frac{3}{4Md(2d-1)(d^2-1)\theta^2} \approx \frac{3}{8Md^4\theta^2}, \tag{132}$$

$$\mathrm{Var}\left(\hat{\chi}_{\mathrm{opt}}\right) = \frac{1}{4Md(2d-1)\theta^2}\frac{4d^2-1}{d^2-1} \approx \frac{1}{2Md^2\theta^2}. \tag{133}$$

Remarkably, the variances of QSPE estimators in Theorem 2 in the main text exactly match the optimality given in Equations (131) and (132). We thus proves the optimality of our QSPE estimator for inferring gate parameter θ and φ. Moreover, we like to point out that the faster than Heisenberg-limit scaling of parameter φ in this asymptotic regime is critical to the successful experimental deployment of our methods. This is because the dominant time-dependent error results in a time-dependent drift error in φ, and a faster convergence in circuit depth provides faster metrology runtime to minimize such drift error during the measurements.

B. Asymptotic regime $d \to \infty$

Thinking under the framework of Heisenberg limit in Ref. [9], for a fixed θ, the optimal variances of θ and φ estimators are expected to scale as $1/d^3$ while that of χ estimator scales as $1/d$ due to the absence of amplification in the quantum circuit. In contrast to these scalings, we show in the last subsection that the scalings of φ and χ estimators can achieve $1/d^4$ and $1/d^2$ in the pre-asymptotic regime $d \ll 1/\theta$. In this subsection, we will argue that the scalings predicted by the Heisenberg scaling hold if further passing to the asymptotic limit $d \to \infty$. As a consequence, there is a nontrivial transition of variance scalings of QSPE estimators in pre-asymptotic regime and the asymptotic regime. We demonstrate such subtle transition in the fundamental efficiency allowed for the given metrology protocol with both numerical simulation and analytic reasoning in this section.

As $d \to \infty$, the measurement probabilities no longer admit the property of concentration around constants. Using the variance derived in Equation (50), the diagonal element of Fisher information matrix is exactly equal to

$$\begin{aligned} I_{kk}(\Xi) &= M \sum_{j=0}^{2d-2} \left(\frac{1}{p_X(\omega_j;\Xi)\left(1-p_X(\omega_j;\Xi)\right)} \frac{\partial p_X(\omega_j;\Xi)}{\partial \xi_k} \frac{\partial p_X(\omega_j;\Xi)}{\partial \xi_k} \right. \\ &\qquad \left. + \frac{1}{p_Y(\omega_j;\Xi)\left(1-p_Y(\omega_j;\Xi)\right)} \frac{\partial p_Y(\omega_j;\Xi)}{\partial \xi_k} \frac{\partial p_Y(\omega_j;\Xi)}{\partial \xi_k} \right) \\ &= M \sum_{j=0}^{2d-2} \left(-\frac{\partial \log p_X(\omega_j;\Xi)}{\partial \xi_k} \frac{\partial \log\left(1-p_X(\omega_j;\Xi)\right)}{\partial \xi_k} - \frac{\partial \log p_Y(\omega_j;\Xi)}{\partial \xi_k} \frac{\partial \log\left(1-p_Y(\omega_j;\Xi)\right)}{\partial \xi_k} \right). \end{aligned} \tag{134}$$

Moreover $p_X(\omega_j;\Xi)$ and $p_Y(\omega_j;\Xi)$ are trigonometric polynomials in θ and φ of degree at most d while in χ of degree 1 due to the absence of amplification. Therefore the log-derivatives of θ and φ are $\mathcal{O}(d)$ in most regular cases while they are $\mathcal{O}(1)$ for χ. Hence, we expect from the Cramér-Rao bound that

$$\mathrm{Var}\left(\hat{\theta}_{\mathrm{opt}}\right), \mathrm{Var}\left(\hat{\varphi}_{\mathrm{opt}}\right) = \Omega\left(\frac{1}{d^3}\right), \quad \text{and } \mathrm{Var}\left(\hat{\chi}_{\mathrm{opt}}\right) = \Omega\left(\frac{1}{d}\right) \quad \text{as } d \to \infty. \tag{135}$$

These results match the scalings predicted by the Heisenberg limit which holds in the asymptotic limit $d \to \infty$.

C. Numerical results

We compute the Cramér-Rao lower bound (CRLB) of the statistical inference problem defined by QSPE. The lower bound is given by the diagonal element of inverse Fisher information matrix

$$\mathrm{CRLB}\left(\hat{\xi}_k\right) = \left(I^{-1}(\Xi)\right)_{kk} \tag{136}$$

where the Fisher information matrix is element-wisely defined in Equation (124). At the same time, we also compute the approximation to the optimal variance in the pre-asymptotic regime $d \ll 1/\theta$ derived in Equations (131) to (133). The numerical results are given in Figure 3 (b) in the main text. It can be seen that the approximated optimal variance agrees very well with the exact CRLB. In the asymptotic regime with large enough d, the optimal variance scaling given by the CRLB is as predicted in Equation (135). Furthermore, the numerical results validate that there exists a nontrivial transition around $d \approx 1/\theta$ making the optimal variance scalings completely different in the pre-asymptotic and asymptotic regime.

To prove the optimality of QSPE and investigate the situation where the conditions for deriving QSPE hold, we numerically estimate the variances of QSPE estimators and compare them with the derived optimal variances in the pre-asymptotic regime in Equations (131) and (132). The QSPE estimators are derived by approximating the original statistical inference problem by a linear model. When d gets large, the model violation due to the approximation contributes to the bias of QSPE estimators. We compute the mean-square error (MSE) and using the bias-variance decomposition MSE $=$ Var $+$ bias^2 to quantify the bias. The numerical results are displayed in Figure 10. Our simulation shows that the bias of θ-estimator dominates the MSE and contaminates the inference accuracy after d becomes larger than a threshold determined by the pre-asymptotic regime $d\theta \ll 1$. Despite the bias due to the model violation, the MSE of the θ-estimator still achieves some accuracy of order θ^2 which suggests that the θ-estimator might give a reasonable estimation of a similar order with model violation in larger d. The numerical results show that the φ-estimator is more robust where the MSE deviates significantly from the theoretical scaling in the pre-asymptotic regime after $d \geq 1/\theta$ is large enough to pass to the asymptotic regime. Furthermore, the MSE well matches the variance which implies that the bias in φ-estimator is always small. The difference in the robustness of the θ- and φ-estimators is credited to the construction of QSPE in which the inferences of θ and φ are completely decoupled due to the data post-processing using FFT.

Figure 10 and Figure 3 (b) in the main text suggest the following. (1) In the pre-asymptotic regime, QSPE estimators achieve the optimality in the sense of saturating the Cramér-Rao lower bound and exhibit robustness against time-dependent errors in φ in both simulation and experimental deployments. Furthermore, the construction of QSPE estimators only involves direct algebraic operations rather than iterative optimization, and the reduced inference problems in Fourier space are linear statistical models whose global optimum is unique for each realization. This not only enables the fast and reliable data post-processing but also allows us to analyze its performance analytically. (2) Passing to the asymptotic regime, given the significant bias of θ-estimator and the sharp transition of the variance of

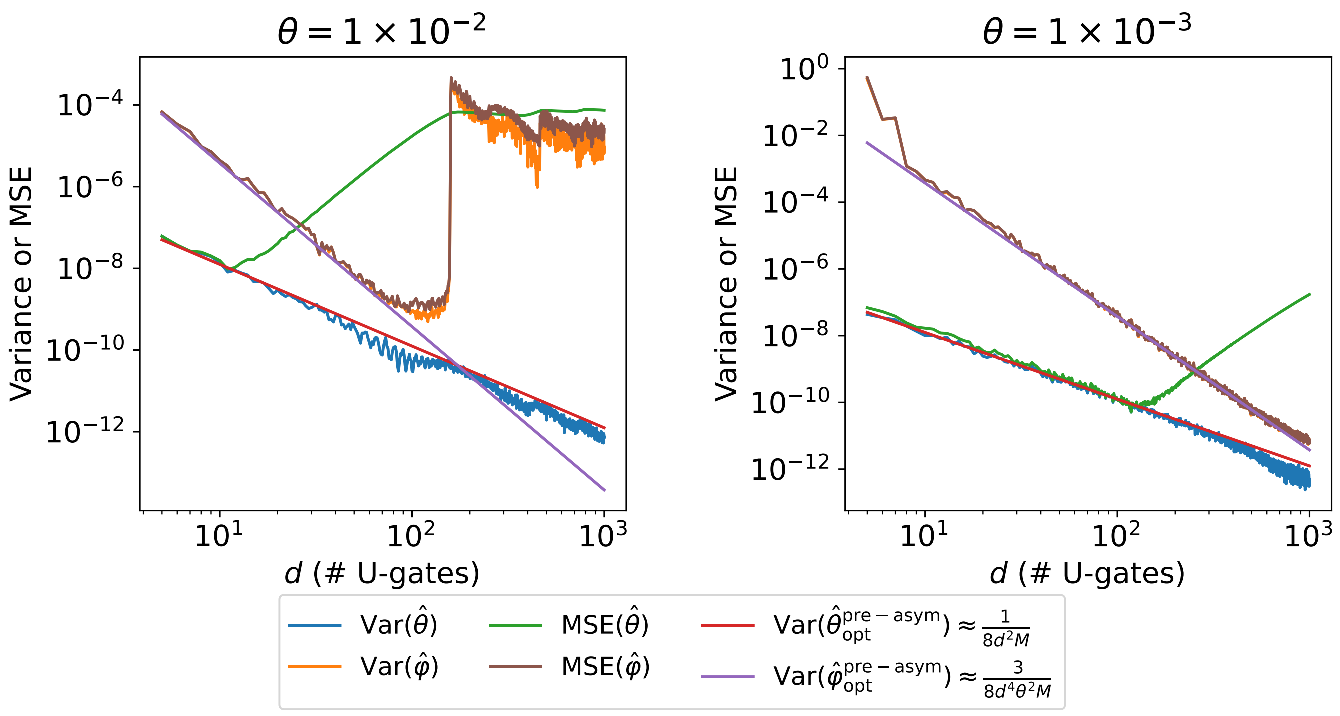

Supplementary Figure 10. Variance and mean-square error (MSE) of QSPE estimators. The left panel corresponds to the case where θ is relatively large and $d\theta \ll 1$ condition fails quickly at around $d = 10$, beyond which bias dominates the estimator's MSE since our inference model assumption ($d\theta \ll 1$) fails. The right panel corresponds to the case where $d\theta \ll 1$ condition holds all the way to around $d = 100$. The single-qubit phases are set to $\varphi = \pi/16$ and $\chi = 5\pi/32$. The number of measurement samples is set to $M = 1 \times 10^5$. Each data point is derived from 100 independent repetitions.

φ-estimator, one has to use other estimators to saturate the optimal variance scaling and unbiasedness, for example, maximum-likelihood estimators (MLE). Furthermore, the analysis based on the Cramér-Rao lower bound is made by fixing the data generation (measuring quantum circuits) but varying data post-processing.

D. Comparing with quantum Cramér-Rao lower bound

As a quantum analog of classical Fisher information, quantum Fisher information (QFI) lies in the center of quantum metrology by providing a fundamental lower bound on the accuracy one can infer from the system of a given resource limit.

In general case, the QFI is defined as

$$\mathfrak{F}(\theta) := \mathrm{tr}\left(\varrho(\theta) L_\varrho^2(\theta)\right) \tag{137}$$

where the symmetric logarithmic derivative $L_\varrho(\theta)$ is defined implicitly [5, 13]. For a system whose density matrix evolves as $\varrho(\theta) = e^{\mathrm{i}\theta\mathcal{H}}\varrho_0 e^{-\mathrm{i}\theta\mathcal{H}}$, the QFI can be explicitly computed by diagonalizing $\varrho(\theta)$. According to the analysis in [13], the QFI is an upper bound on the Fisher information over all possible measurements. For brevity, we only consider the inference of θ and hold all other unknown parameters constant in the analysis. However, our analysis can be generalized to the multiple parameter inference by adopting the multi-variable QFI in Ref. [13].

In our case, the collection of quantum circuits with variable modulation angle ω can be written in a density matrix which is a uniform average over all circuit realizations, namely

$$\varrho(\theta) = \frac{1}{2d-1} \sum_{j=0}^{2d-2} \mathcal{U}^{(d)}(\omega_j; \theta, \varphi, \chi) \varrho_0 \mathcal{U}^{(d)}(\omega_j; \theta, \varphi, \chi)^\dagger. \tag{138}$$

Note that the optimization is intended to be performed over all potential initialization and measurement. Given

that only θ is considered and other angle parameters are held constant, it suffices to consider a simpler case

$$\varrho(\theta)=\frac{1}{2d-1}\sum_{j=0}^{2d-2}U^{(d)}(\omega_j-\varphi,\theta)\varrho_0 U^{(d)}(\omega_j-\varphi,\theta)^\dagger. \tag{139}$$

According to Equation (17), this alternative density matrix is equivalent to absorbing some constant rotation gates into initialization and measurement. When $\theta\ll 1$ is small, the following expansion holds:

$$U^{(d)}(\omega,\theta)=I+\mathrm{i}\theta\sum_{k=1}^{d}e^{\mathrm{i}k\omega Z}Xe^{\mathrm{i}(d-k+1)\omega Z}+\mathcal{O}((d\theta)^2)=I+\mathrm{i}\theta\underbrace{X\sum_{k=1}^{d}e^{\mathrm{i}(d-2k+1)\omega Z}}_{\mathcal{H}(\omega)}+\mathcal{O}((d\theta)^2). \tag{140}$$

Note that $\mathcal{H}(\omega)$ is Hermitian because

$$\mathcal{H}^\dagger(\omega)=\sum_{k=1}^{d}e^{-\mathrm{i}(d-2k+1)\omega Z}X=X\sum_{k=1}^{d}e^{\mathrm{i}(d-2k+1)\omega Z}=\mathcal{H}(\omega). \tag{141}$$

Furthermore,

$$\mathcal{H}(\omega)=X\mathrm{diag}\left\{\sum_{k=1}^{d}e^{\mathrm{i}(d-2k+1)\omega},\sum_{k=1}^{d}e^{-\mathrm{i}(d-2k+1)\omega}\right\}=X\frac{e^{\mathrm{i}d\omega}-e^{-\mathrm{i}d\omega}}{e^{\mathrm{i}\omega}-e^{-\mathrm{i}\omega}}=XU_{d-1}(\cos(\omega)) \tag{142}$$

where U_{d-1} is the Chebyshev polynomial of the second kind. Then, when $d\theta\ll 1$ is small enough, it approximately holds that

$$\varrho_j(\theta):=U^{(d)}(\omega_j-\varphi,\theta)\varrho_0 U^{(d)}(\omega_j-\varphi,\theta)^\dagger=\varrho_0+\mathrm{i}\theta\left[\mathcal{H}(\omega_j-\varphi),\varrho_0\right]+\mathcal{O}((d\theta)^2)\approx e^{\mathrm{i}\theta\mathcal{H}(\omega_j-\varphi)}\varrho_0 e^{-\mathrm{i}\theta\mathcal{H}(\omega_j-\varphi)}. \tag{143}$$

Then, as a two-dimensional density matrix, its QFI is

$$\mathfrak{F}_j(\theta):=4\frac{(\lambda_0-\lambda_1)^2}{\lambda_0+\lambda_1}\left|\langle\psi_0|\,\mathcal{H}(\omega_j-\varphi)\,|\psi_1\rangle\right|^2 \tag{144}$$

where $\lambda_i,|\psi_i\rangle$ are the eigenvalue and eigenvector of the density matrix. It is upper bounded as

$$\mathfrak{F}_j(\theta)\le 4\|\mathcal{H}(\omega_j-\varphi)\|_2^2=4U_{d-1}^2(\cos(\omega_j-\varphi)). \tag{145}$$

Because the overall density matrix is a uniform combination of $\varrho_j(\theta)$ and all generator Hamiltonians are scaled Pauli X operators, the convexity of the QFI implies that

$$\mathfrak{F}(\theta)\le\frac{1}{2d-1}\sum_{j=0}^{2d-2}\mathfrak{F}_j(\theta)\le\frac{4}{2d-1}\sum_{j=0}^{2d-2}U_{d-1}^2(\cos(\omega_j-\varphi))=4d \tag{146}$$

where Lemma 18 is used. Note that the upper bound on the QFI derived here is independent of the initialization ϱ, and the formalism of QFI provides a bound on the inference variance regardless of the choice of measurements. Hence, we achieve a lower bound on the variance of $\hat{\theta}$ with respect to any initialization, measurement, and classical data processing by invoking the quantum Cramér-Rao bound:

$$\inf\mathrm{Var}(\hat{\theta})\ge\frac{1}{M2(2d-1)\mathfrak{F}(\theta)}\ge\frac{1}{8Md(2d-1)} \tag{147}$$

where it uses the fact that there are $2(2d-1)M$ experiments in total.

Before closing the analysis of inference error, we could gain more understanding of our method from the lower bounds derived in this section.

1. Compared with the result in Equation (131) derived by applying classical Cramér-Rao bound, we see that the bound in Equation (147) derived from QFI is lower, namely, quantum bound $=0.5\times$ classical bound. This differentiation is explainable. Note that we use two logical Bell states to perform experiments. The advantage is the experimental probabilities of these two experiments form a conjugate pair to reconstruct a complex function

that for the ease of analysis. This complex function and its properties (see Theorems 7 and 9) eventually lead to a simple robust statistical estimator requiring only light computation. In contrast, the data generated from the initialization of one Bell state still contains full information of the parameters to be estimated. However, the highly nonlinear dependency renders the practical inference challenging. Hence, the factor of 2 is due to the use of a pair of Bell states. Although the QFI indicates that inference variance can be lower by removing such redundancy in the initialization, the nature of ignoring practical ease makes it hard to achieve.

2. The importance of the phase matching condition is also reflected in the analysis of QFI. It is worth noting that when we set $\omega_j = \varphi$, the QFI of a single experiment is $\mathfrak{F}_j(\theta)|_{\omega_j=\varphi} \leq 4U_{d-1}^2(1) = 4d^2$ which attains the maximum of the Chebyshev polynomial. However, in practice, due to the absence of accurate information of φ, our method samples the data on equally spaced ω points and processes the data via FFT to isolate the dependencies of θ and φ. This further sampling procedure averages the QFI and lowers its value from $4d^2$ to $4d$.

Lemma 18. *Given an integer d and any $\varphi \in \mathbb{R}$, it holds that*

$$\frac{1}{2d-1} \sum_{j=0}^{2d-2} U_{d-1}^2(\cos(\omega_j - \varphi)) = d \tag{148}$$

where $\omega_j := j\pi/(2d-1)$ where $j = 0, \cdots, 2d-2$.

Proof. Note that the discrete orthogonality implies that

$$\frac{1}{2d-1} \sum_{j=0}^{2d-2} U_{d-1}^2(\cos(\omega_j)) = \frac{1}{\pi} \int_0^\pi U_{d-1}^2(\cos(\omega - \varphi)) \mathrm{d}\omega = \frac{1}{\pi} \left(\int_0^\pi + \int_{-\varphi}^0 - \int_{\pi-\varphi}^\pi \right) U_{d-1}^2(\cos(\omega)) \mathrm{d}\omega. \tag{149}$$

Using the parity condition of Chebyshev polynomials, it holds that

$$\int_{\pi-\varphi}^\pi U_{d-1}^2(\cos(\omega)) \mathrm{d}\omega \overset{\omega \leftarrow \omega - \pi}{=} \int_{-\varphi}^0 \left((-1)^{d-1} U_{d-1}(\cos(\omega)) \right)^2 \mathrm{d}\omega = \int_{-\varphi}^0 U_{d-1}^2(\cos(\omega)) \mathrm{d}\omega. \tag{150}$$

Hence, for any $\varphi \in \mathbb{R}$, it holds that

$$\frac{1}{2d-1} \sum_{j=0}^{2d-2} U_{d-1}^2(\cos(\omega_j)) = \frac{1}{\pi} \int_0^\pi U_{d-1}^2(\cos(\omega)) \mathrm{d}\omega = \frac{1}{\pi} \int_{-1}^1 \frac{U_{d-1}^2(x)}{\sqrt{1-x^2}} \mathrm{d}x. \tag{151}$$

Using the product formula of Chebyshev polynomials, we have

$$U_{d-1}^2(x) = \sum_{p=0}^{d-1} U_{2p}(x) = \sum_{p=0}^{d-1} \left(1 + 2 \sum_{k=1}^p T_{2k}(x) \right). \tag{152}$$

Then, the weighted orthogonality of Chebyshev polynomials of the first kind implies that

$$\frac{1}{\pi} \int_{-1}^1 \frac{U_{d-1}^2(x)}{\sqrt{1-x^2}} \mathrm{d}x = \sum_{p=0}^{d-1} \left(1 + \frac{2}{\pi} \sum_{k=1}^p \int_{-1}^1 \frac{T_{2k}(x) T_0(x)}{\sqrt{1-x^2}} \mathrm{d}x \right) = d \tag{153}$$

which completes the proof. □

Supplementary Information 7. Analysis of realistic error

Although QSPE estimators are derived from modeling Monte Carlo sampling error, we numerically show their robustness against realistic errors in this section. This section is organized as follows. We discuss the sources of realistic errors including depolarizing error, time-dependent error, and readout error in each subsection. We study the methods for correcting some realistic errors by analyzing experimental data. Furthermore, we perform numerical experiments to validate the robustness of our proposed quantum metrology scheme.

A. Depolarizing error

The quantum error largely contaminates the signal. In the two-qubit system, we assume the quantum error is captured by a depolarizing quantum channel, where the density matrix is transformed to the convex combination of the correctly implemented density matrix and that of the uniform distribution on bit-strings. Therefore, assuming the infinite number of measurement samples (vanishing Monte Carlo sampling error), the measurement probability is

$$p_{X(Y)|\alpha}(\omega;\theta,\varphi,\chi) = \alpha p_{X(Y)}(\omega;\theta,\varphi,\chi) + \frac{1-\alpha}{4} \tag{154}$$

where $\alpha \in [0,1]$ is referred to as the circuit fidelity. Then, the sampled reconstructed function is also shifted and scaled accordingly $\mathfrak{h}_\alpha(\omega;\theta,\varphi,\chi) = \alpha\mathfrak{h}(\omega;\theta,\varphi,\chi) - \frac{1-\alpha}{4}(1+\mathrm{i})$. Consequentially, the Fourier coefficients are expected to be scaled by α simultaneously and the constant shift only contributes to the zero-indexed Fourier coefficient, namely

$$\left|c_{0|\alpha}^{\exp}\right| = \left|\alpha c_0^{\exp} - \frac{1-\alpha}{4}(1+\mathrm{i})\right| \approx \alpha\theta + \frac{1-\alpha}{2\sqrt{2}}, \quad \left|c_{k|\alpha}^{\exp}\right| \approx \alpha\theta,\ \forall k = 1,\cdots,d-1. \tag{155}$$

The approximation of $\left|c_{0|\alpha}^{\exp}\right|$ holds when the circuit fidelity is not close to one, namely, $\theta \ll 1-\alpha$. Yet when the circuit fidelity is close to one, the depolarizing error can be neglected as a higher-order effect. Using this feature, the circuit fidelity can be estimated from the difference between the Fourier coefficient of zero index and those of nonzero indices. Then, the estimators of the circuit fidelity and the swap angle are given by

$$\begin{aligned} \hat{\alpha} &= 1 - 2\sqrt{2}\left(\left|c_{0|\alpha}^{\exp}\right| - \frac{1}{d-1}\sum_{k=1}^{d-1}\left|c_{k|\alpha}^{\exp}\right|\right), \\ \hat{\theta} &= \frac{1}{\hat{\alpha}} \times \frac{1}{d-1}\sum_{k=1}^{d-1}\left|c_{k|\alpha}^{\exp}\right|. \end{aligned} \tag{156}$$

We numerically test the accuracy of these estimators in Section Supplementary Information 7 C. In Table 1, we list the effective depolarizing error rate on the single-excitation subspace inferred from the exponential decay of circuit fidelities derived from QSPE methods on our experimental measurements. These values agree with our estimation results using a conventional cross-entropy benchmark that is much slower to run and requires a $10X$ deeper circuit for randomization.

(3,6) and (3,7)	(3,6) and (4,6)	(3,7) and (4,7)	(4,5) and (4,6)	(4,7) and (5,7)
4.52×10^{-3}	4.73×10^{-3}	5.39×10^{-3}	4.69×10^{-3}	5.15×10^{-3}
(5,7) and (6,7)	(5,7) and (5,8)	(5,6) and (6,6)	(5,6) and (5,7)	(4,8) and (5,8)
8.25×10^{-3}	5.89×10^{-3}	2.81×10^{-3}	3.59×10^{-3}	4.96×10^{-3}
(5,8) and (5,9)	(5,8) and (6,8)	(6,6) and (7,6)	(6,8) and (7,8)	(7,5) and (7,6)
5.84×10^{-3}	5.70×10^{-3}	3.36×10^{-3}	5.13×10^{-3}	3.32×10^{-3}
(7,6) and (7,7)	(7,7) and (7,8)			
2.36×10^{-3}	2.89×10^{-3}			

Supplementary Table 1. Qubit pairs and the inferred effective error rate on the single-excitation subspace. The error rate is estimated by the regression with respect to the exponential decay. The regression data are the circuit fidelity estimated from QSPE in Figure 4 in the main text (top-right panel).

B. Time-dependent error

The dominant time-dependent noise in superconducting qubits two-qubit control is in the frequency of the qubits. It can be modeled by time-dependent Z phase error in FsimGate. Observed from experimental data, the magnitude of the time-dependent drift error increases when more gates are applied to the circuit. To emulate the realistic time-dependent noise, we model the noise by introducing a random deviation in angle parameters, which is referred to as the coherent angle uncertainty. Given a perfect FsimGate parametrized as $U_{\mathrm{FsimGate}}(\theta,\varphi,\chi,*)$, the erroneous quantum

gate due to the coherent angle uncertainty is another FsimGate parametrized as $U_{\mathrm{FsimGate}}(\theta_{\mathrm{unc}}, \varphi_{\mathrm{unc}}, \chi_{\mathrm{unc}}, *)$. Here, angle parameters subjected to the uncertainty are distributed uniformly at random around the perfect value

$$\theta_{\mathrm{unc}} \in [\theta - D_\theta, \theta + D_\theta],\ \varphi_{\mathrm{unc}} \in [\varphi - D_\varphi, \varphi + D_\varphi],\ \chi_{\mathrm{unc}} \in [\chi - D_\chi, \chi + D_\chi] \tag{157}$$

where $D_\theta, D_\varphi, D_\chi$ stand for the maximal deviations of uncertain parameters. Inspired by experimental results, maximum deviations of phase angles are increasing when more FsimGate's are applied. Moreover, there is a Gaussian noise [18] in the analog pulse realizations, causing small fluctuations on all gate parameters. To capture this feature and the rough estimate from the experimental data, we set the uncertainty model when the j-th FsimGate is applied as

$$D_\theta^{(j)} = 0.1 \times \theta,\ D_\varphi^{(j)} = D_\chi^{(j)} = 0.3 \times \frac{j}{d}. \tag{158}$$

Noticeably, the proposed model has already taken the phase drift in Z-rotation gates into account, which is effectively factored in the random phase drift in the single-qubit phase φ and χ in the FsimGate.

C. Numerical performance of the estimation against depolarizing error and time-dependent drift error

In the numerical simulation, we add a depolarizing error channel after each individual gate. In terms of the quantum channel, it is quantified as

$$\begin{aligned}\mathcal{E}_{A_0}(\varrho) = &\left(1 - \frac{3}{4}r\right)\varrho + \frac{r}{4}\left((X_{A_0} \otimes I_{A_1})\,\varrho\,(X_{A_0} \otimes I_{A_1})\right.\\ &\left. + (Y_{A_0} \otimes I_{A_1})\,\varrho\,(Y_{A_0} \otimes I_{A_1}) + (Z_{A_0} \otimes I_{A_1})\,\varrho\,(Z_{A_0} \otimes I_{A_1})\right),\\ \mathcal{E}_{A_0,A_1}(\varrho) = &(1-r)\varrho + r\frac{I_{A_0,A_1}}{4}\end{aligned} \tag{159}$$

where r is the error rate. At the same time, the quantum circuit is subject to drift error according to Equations (157) and (158).

In Figure 11, we numerically test the accuracy of estimating the circuit fidelity using the Fourier space data according to the estimator in Equation (156). The reference value of the circuit fidelity is computed from the digital error model (DEM) [4] with

$$\alpha_{\mathrm{DEM}} := (1-r)^{n_{\mathrm{gates}}} \approx (1-r)^{2d+5} + \mathcal{O}(r). \tag{160}$$

Here, n_{gates} stands for the number of total gates in the quantum circuit. Because of the additional phase gate used in the Bell-state preparation, the quantum circuit for computing p_Y uses $n_{\mathrm{gates}} = 2d + 6$ gates while that for p_X uses $n_{\mathrm{gates}} = 2d + 5$ gates. This ambiguity in a gate makes the left-hand side approximate the circuit fidelity up to $\mathcal{O}(r)$. In Figure 11, the performance of the circuit fidelity estimation is quantified by the deviation $|\hat{\alpha} - \alpha_{\mathrm{DEM}}|$. As the circuit depth of QSPE increases, it turns out that the deviation decreases to ~ 0.001, which is equal to the error rate r. The decreasing deviation is due to the improvement of the SNR when increasing the circuit depth. Furthermore, the plateau near 0.001 is due to the ambiguity discussed in the reference α_{DEM}. In the left panel, we turn off the time-dependent drift error and the quantum circuit is only subject to Monte Carlo sampling error and depolarizing error. However, the performance of the circuit fidelity estimation does not differ significantly after turning on the time-dependent drift error. The numerical results suggest that the depolarizing error can be inferred with considerable accuracy even in the presence of more complex time-dependent errors.

In Figures 12 and 13, we test our proposed metrology scheme in the presence of Monte Carlo sampling error, depolarizing error, and time-dependent error. Although the system is subjected to realistic errors, the numerical results suggest that the QSPE estimators show some robustness against errors, and they can give reasonable estimation results with one or two correct digits. Furthermore, the accuracy of φ-estimation is also not fully contaminated by the time-dependent error on it. The improvement due to the peak fitting becomes less significant under realistic errors because the structure of the highest peak is heavily distorted in the presence of realistic errors. More interestingly, the numerical results show the accuracy of θ-estimation does not decay and even increases after some d^*. This transition is due to a tradeoff. When d becomes larger, the inference is expected to be more accurate because the gate parameters are more amplified. However, in the presence of realistic error, the FsimGate is subjected to both time-independent errors and time-dependent drift errors. A quantum circuit with more FsimGate s violates the model derived from the noiseless setting more. The competition between these two opposite effects makes the estimation error attain

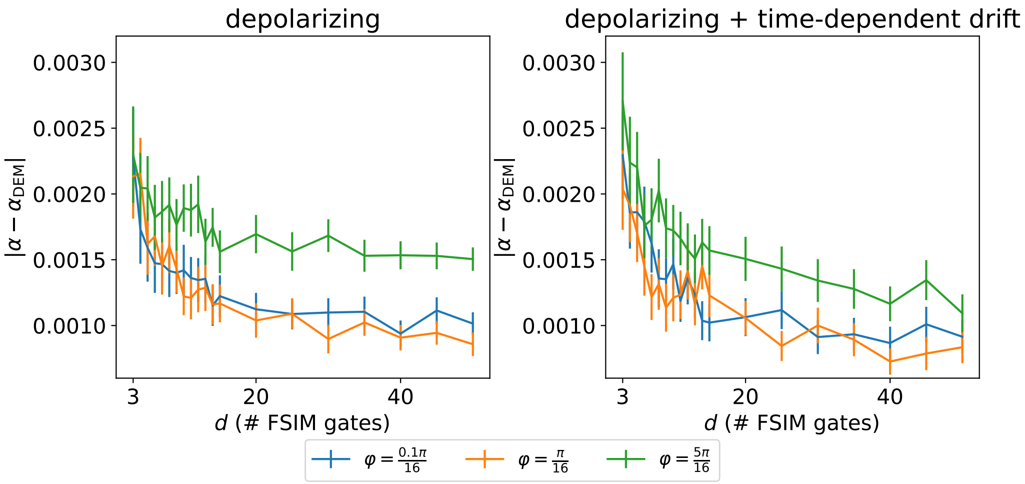

Supplementary Figure 11. Estimating circuit fidelity using QSPE. The reference value α_{DEM} is the circuit fidelity estimated from the digital error model. The sources of noise in the numerical experiments are Monte Carlo sampling error, depolarizing error and drift error. The depolarizing error rate is set to $r = 1 \times 10^{-3}$ and the number of measurement samples is set to $M = 1 \times 10^5$. The parameters of FsimGate are set to $\theta = 1 \times 10^{-3}$ and $\chi = 5\pi/32$. The error bar of each point stands for the confidence interval derived from 96 independent repetitions.

some minimum at d^*. This observation also suggests that in the experimental deployment, one can consider using a moderate d with respect to the tradeoff.

In Figure 13, we perform the numerical simulation with variable swap angle and number of measurement samples. Similar to the case of Monte Carlo sampling error, the estimation results are less accurate when θ is small because of the insufficient SNR. The numerical results indicate that the estimation accuracy cannot be further improved after the number of measurement samples is greater than some M^*. That is because increasing M can only mitigate Monte Carlo sampling error. When M is large enough, the sources of errors are dominated by depolarizing error and time-dependent drift error, which cannot be sufficiently mitigated by large M. Combing with the discussion on d^*, the numerical results suggest that the experimental deployment does not require large d and M, and using a moderate choice of d^* and M^* suffices to get some accurate estimation.

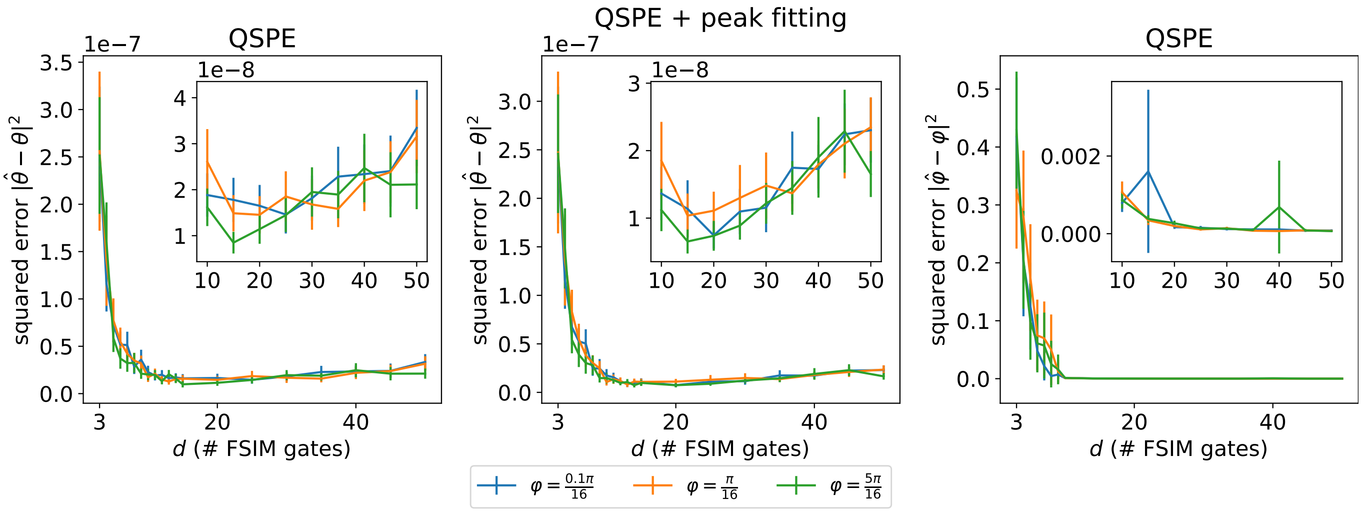

Supplementary Figure 12. Accuracy of estimators as a function of the number of FsimGate s. The sources of noise in the numerical experiments are Monte Carlo sampling error, depolarizing error and time-dependent drift error. The depolarizing error rate is set to $r = 1 \times 10^{-3}$ and the number of measurement samples is set to $M = 1 \times 10^5$. The swap angle is set to $\theta = 1 \times 10^{-3}$ and the phase parameter is set to $\chi = 5\pi/32$. The error bar of each point stands for the confidence interval derived from 96 independent repetitions.

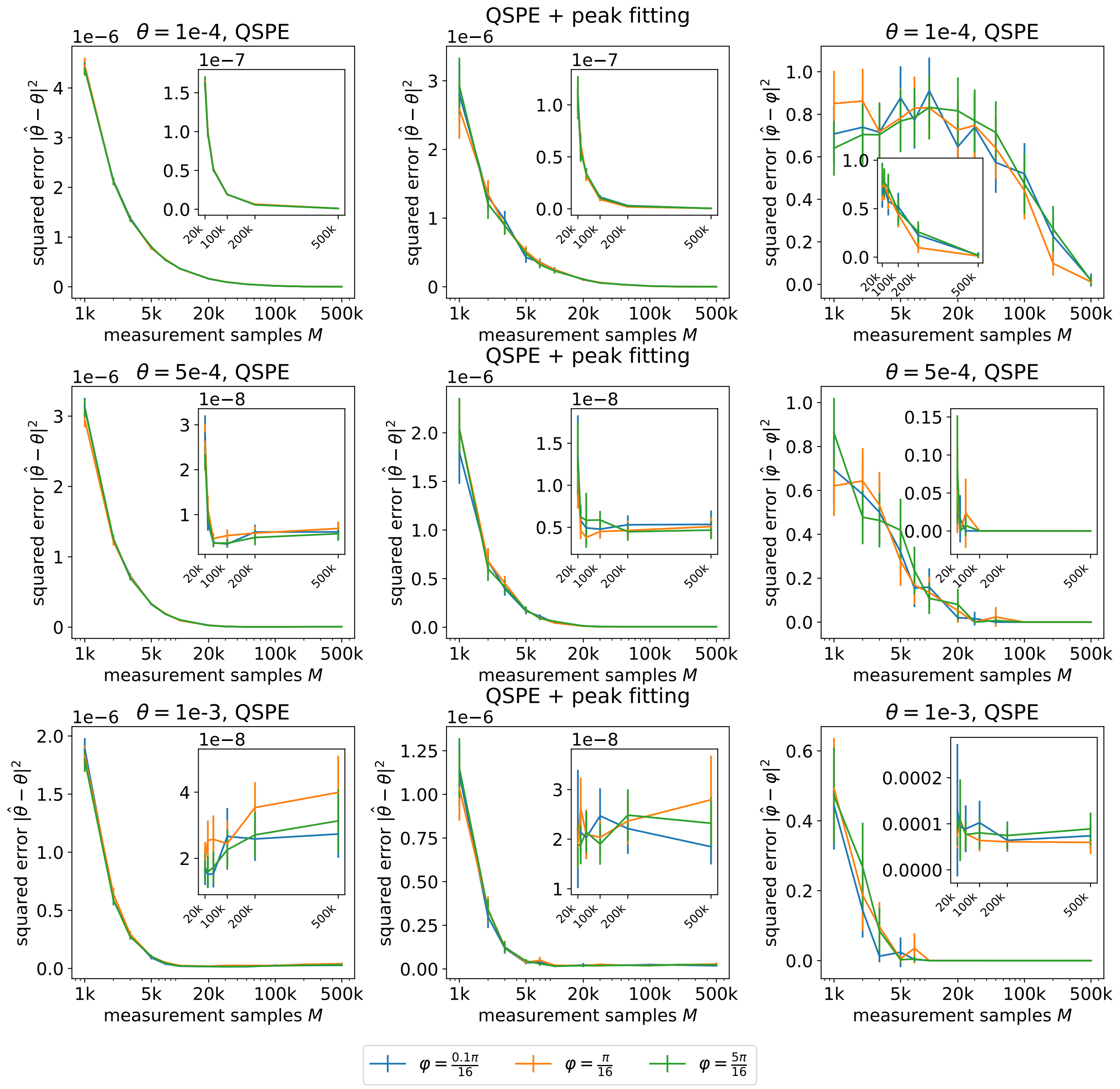

Supplementary Figure 13. Accuracy of estimators as a function of the number of measurement samples. The sources of noise in the numerical experiments are Monte Carlo sampling error, depolarizing error and time-dependent drift error. The depolarizing error rate is set to $r = 1 \times 10^{-3}$. The circuit degree is set to $d = 50$ and the FsimGate phase parameter is set to $\chi = 5\pi/32$.The error bar of each point stands for the confidence interval derived from 96 independent repetitions.

D. Readout error

The readout error is modeled by a stochastic matrix whose entry is interpreted as a conditional probability. This matrix is referred to as the confusion matrix in the readout. For a two-qubit system, it takes the form

$$R := [\mathbb{P}(\text{binary}(j)|\text{binary}(i))]_{i,j=0}^{3} = \begin{pmatrix} \mathbb{P}(00|00) & \mathbb{P}(01|00) & \mathbb{P}(10|00) & \mathbb{P}(11|00) \\ \mathbb{P}(00|01) & \mathbb{P}(01|01) & \mathbb{P}(10|01) & \mathbb{P}(11|01) \\ \mathbb{P}(00|10) & \mathbb{P}(01|10) & \mathbb{P}(10|10) & \mathbb{P}(11|10) \\ \mathbb{P}(00|11) & \mathbb{P}(01|11) & \mathbb{P}(10|11) & \mathbb{P}(11|11) \end{pmatrix} \tag{161}$$

where $\mathbb{P}(\text{binary}(j)|\text{binary}(j))$ is the conditional probability of measuring the qubits with the bit-string binary(j) given that the quantum state is $|\text{binary}(i)\rangle$. The sum of each row of the confusion matrix is equal to one due to the normalization of probability. The confusion matrix can be determined by performing additional quantum experiments in which $I \otimes I$, $I \otimes X$, $X \otimes I$, and $X \otimes X$ are measured to determine each row, respectively. If the probability vector from the measurement with readout error is $\mathbf{q}^{\text{exp}} = (q^{\text{exp}}(00), q^{\text{exp}}(01), q^{\text{exp}}(10), q^{\text{exp}}(11))^\top$, the probability vector after correcting the readout error is given by inverting the confusion matrix

$$\mathbf{p}^{\text{exp}} = (p^{\text{exp}}(00), p^{\text{exp}}(01), p^{\text{exp}}(10), p^{\text{exp}}(11))^\top = \left(R^\top\right)^{-1} \mathbf{q}^{\text{exp}}. \tag{162}$$

In practice, the confusion matrix is determined by finite measurement samples which could introduce error to the confusion matrix due to the statistical fluctuation. We analyze the error and its effect in Theorem 19. As a consequence, the theorem indicates a minimal requirement on the measurement sample size so that the readout error can be accurately corrected.

Theorem 19. *Let $\mathbf{p}_{\text{fs}}^{\text{exp}}$ be the probability vector computed by inverting the confusion matrix estimated by finite samples. To achieve the bounded error $\|\mathbf{p}^{\text{exp}} - \mathbf{p}_{\text{fs}}^{\text{exp}}\|_2 \le \epsilon$ with confidence level $1-\alpha$, it suffices to set the number of measurement samples in each experiment determining the confusion matrix as*

$$M_{\text{cmt}} = \left\lceil \frac{2\kappa^2(\kappa+\epsilon)^2 \ln(32/\alpha)}{\epsilon^2} \right\rceil \tag{163}$$

where

$$\kappa = \max_{i=0,\cdots,3} \frac{1}{2R_{ii}-1}. \tag{164}$$

Proof. In each experiment given the exact outcome $u \in \{0,1\}^2$ without readout error and exact measurement probability vector $\mathbf{p}^{(u)} := (p(00|u), p(01|u), p(10|u), p(11|u))$ taking readout error into account, the number of measurement outcomes corresponding to each bit-string is multinomial distributed

$$\mathbf{k}^{(u)} := (k(00|u), k(01|u), k(10|u), k(11|u)) \sim \text{Multinomial}(M_{\text{cmt}}, \mathbf{p}^{(u)}) \tag{165}$$

where $k(s|u) := \#(\text{outcome is } s \text{ in } M_{\text{cmt}} \text{ samples})$. The bit-string frequency

$$\mathbf{q}^{(u)} = (q(00|u), q(01|u), q(10|u), q(11|u)) := \left(\frac{k(00|u)}{M_{\text{cmt}}}, \frac{k(01|u)}{M_{\text{cmt}}}, \frac{k(10|u)}{M_{\text{cmt}}}, \frac{k(11|u)}{M_{\text{cmt}}}\right) \tag{166}$$

is therefore an estimate to the measurement probability since $\mathbb{E}\left(\mathbf{q}^{(u)}\right) = \mathbf{p}^{(u)}$. However, the statistical fluctuation makes the estimate deviate the exact probability. Applying Hoeffding's inequality, we have

$$\mathbb{P}\left(|q(s|u) - p(s|u)| > \frac{\widetilde{\epsilon}}{4}\right) = \mathbb{P}\left(|k(s|u) - M_{\text{cmt}} p(s|u)| > \frac{\widetilde{\epsilon} M_{\text{cmt}}}{4}\right) \le 2e^{-\frac{\widetilde{\epsilon}^2 M_{\text{cmt}}}{8}}. \tag{167}$$

Let the confusion matrix determined by finite samples be R_{fs} where $(R_{\text{fs}})_{ij} = q\left(\text{binary}(j)|\text{binary}(i)\right)$ and the subscript "fs" abbreviates "finite sample". Then, the deviation can be bounded as

$$\begin{aligned} &\mathbb{P}\left(\|R_{\text{fs}} - R\|_2 > \widetilde{\epsilon}\right) \le \mathbb{P}\left(\|R_{\text{fs}} - R\|_F > \widetilde{\epsilon}\right) = \mathbb{P}\left(\sum_{s,u\in\{0,1\}^2} |q(s|u) - p(s|u)|^2 > \widetilde{\epsilon}^2\right) \\ &\le \mathbb{P}\left(\bigcup_{s,u\in\{0,1\}^2} \left\{|q(s|u) - p(s|u)| > \frac{\widetilde{\epsilon}}{4}\right\}\right) \le \sum_{s,u\in\{0,1\}^2} \mathbb{P}\left(|q(s|u) - p(s|u)| > \frac{\widetilde{\epsilon}}{4}\right) \\ &\le 32 e^{-\frac{\widetilde{\epsilon}^2 M_{\text{cmt}}}{8}}. \end{aligned} \tag{168}$$

Therefore, to achieve $\|R_{\rm fs}-R\|_2 \le \widetilde{\epsilon}$ with confidence level $1-\alpha$, it suffices to set the number of measurement samples in each experiment as

$$M_{\rm cmt} = \left\lceil \frac{8\ln(32/\alpha)}{\widetilde{\epsilon}^2} \right\rceil . \tag{169}$$

Expanding the matrix inverse in terms of power series and denoting $\Delta_{\rm fs} := R_{\rm fs} - R$ for convenience, we have

$$R_{\rm fs}^{-1} = (R+\Delta_{\rm fs})^{-1} = R^{-1}\left(I+\Delta_{\rm fs}R^{-1}\right)^{-1} = R^{-1} + \sum_{j=1}^{\infty} R^{-1}\left(\Delta_{\rm fs}R^{-1}\right)^j . \tag{170}$$

Furthermore, we get

$$\left\|R_{\rm fs}^{-1}-R^{-1}\right\|_2 \le \left\|R^{-1}\right\|_2 \sum_{j=1}^{\infty}\left\|\Delta_{\rm fs}R^{-1}\right\|_2^j \le \frac{\|\Delta_{\rm fs}\|_2\left\|R^{-1}\right\|_2^2}{1-\|\Delta_{\rm fs}\|_2\|R^{-1}\|_2}. \tag{171}$$

Note that $\left\|R^{-1}\right\|_2 = \lambda_{\min}^{-1}(R)$. To proceed, we have to lower bound the smallest eigenvalue of the confusion matrix. Note that all eigenvalues of the confusion matrix are real as a property of the stochastic matrix. Applying the Gershgorin circle theorem, all eigenvalues of the confusion matrix are contained in the union of intervals

$$\bigcup_{i=0}^{3}\left[R_{ii}-\sum_{j\neq i}R_{ij}, R_{ii}+\sum_{j\neq i}R_{ij}\right] . \tag{172}$$

Consequentially, the smallest eigenvalue of the confusion matrix is lower bounded

$$\lambda_{\min}(R) \ge \min_{i=0,\cdots,3}\left(R_{ii}-\sum_{j\neq i}R_{ij}\right) = \min_{i=0,\cdots,3}(2R_{ii}-1) =: \kappa^{-1}. \tag{173}$$

Thus, by properly choosing the number of measurement samples, with confidence level $1-\alpha$, we can bound the inverse confusion matrix as

$$\left\|R_{\rm fs}^{-1}-R^{-1}\right\|_2 \le \frac{\widetilde{\epsilon}\kappa^2}{1-\widetilde{\epsilon}\kappa}. \tag{174}$$

When computing the probability vector by inverting the confusion matrix determined by finite measurement samples, the error is bounded as

$$\|\mathbf{p}^{\rm exp}-\mathbf{p}_{\rm fs}^{\rm exp}\|_2 \le \left\|R_{\rm fs}^{-1}-R^{-1}\right\|_2\|\mathbf{q}^{\rm exp}\|_2 \le \left\|R_{\rm fs}^{-1}-R^{-1}\right\|_2\|\mathbf{q}^{\rm exp}\|_1 \le \frac{\widetilde{\epsilon}\kappa^2}{1-\widetilde{\epsilon}\kappa}. \tag{175}$$

Let

$$\frac{\widetilde{\epsilon}\kappa^2}{1-\widetilde{\epsilon}\kappa} = \epsilon \Rightarrow \widetilde{\epsilon} = \frac{\epsilon}{\kappa(\kappa+\epsilon)} \tag{176}$$

Thus, to achieve the bounded error $\|\mathbf{p}^{\rm exp}-\mathbf{p}_{\rm fs}^{\rm exp}\|_2 \le \epsilon$ with confidence level $1-\alpha$, it suffices to set the number of measurement samples in each experiment determining the confusion matrix as

$$M_{\rm cmt} = \left\lceil \frac{8\kappa^2(\kappa+\epsilon)^2\ln(32/\alpha)}{\epsilon^2} \right\rceil . \tag{177}$$

The proof is completed. □

E. Initial state preparation error

In this subsection, we analyze the effect of the error in the initialization of Bell states in the quantum circuits. One case is that the error brings the initial state out of the subspace $\mathrm{span}\{|0_\ell\rangle, |1_\ell\rangle\}$. Because the rest of the circuit

preserves that two-dimensional subspace, such error can be mitigated by post-selecting the measurement outcomes in the two-dimensional subspace. Hence, in the rest of this subsection, we focus on the initial state error within the two-dimensional subspace.

The initial state error is modeled by a unitary rotation $E_\eta := e^{-\mathrm{i}\eta K}$ where $\eta \in \mathbb{R}$ is the error magnitude and K is the normalized Hermitian generator of the error with $\|K\|_2 = 1$. Suppose the ideal Bell state is $|\beta\rangle$ with $\beta \in \{+, \mathrm{i}\}$, the experimentally prepared initial state is $|\beta'\rangle := E_\eta |\beta\rangle \approx |\beta\rangle - \mathrm{i}\eta K |\beta\rangle + \mathcal{O}(\eta^2)$. The experimental measurement probability is then

$$\begin{aligned} p'_\beta &= \left|\langle 0_\ell|\mathcal{U}^{(d)}(\omega;\theta,\varphi,\chi)|\beta'\rangle\right|^2 \approx \left|\langle 0_\ell|\mathcal{U}^{(d)}(\omega;\theta,\varphi,\chi)|\beta\rangle - \mathrm{i}\eta\langle 0_\ell|\mathcal{U}^{(d)}(\omega;\theta,\varphi,\chi)K|\beta\rangle\right|^2 + \mathcal{O}(\eta^2) \\ &\approx p_\beta\left(1 + \mathcal{O}(p_\beta^{-1/2}\eta\langle 0_\ell|\mathcal{U}^{(d)}(\omega;\theta,\varphi,\chi)K|\beta\rangle)\right) + \mathcal{O}(\eta^2) \\ &\approx p_\beta + \mathcal{O}(\eta\langle 0_\ell|\mathcal{U}^{(d)}(\omega;\theta,\varphi,\chi)K|\beta\rangle) + \mathcal{O}(\eta^2 + d\theta\eta) \end{aligned} \tag{178}$$

Here, the second and the third lines use the conclusion that $\langle 0_\ell|\mathcal{U}^{(d)}(\omega;\theta,\varphi,\chi)|\beta\rangle = \mathcal{O}(\sqrt{p_\beta}) = \mathcal{O}(1) + \mathcal{O}(d\theta)$ when $d\theta \ll 1$. Note that Equations (140) and (142) indicate that

$$\mathcal{O}(\mathcal{U}^{(d)}(\omega;\theta,\varphi,\chi)) = \mathcal{O}(I + \mathrm{i}\theta X U_{d-1}(\cos(\omega-\varphi))) + \mathcal{O}((d\theta)^2). \tag{179}$$

Note that the identity matrix in the expansion only contributes to the constant term, which we explicitly annotate in the following result:

$$p'_\beta \approx p_\beta + \mathrm{const} \times \eta + \mathrm{const} \times \eta\theta U_{d-1}(\omega - \varphi) + \mathcal{O}(\eta^2 + d\theta\eta). \tag{180}$$

We remark that the constant-shift term const $\times\, \eta$ only affects the zeroth Fourier mode, which can be dropped by post-selecting Fourier modes of positive indices. This perturbative analysis implies that when subjected to initial state error with magnitude η, each Fourier component has an additive error magnitude $\mathcal{O}(\eta\theta)$. Thus, the initial state error contributes to a relative error in the estimation, $|\hat{\theta} - \theta|/\theta = \mathcal{O}(\eta)$.

The perturbative analysis above gives intuition in understanding the initial state error. However, the derivation relies on several approximations that may not cover all cases. In the following text, we perform numerical analysis to have a more comprehensive understanding of the estimation error induced by initial state error. We turn off all other noises including sampling error to focus on the effect of initial state error. We perform three different strategies to add initial state error to the system.

1. "identical": The initial state error $E_\eta = \exp\big(-\mathrm{i}\eta(X+Y)/\sqrt{2}\big)$ is identical in all experiments for any $\beta \in \{+, \mathrm{i}\}$ and any ω.
2. "fixed": The initial state error E_η only depends on the initial state to be prepared. It is fixed in all experiments for any ω but takes two possible values $E_{\eta,+} = \exp\big(-\mathrm{i}\eta(X+Y)/\sqrt{2}\big)$ and $E_{\eta,\mathrm{i}} = \exp\big(-\mathrm{i}\eta(2X+Y)/\sqrt{5}\big)$.
3. "random": The initial state error is drawn randomly in each experiment for each $\beta \in \{+, \mathrm{i}\}$ and ω, namely, $E_\eta = \exp(-\mathrm{i}\eta K)$ and K is a random normalized Hermitian error generator.

The numerical results are given in Figure 14. These results suggest that the error scaling depends weakly on the strategy of adding initial state error. The study in Figure 14(a) suggests that the estimation error is linearly additive in the initial state error η, which agrees with our perturbative analysis. Moreover, we see that when $\theta = 10^{-3}$ and $\eta = 10^{-3}$, the induced estimation error of θ is around 10^{-6} and that of φ is around 10^{-3} which are negligible compared to their own magnitudes. It is also worth noting that the plateau around 10^{-7} of θ estimation error (left panel in Figure 14(a)) is because the approximation error derived in Section Supplementary Information 3 dominates the estimation error. In Figure 14(b), we study the error scaling in a wide range of θ values. We see that the function form is nontrivial across different θ values. When θ is around 10^{-3}, the θ estimation error scales quadratically in θ, which might be due to some complex cancellation in Fourier transformation. The φ estimation error scales reciprocally in θ, because the signal-to-noise ratio of the complex phase increases as θ gets large. It agrees with the analysis in Section Supplementary Information 3. Moreover, Figure 14(c) shows that the spam error can be mitigated by increasing the circuit depth as the estimations error decays as d^{-1} and d^{-2} for θ and φ respectively. Theoretically understanding these error scalings will be part of our future work.

Supplementary Information 8. Computing the polynomial representation on a special set of points

Lemma 20. *Let $d = 2^j$ for some $j = 0, 1, 2, \cdots$. Then*

$$P_\omega^{(d)}(x) = e^{\mathrm{i}\omega}\left(\cos(d\sigma) + \mathrm{i}\frac{\sin(d\sigma)}{\sin\sigma}(\sin\omega)\,x\right) \text{ and } Q_\omega^{(d)}(x) = \frac{\sin(d\sigma)}{\sin\sigma} \tag{181}$$

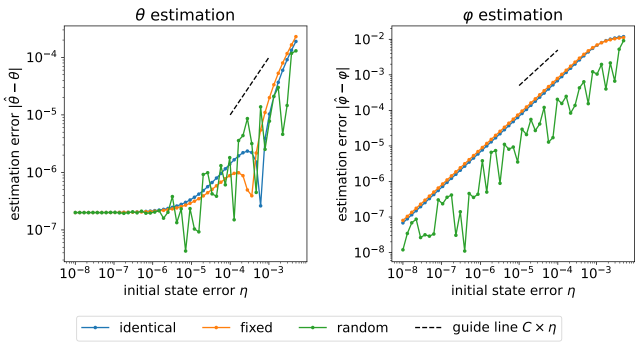

(a) Estimation error as a function of initial state error η ($\theta = 10^{-3}, d = 20$)

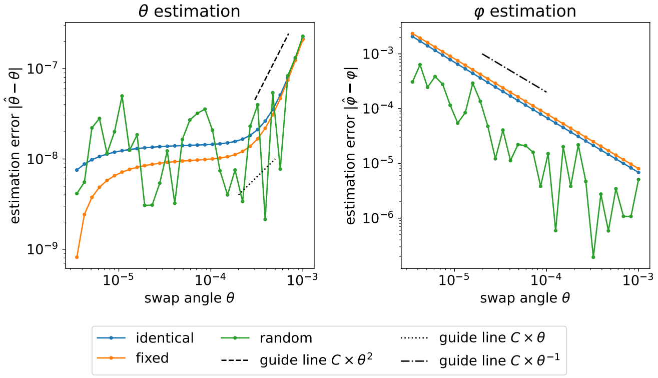

(b) Estimation error as a function of swap angle θ ($\eta = 10^{-6}, d = 20$)

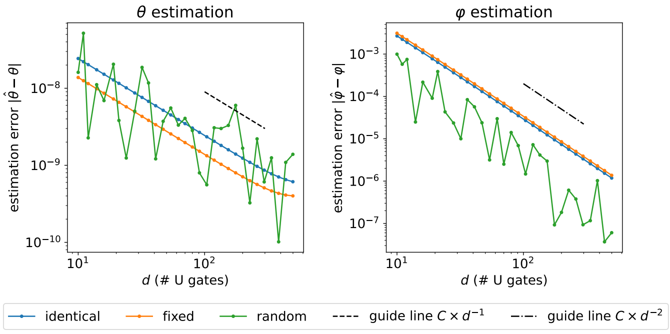

(c) Estimation error as a function of depth parameter d ($\theta = 10^{-5}, \eta = 10^{-6}$)

Supplementary Figure 14. Estimation error induced by initial state error. We set $\varphi = 3\pi/16$ and $\chi = 5\pi/32$ for all experiments.

where $\sigma = \arccos\left((\cos\omega)\, x\right)$.

Proof. A system of recurrence relations can be established by inserting the resolution of identity in the matrix multiplication:

$$\begin{aligned}
\mathrm{i}\sqrt{1-x^2}Q^{(d)}(x) &= \langle 0|\, U^{(d)}(\omega,\theta)\,|1\rangle = \langle 0|\, U^{(d/2)}(\omega,\theta) e^{-\mathrm{i}\omega Z}\left(|0\rangle\langle 0| + |1\rangle\langle 1|\right) U^{(d/2)}(\omega,\theta)\,|1\rangle \\
&= e^{-\mathrm{i}\omega} P_\omega^{(d/2)}(x)\mathrm{i}\sqrt{1-x^2}Q_\omega^{(d/2)}(x) + e^{\mathrm{i}\omega}\mathrm{i}\sqrt{1-x^2}Q_\omega^{(d/2)}(x) P_\omega^{(d/2)*}(x) \\
&= \mathrm{i}\sqrt{1-x^2}Q_\omega^{(d/2)}(x) 2\mathrm{Re}\left(e^{-\mathrm{i}\omega} P_\omega^{(d/2)}(x)\right) \\
\Rightarrow Q_\omega^{(d)}(x) &= 2Q_\omega^{(d/2)}(x)\mathrm{Re}\left(e^{-\mathrm{i}\omega} P_\omega^{(d/2)}(x)\right),
\end{aligned} \tag{182}$$

and

$$\begin{aligned}
P_\omega^{(d)}(x) &= \langle 0|\, U^{(d)}(\omega,\theta)\,|0\rangle = \langle 0|\, U^{(d/2)}(\omega,\theta) e^{-\mathrm{i}\omega Z}\left(|0\rangle\langle 0| + |1\rangle\langle 1|\right) U^{(d/2)}(\omega,\theta)\,|0\rangle \\
&= e^{-\mathrm{i}\omega}\left(P_\omega^{(d/2)}(x)\right)^2 - e^{\mathrm{i}\omega}(1-x^2)\left(Q_\omega^{(d/2)}(x)\right)^2 \\
&\overset{(\star)}{=} -e^{\mathrm{i}\omega} + 2P_\omega^{(d/2)}(x)\mathrm{Re}\left(e^{-\mathrm{i}\omega} P_\omega^{(d/2)}(x)\right) \\
\Rightarrow \mathrm{Re}\left(e^{-\mathrm{i}\omega} P_\omega^{(d)}(x)\right) &= -1 + 2\mathrm{Re}^2\left(e^{-\mathrm{i}\omega} P_\omega^{(d/2)}(x)\right), \\
\text{and } \mathrm{Im}\left(e^{-\mathrm{i}\omega} P_\omega^{(d)}(x)\right) &= 2\mathrm{Im}\left(e^{-\mathrm{i}\omega} P_\omega^{(d/2)}(x)\right)\mathrm{Re}\left(e^{-\mathrm{i}\omega} P_\omega^{(d/2)}(x)\right).
\end{aligned} \tag{183}$$

Here, equation $(\star)$ uses the special unitarity of $U^{(d/2)}(\omega,\theta)$ which yields $P_\omega^{(d/2)}(x)P_\omega^{(d/2)*}(x) + (1-x^2)\left(Q_\omega^{(d/2)}(x)\right)^2 = 1$ by taking determinant. We will first solve the nonlinear recurrence relation for $\mathrm{Re}\left(e^{-\mathrm{i}\omega}P_\omega^{(d)}\right)$ in Equation (183). Note

that the second-order Chebyshev polynomial of the first kind is $T_2(x) = 2x^2 - 1$. Then,

$$\mathrm{Re}\left(e^{-\mathrm{i}\omega}P_\omega^{(d)}(x)\right) = T_2\left(\mathrm{Re}\left(e^{-\mathrm{i}\omega}P_\omega^{(d/2)}(x)\right)\right) = \cdots = \underbrace{T_2 \circ \cdots \circ T_2}_{\log_2(d)}\left(\mathrm{Re}\left(e^{-\mathrm{i}\omega}P_\omega^{(1)}(x)\right)\right) \tag{184}$$

Using the composition identity of the Chebyshev polynomials $T_n \circ T_m = T_{nm}$, we have $\underbrace{T_2 \circ \cdots \circ T_2}_{\log_2(d)} = T_d$. On the other hand, when $d = 1$, we have

$$\begin{aligned} U^{(1)}(\omega, \arccos(x)) &= e^{\mathrm{i}\omega Z} e^{\mathrm{i}\arccos(x) X} e^{\mathrm{i}\omega Z} = \begin{pmatrix} e^{2\mathrm{i}\omega}x & \mathrm{i}\sqrt{1-x^2} \\ \mathrm{i}\sqrt{1-x^2} & e^{-2\mathrm{i}\omega}x \end{pmatrix} \\ \Rightarrow e^{-\mathrm{i}\omega}P_\omega^{(1)}(x) &= e^{\mathrm{i}\omega}x,\ Q_\omega^{(1)}(x) = 1. \end{aligned} \tag{185}$$

Therefore

$$\mathrm{Re}\left(e^{-\mathrm{i}\omega}P_\omega^{(d)}(x)\right) = T_d\left((\cos\omega)\, x\right). \tag{186}$$

Furthermore, $Q_\omega^{(d)}$ and $\mathrm{Im}\left(e^{-\mathrm{i}\omega}P_\omega^{(d)}\right)$ can be determined from the recurrence relation in Equations (182) and (183)

$$Q_\omega^{(d)}(x) = d\prod_{j=0}^{\log_2(d)-1} T_{2^j}\left((\cos\omega)\, x\right),\ \mathrm{Im}\left(e^{-\mathrm{i}\omega}P_\omega^{(d)}(x)\right) = Q_\omega^{(d)}(x)\,(\sin\omega)\, x. \tag{187}$$

For convenience, let $\cos\sigma := (\cos\omega)\, x = \cos\omega\cos\theta$. Then

$$\begin{aligned} Q_\omega^{(d)}(x)\sin\sigma &= \left(\frac{d}{2}\prod_{j=1}^{\log_2(d)-1}\right) 2\cos\sigma\sin\sigma = \left(\frac{d}{4}\prod_{j=2}^{\log_2(d)-1}\right) 2\cos(2\sigma)\sin(2\sigma) \\ &= \cdots = 2\cos\left(\frac{d}{2}\sigma\right)\sin\left(\frac{d}{2}\sigma\right) = \sin(d\sigma). \end{aligned} \tag{188}$$

Therefore

$$P_\omega^{(d)}(x) = e^{\mathrm{i}\omega}\left(\cos(d\sigma) + \mathrm{i}\frac{\sin(d\sigma)}{\sin\sigma}(\sin\omega)\, x\right),\ \text{and } Q_\omega^{(d)}(x) = \frac{\sin(d\sigma)}{\sin\sigma}. \tag{189}$$

□

Supplementary Information 9. Analysis of periodic calibration: variance lower bound and shortcomings

A. Overview of the methodology of periodic calibration

In this subsection, we provide an overview of periodic calibration, also known as Floquet calibration, which is proposed in [2, Appendix C] and [17, Appendix A]. Periodic calibration is a generalization of the robust single-qubit gate calibration [12] to entangling gates. The main component of the quantum circuit for performing periodic calibration is equivalent to the periodic part used in our method (see the shaded part in Figure 1 in the main text). However, instead of the initialization in terms of Bell states, periodic calibration measures the transition probability between tensor product states $|01\rangle$ and $|10\rangle$, namely, between logical quantum state $|0_\ell\rangle$ and $|1_\ell\rangle$. The exact parametric expression of this probability can be derived from the results presented in Section Supplementary Information 3, which is consistent with that in literature (upon convention difference):

$$P_{\mathrm{pc}}(\theta, \varphi, \omega, d) = \sin^2(\theta)\frac{\sin^2(d\sigma)}{\sin^2(\sigma)} = \sin^2(\theta)U_{d-1}^2(\cos(\sigma)) \text{ where } \sigma = \arccos(\cos(\theta)\cos(\omega - \varphi)). \tag{190}$$

Given the parametric expression, periodic calibration is performed by minimizing the distance metric between the parametric ansatz and the experimentally measured probabilities. To improve the efficiency, the depth parameter d is chosen to be logarithmically spaced, namely, $d = 1, 2, 4, 8, \cdots$. In practice, multiple modulation angles ω can be chosen. Yet, for simplicity, and due to the linear additivity of the Fisher information gained from multiple ω values, we focus on a single variable value of ω in our analysis. Remarkably, we prove that for each circuit depth, the Fisher information is maximized when $\omega = \varphi$, which is referred to as phase-matching condition. Consequently, the simplified choice of fixed ω value in the analysis will indeed capture the optimality of the estimation.

B. Optimality analysis using Fisher information and Cramér-Rao bound

According to the procedure outlined in the previous section, we can compute the Fisher information as follows. Suppose $d = 2^L$ is the maximum depth of the experiment, and M is the number of measurement samples in each experiment. For notational simplicity, let the parameter vector be $\Xi = (\xi_1 := \theta, \xi_2 := \varphi)$. Then, the Fisher information matrix is expressed as:

$$I_{k,k'}(\Xi;\omega,d) = \sum_{j=0}^{\log_2(d)} \frac{M}{P_{\rm pc}(\theta,\varphi,\omega,2^j)(1-P_{\rm pc}(\theta,\varphi,\omega,2^j))} \frac{\partial P_{\rm pc}(\theta,\varphi,\omega,2^j)}{\partial \xi_k} \frac{\partial P_{\rm pc}(\theta,\varphi,\omega,2^j)}{\partial \xi_{k'}}. \tag{191}$$

Here, the relevant derivatives are

$$\begin{aligned} \frac{\partial P_{\rm pc}(\theta,\varphi,\omega,d)}{\partial\theta} &= 2\sin(\theta)\cos(\theta)U_{d-1}^2(\cos(\sigma)) - 2\sin^3(\theta)\cos(\omega-\varphi)U_{d-1}(\cos(\sigma))U'_{d-1}(\cos(\sigma)), \\ \frac{\partial P_{\rm pc}(\theta,\varphi,\omega,d)}{\partial\varphi} &= 2\sin^2(\theta)\cos(\theta)\sin(\omega-\varphi)U_{d-1}(\cos(\sigma))U'_{d-1}(\cos(\sigma)), \end{aligned} \tag{192}$$

and

$$U'_{d-1}(\cos(\sigma)) = -\frac{d\cos(d\sigma) - \cos(\sigma)U_{d-1}(\cos(\sigma))}{\sin^2(\sigma)}.$$

Using the explicit expressions above, the Fisher information matrix can be numerically evaluated. The estimation variances are lower bounded by the diagonal elements of the inverse Fisher information matrix according to Cramér-Rao bound. For simplicity, we absorb the modulation angle ω into the definition of φ angle, namely, setting $\omega = 0$ and φ variable. Hence, the phase-matching condition is equivalent to $\varphi = 0$ ($\omega = \varphi$ in the original setting).

When the phase-matching condition is satisfied, the CRLB can be exactly derived. Note that $P_{\rm pc}|_{\varphi=0,d=\ell} = \sin^2(\ell\theta)$. Then, for a fixed circuit depth ℓ with M measurement shots, the Fisher information is

$$J_\theta(\ell)|_{\varphi=0} = \frac{M}{\sin^2(\ell\theta)\cos^2(\ell\theta)}(2\ell\sin(\ell\theta)\cos(\ell\theta))^2 = 4M\ell^2.$$

As we derived in Equation (145) in Section Supplementary Information 6 D, the quantum Fisher information that a quantum circuit using ℓ U-gates can maximally contribute is $\mathfrak{F}_{\max} = 4M\ell^2$. Hence, the coincidence between the classical Fisher information with phase-matching condition and the maximal quantum Fisher information indicates that the optimality is attained at phase-matching because the Fisher information attains its maximum.

Under phase-matching conditions, the total Fisher information is

$$I_{1,1} = \sum_{j=0}^{\log_2(d)} \frac{M}{\sin^2(2^j\theta)\cos^2(2^j\theta)} 4\times 2^{2j}\sin^2(2^j\theta)\cos^2(2^j\theta) = 4M\sum_{j=0} 2^{2j} = \frac{4M(4d^2-1)}{3}.$$

Hence, the CRLB on the estimation variance of θ is

$$\mathrm{Var}(\theta)|_{\rm phase-matching} \geq I_{1,1}^{-1} = \frac{3}{4M(4d^2-1)} \approx \frac{3}{16Md^2}. \tag{193}$$

The numerical results are depicted in Figure 15. In Figure 15(a), it can be seen that the improvement in the estimation variance with increasingly large depth d is very limited when φ angle deviates from zero, namely, the phase-matching condition is violated. To understand the optimal variance scaling of periodic calibration as a function of the depth parameter d, we numerically depict the results in Figure 15(b). The numerical result indicates that the variance scales as $1/d^2$ when the phase-matching condition is satisfied. This is consistent with the exactly derived result in Equation (193). Yet when the phase-matching condition is violated, the variance decays as $1/d^2$ when the depth parameter is not too large ($d \lesssim 1/\varphi$). However, when d gets increasingly large, the variance is almost plateaued with very limited decay.

C. Violation of phase-matching condition implies exponentially worse estimation variance

To understand the importance of the phase-matching condition and the plateau of estimation variance, we provide an approximation to the Fisher information when the swap angle θ is small. For simplicity, we focus on the quantum

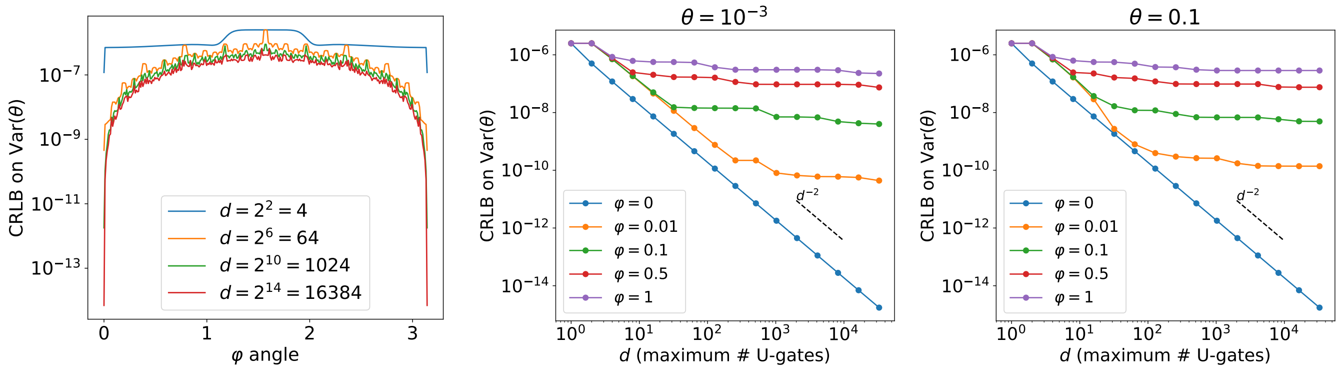

(a) CRLB as a function of φ angle ($\theta = 10^{-3}$)

(b) CRLB as a function of depth parameter d

Supplementary Figure 15. Cramér-Rao lower bound (CRLB) on the estimation variance of θ using periodic calibration. The number of measurement samples is set to $M = 1 \times 10^5$.

circuit with a fixed depth parameter d rather than a series of circuits with variable depths. It is referred to as J_θ below. We remark that the focus on the fixed-depth Fisher information does not lose generality due to the additivity of Fisher information.

Note that when θ is small, to the first order in θ, the Fisher information of θ is approximately

$$J_\theta \approx 4M \frac{\sin^2(d\varphi)}{\sin^2(\varphi)} + \mathcal{O}(d^4\theta^2). \tag{194}$$

Now, we provide an approximation to the expression above. Note that the extrema of $g(\varphi) := \sin(d\varphi)/\sin(\varphi)$ is attained when $d\tan(\varphi) = \tan(d\varphi)$ is satisfied. Let $\varphi_k^\star$ be the k-th solution to the equation involving tangent functions. They belong to the maxima of g^2, which are

$$g^2_{\max,k} = g^2(\varphi_k^\star) = \frac{\sin^2(d\varphi_k^\star)}{\sin^2(\varphi_k^\star)} = \frac{1 - \frac{1}{1+\tan^2(d\varphi_k^\star)}}{1 - \frac{1}{1+\tan^2(\varphi_k^\star)}} = \frac{1+\tan^2(\varphi_k^\star)}{d^{-2}+\tan^2(\varphi_k^\star)} \le \frac{1+(\varphi_k^\star)^2}{d^{-2}+(\varphi_k^\star)^2}.$$

By extrapolating this function to other points rather than maxima, we get a reasonably good approximation to the Fisher information:

$$J_\theta \lesssim \frac{1+\varphi^2}{d^{-2}+\varphi^2}. \tag{195}$$

Furthermore, when the violation of the phase-matching condition satisfies $|\varphi| \ge \pi/d$, the loss in the Fisher information compared to the maximum is

$$\frac{\max_{|\varphi|\ge\pi/d} J_\theta}{J_\theta|_{\text{phase-matching}}} \lesssim \frac{1}{d^2}\frac{1+(\pi/d)^2}{d^{-2}+(\pi/d)^2} = \frac{1+(\pi/d)^2}{1+\pi^2} \to \frac{1}{1+\pi^2} \approx 9.2\%. \tag{196}$$

Hence, though the phase-matching condition is slightly violated $|\varphi| \ge \pi/d$, which is common when d is large, more than 90% of the statistical power of θ-estimation is eliminated.

Furthermore, the plateaued estimation variance is justified. According to Equation (195), the total Fisher information is approximated as

$$I_{1,1} \lesssim 4M \sum_{j=0}^{\log_2(d)} \frac{1+\varphi^2}{4^{-j}+\varphi^2} \approx 4M \int_0^{\log_2(d)} \frac{1+\varphi^2}{4^{-x}+\varphi^2}\mathrm{d}x = 2M\frac{1+\varphi^2}{\varphi^2}\log_2\left(\frac{1+(d\varphi)^2}{1+\varphi^2}\right). \tag{197}$$

This result indicates that when the phase-matching condition is not satisfied, the Fisher information is exponentially diminished as $\Theta(\log(d))$ compared to the optimal $\Theta(d^2)$ Fisher information with the phase-matching condition.

To conclude, in Equation (196), we find that more than 90% of Fisher information is eliminated even though the phase-matching condition is slightly violated such that $|\varphi| \ge \pi/d$ is outside the principal peak. Moreover, in Equation (197), we derive that the majority of the violation of phase-matching condition, e.g. when φ is constantly

large, leads to an exponentially worse estimation variance rather than saturating Heisenberg limit. Due to the sensitivity of θ estimation to phase-matching conditions, our proposed QSPE method is superior because of the isolation of θ and φ estimations in Fourier space and its consequent robustness against realistic errors.

In Figure 16, we visualize the approximation analysis of Fisher information and CRLB. It can be seen that our approximation well fits the exact values and explains the exponentially worse scaling of θ-estimation variance when the phase-matching condition is violated.

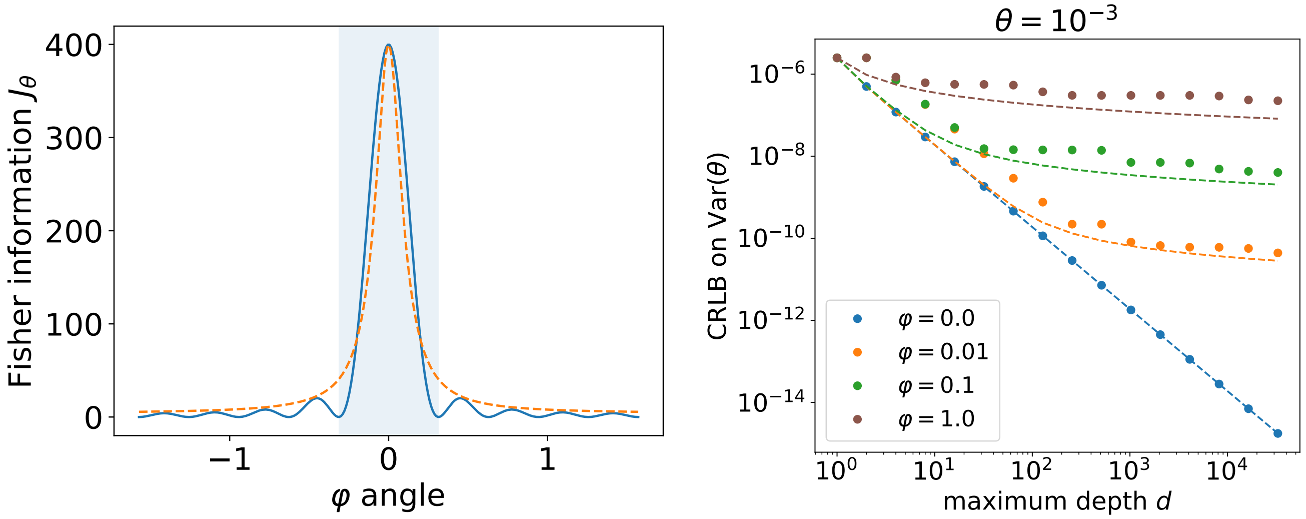

(a)

(b)

Supplementary Figure 16. Approximation analysis of Cramér-Rao lower bound (CRLB). (a) Fisher information of a fixed depth J_θ (blue solid line) and its approximation in Equation (195) (orange dashed line). The shaded area is the principal peak with $|\varphi| \le \pi/d$. We set $d = 10$ and $M = 1$. (b) CRLB (scatters) and its approximation (dashed lines) based on Equation (195). We set $M = 1 \times 10^5$.

We summarize our findings in the following theorem in which for completeness, we explicitly include the phase modulation angle ω as per our original formalism.

Theorem 21. *Let d be the depth parameter of periodic calibration and M be the number of measurement samples in each experiment. When the swap angle θ is small, the optimal estimation variance that periodic calibration can achieve is lower bounded as follows.*

1. *When phase-matching condition is satisfied $\omega = \varphi$, the optimal variance is lower bounded by $\Omega(1/(Md^2))$.*
2. *When phase-matching condition is constantly violated $|\omega - \varphi| \ge$ constant, the optimal variance is lower bounded by $\Omega(1/(M\log(d)))$.*

D. Practical challenges due to the complex optimization landscape

In the previous subsections, we analyze the lower bound on the optimal estimation variance that periodic calibration can achieve in an idealized scenario. However, due to the actual need to minimize a loss function, the estimation performance that periodic calibration can achieve highly depends on the optimization landscape. In this subsection, we analyze the practical challenges that prevent periodic calibration from achieving high estimation accuracy by visualizing the optimization landscape. We consider the following mean squared error (MSE) loss function:

$$\mathcal{L}(\theta, \varphi, d, M) = \sum_{j=0}^{\log_2(d)} \left| \sin^2(\theta) U^2_{2^j-1}(\cos(\theta)\cos(\varphi)) - \hat{P}^{\exp}_{2^j,M} \right|^2 \tag{198}$$

where $\hat{P}^{\exp}_{d,M}$ stands for the experimentally sampled probability using a depth-d circuit with M measurement samples. We numerically study the optimization landscape in Figure 17.

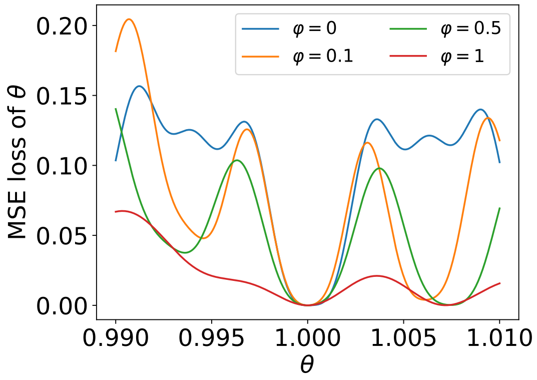

(a)

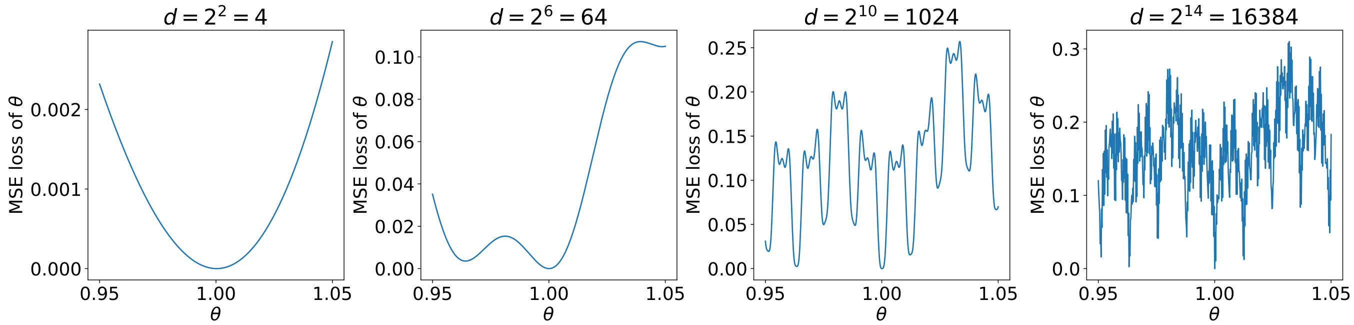

(b)

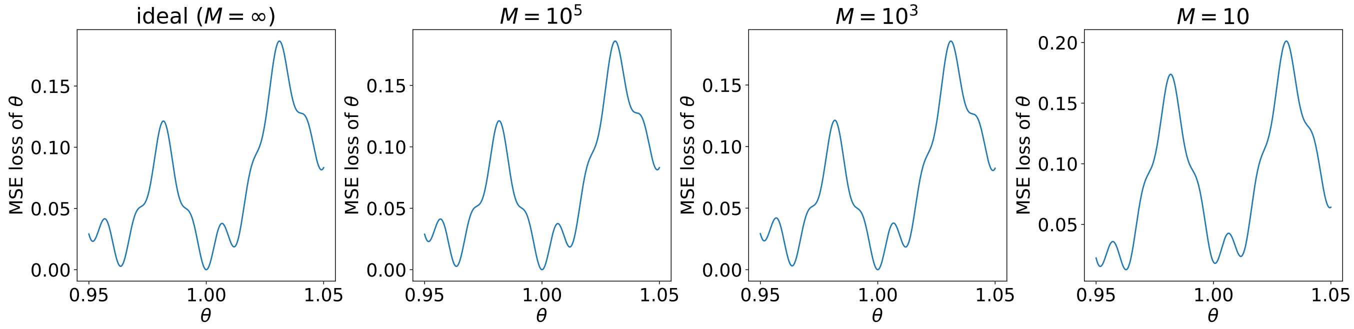

(c)

Supplementary Figure 17. Landscape analysis of periodic calibration. We set the exact value $\theta = 1$ for all figures. (a) Optimization landscape of different φ angles. We set the maximum depth $d = 2^10 = 1024$ and consider an ideal case where infinite many measurement samples are used $M = \infty$. (b) Optimization landscape of different maximum depth parameters d. We consider an ideal case where infinite many measurement samples are used $M = \infty$, and the phase-matching condition is satisfied $\varphi = 0$. (c) Optimization landscape of different numbers of measurement samples M. We set $d = 2^8 = 256$. We consider an ideal case where the phase-matching condition is satisfied $\varphi = 0$.

When the phase-matching condition is violated, we visualize the optimization landscape in Figure 17(a). It can be seen that as φ becomes large, the local minima become increasingly influential. Moreover, some local minima attain an almost vanishing loss value, which makes the optimization challenging.

In Figure 17(b), we visualize the optimization landscape of variable depth parameters. It indicates that the optimization landscape becomes increasingly complex when d is larger. The landscape becomes highly non-convex and has increasingly many local minima. As the local landscape becomes sharper and the local gradient is increasingly large, it is hard for the optimizer to proceed with a reasonable step size. This renders the practical optimization-based solution hard to obtain.

In Figure 17(c), we see that the optimization landscape is not very sensitive to the number of measurement samples. Although the landscape deviates from the true one when $M = 10$ is too small (rightmost panel), the optimization

landscape almost faithfully reproduces the one derived from the ideal case (leftmost panel) when $M \geq 1000$. Hence, the hardness of optimization is less affected by the number of measurement samples but mainly bottlenecked by the exponential disadvantage as circuit depth increases when the phase matching condition is not met.

Supplementary References

[1] R. Acharya, I. Aleiner, R. Allen, T. I. Andersen, M. Ansmann, F. Arute, K. Arya, A. Asfaw, J. Atalaya, R. Babbush, D. Bacon, J. C. Bardin, J. Basso, A. Bengtsson, S. Boixo, G. Bortoli, A. Bourassa, J. Bovaird, L. Brill, M. Broughton, B. B. Buckley, D. A. Buell, T. Burger, B. Burkett, N. Bushnell, Y. Chen, Z. Chen, B. Chiaro, J. Cogan, R. Collins, P. Conner, W. Courtney, A. L. Crook, B. Curtin, D. M. Debroy, A. Del Toro Barba, S. Demura, A. Dunsworth, D. Eppens, C. Erickson, L. Faoro, E. Farhi, R. Fatemi, L. Flores Burgos, E. Forati, A. G. Fowler, B. Foxen, W. Giang, C. Gidney, D. Gilboa, M. Giustina, A. Grajales Dau, J. A. Gross, S. Habegger, M. C. Hamilton, M. P. Harrigan, S. D. Harrington, O. Higgott, J. Hilton, M. Hoffmann, S. Hong, T. Huang, A. Huff, W. J. Huggins, L. B. Ioffe, S. V. Isakov, J. Iveland, E. Jeffrey, Z. Jiang, C. Jones, P. Juhas, D. Kafri, K. Kechedzhi, J. Kelly, T. Khattar, M. Khezri, M. Kieferová, S. Kim, A. Kitaev, P. V. Klimov, A. R. Klots, A. N. Korotkov, F. Kostritsa, J. M. Kreikebaum, D. Landhuis, P. Laptev, K.-M. Lau, L. Laws, J. Lee, K. Lee, B. J. Lester, A. Lill, W. Liu, A. Locharla, E. Lucero, F. D. Malone, J. Marshall, O. Martin, J. R. McClean, T. McCourt, M. McEwen, A. Megrant, B. Meurer Costa, X. Mi, K. C. Miao, M. Mohseni, S. Montazeri, A. Morvan, E. Mount, W. Mruczkiewicz, O. Naaman, M. Neeley, C. Neill, A. Nersisyan, H. Neven, M. Newman, J. H. Ng, A. Nguyen, M. Nguyen, M. Y. Niu, T. E. O'Brien, A. Opremcak, J. Platt, A. Petukhov, R. Potter, L. P. Pryadko, C. Quintana, P. Roushan, N. C. Rubin, N. Saei, D. Sank, K. Sankaragomathi, K. J. Satzinger, H. F. Schurkus, C. Schuster, M. J. Shearn, A. Shorter, V. Shvarts, J. Skruzny, V. Smelyanskiy, W. C. Smith, G. Sterling, D. Strain, M. Szalay, A. Torres, G. Vidal, B. Villalonga, C. Vollgraff Heidweiller, T. White, C. Xing, Z. J. Yao, P. Yeh, J. Yoo, G. Young, A. Zalcman, Y. Zhang, N. Zhu, and G. Q. AI. Suppressing quantum errors by scaling a surface code logical qubit. *Nature*, 614(7949):676–681, 2023. doi:10.1038/s41586-022-05434-1.

[2] F. Arute, K. Arya, R. Babbush, D. Bacon, J. C. Bardin, R. Barends, A. Bengtsson, S. Boixo, M. Broughton, B. B. Buckley, D. A. Buell, B. Burkett, N. Bushnell, Y. Chen, Z. Chen, Y.-A. Chen, B. Chiaro, R. Collins, S. J. Cotton, W. Courtney, S. Demura, A. Derk, A. Dunsworth, D. Eppens, T. Eckl, C. Erickson, E. Farhi, A. Fowler, B. Foxen, C. Gidney, M. Giustina, R. Graff, J. A. Gross, S. Habegger, M. P. Harrigan, A. Ho, S. Hong, T. Huang, W. Huggins, L. B. Ioffe, S. V. Isakov, E. Jeffrey, Z. Jiang, C. Jones, D. Kafri, K. Kechedzhi, J. Kelly, S. Kim, P. V. Klimov, A. N. Korotkov, F. Kostritsa, D. Landhuis, P. Laptev, M. Lindmark, E. Lucero, M. Marthaler, O. Martin, J. M. Martinis, A. Marusczyk, S. McArdle, J. R. McClean, T. McCourt, M. McEwen, A. Megrant, C. Mejuto-Zaera, X. Mi, M. Mohseni, W. Mruczkiewicz, J. Mutus, O. Naaman, M. Neeley, C. Neill, H. Neven, M. Newman, M. Y. Niu, T. E. O'Brien, E. Ostby, B. Pató, A. Petukhov, H. Putterman, C. Quintana, J.-M. Reiner, P. Roushan, N. C. Rubin, D. Sank, K. J. Satzinger, V. Smelyanskiy, D. Strain, K. J. Sung, P. Schmitteckert, M. Szalay, N. M. Tubman, A. Vainsencher, T. White, N. Vogt, Z. J. Yao, P. Yeh, A. Zalcman, and S. Zanker. Observation of separated dynamics of charge and spin in the Fermi-Hubbard model. *arXiv:2010.07965 [quant-ph]*, Oct. 2020. arXiv: 2010.07965. URL: http://arxiv.org/abs/2010.07965.

[3] F. Arute, K. Arya, R. Babbush, D. Bacon, J. C. Bardin, R. Barends, R. Biswas, S. Boixo, F. G. Brandao, D. A. Buell, et al. Quantum supremacy using a programmable superconducting processor. *Nature*, 574(7779):505–510, 2019.

[4] S. Boixo, S. V. Isakov, V. N. Smelyanskiy, R. Babbush, N. Ding, Z. Jiang, M. J. Bremner, J. M. Martinis, and H. Neven. Characterizing quantum supremacy in near-term devices. *Nature Physics*, 14(6):595–600, 2018.

[5] S. L. Braunstein and C. M. Caves. Statistical distance and the geometry of quantum states. *Physical Review Letters*, 72(22):3439, 1994.

[6] Y. Dong, X. Meng, K. B. Whaley, and L. Lin. Efficient phase-factor evaluation in quantum signal processing. *Physical Review A*, 103(4):042419, 2021.

[7] B. Foxen, C. Neill, A. Dunsworth, P. Roushan, B. Chiaro, A. Megrant, J. Kelly, Z. Chen, K. Satzinger, R. Barends, F. Arute, K. Arya, R. Babbush, D. Bacon, J. C. Bardin, S. Boixo, D. Buell, B. Burkett, Y. Chen, R. Collins, E. Farhi, A. Fowler, C. Gidney, M. Giustina, R. Graff, M. Harrigan, T. Huang, S. V. Isakov, E. Jeffrey, Z. Jiang, D. Kafri, K. Kechedzhi, P. Klimov, A. Korotkov, F. Kostritsa, D. Landhuis, E. Lucero, J. McClean, M. McEwen, X. Mi, M. Mohseni, J. Y. Mutus, O. Naaman, M. Neeley, M. Niu, A. Petukhov, C. Quintana, N. Rubin, D. Sank, V. Smelyanskiy, A. Vainsencher, T. C. White, Z. Yao, P. Yeh, A. Zalcman, H. Neven, and J. M. Martinis. Demonstrating a continuous set of two-qubit gates for near-term quantum algorithms. *Phys. Rev. Lett.*, 125:120504, Sep 2020. URL: https://link.aps.org/doi/10.1103/PhysRevLett.125.120504, doi:10.1103/PhysRevLett.125.120504.

[8] A. Gilyén, Y. Su, G. H. Low, and N. Wiebe. Quantum singular value transformation and beyond: exponential improvements for quantum matrix arithmetics. In *Proceedings of the 51st Annual ACM SIGACT Symposium on Theory of Computing*, pages 193–204. ACM, 2019.

[9] V. Giovannetti, S. Lloyd, and L. Maccone. Quantum metrology. *Phys. Rev. Lett.*, 96:010401, Jan 2006. URL: https://link.aps.org/doi/10.1103/PhysRevLett.96.010401, doi:10.1103/PhysRevLett.96.010401.

[10] S. Kay. A fast and accurate single frequency estimator. *IEEE Transactions on Acoustics, Speech, and Signal Processing*, 37(12):1987–1990, 1989.

[11] R. W. Keener. *Theoretical statistics: Topics for a core course.* Springer, 2010.

[12] S. Kimmel, G. H. Low, and T. J. Yoder. Robust calibration of a universal single-qubit gate set via robust phase estimation. *Physical Review A*, 92(6):062315, 2015.

[13] I. Kull, P. A. Guérin, and F. Verstraete. Uncertainty and trade-offs in quantum multiparameter estimation. *Journal of Physics A: Mathematical and Theoretical*, 53(24):244001, 2020.

[14] G. H. Low and I. L. Chuang. Optimal hamiltonian simulation by quantum signal processing. *Physical review letters*, 118(1):010501, 2017.

[15] A. A. Markov. On a question by di mendeleev. *Zapiski Imperatorskoi Akademii Nauk*, 62(1-24):12, 1890.

[16] J. M. Martyn, Z. M. Rossi, A. K. Tan, and I. L. Chuang. Grand unification of quantum algorithms. *PRX Quantum*, 2(4):040203, 2021.

[17] C. Neill, T. McCourt, X. Mi, Z. Jiang, M. Y. Niu, W. Mruczkiewicz, I. Aleiner, F. Arute, K. Arya, J. Atalaya, R. Babbush, J. C. Bardin, R. Barends, A. Bengtsson, A. Bourassa, M. Broughton, B. B. Buckley, D. A. Buell, B. Burkett, N. Bushnell, J. Campero, Z. Chen, B. Chiaro, R. Collins, W. Courtney, S. Demura, A. R. Derk, A. Dunsworth, D. Eppens, C. Erickson, E. Farhi, A. G. Fowler, B. Foxen, C. Gidney, M. Giustina, J. A. Gross, M. P. Harrigan, S. D. Harrington, J. Hilton, A. Ho, S. Hong, T. Huang, W. J. Huggins, S. V. Isakov, M. Jacob-Mitos, E. Jeffrey, C. Jones, D. Kafri, K. Kechedzhi, J. Kelly, S. Kim, P. V. Klimov, A. N. Korotkov, F. Kostritsa, D. Landhuis, P. Laptev, E. Lucero, O. Martin, J. R. McClean, M. McEwen, A. Megrant, K. C. Miao, M. Mohseni, J. Mutus, O. Naaman, M. Neeley, M. Newman, T. E. O'Brien, A. Opremcak, E. Ostby, B. Pató, A. Petukhov, C. Quintana, N. Redd, N. C. Rubin, D. Sank, K. J. Satzinger, V. Shvarts, D. Strain, M. Szalay, M. D. Trevithick, B. Villalonga, T. C. White, Z. Yao, P. Yeh, A. Zalcman, H. Neven, S. Boixo, L. B. Ioffe, P. Roushan, Y. Chen, and V. Smelyanskiy. Accurately computing the electronic properties of a quantum ring. *Nature*, 594(7864):508–512, June 2021. URL: https://www.nature.com/articles/s41586-021-03576-2, doi:10.1038/s41586-021-03576-2.

[18] M. Y. Niu, S. Boixo, V. N. Smelyanskiy, and H. Neven. Universal quantum control through deep reinforcement learning. *npj Quantum Information*, 5(1):1–8, 2019.

[19] Z. Shen and R. Liu. Efficient and accurate frequency estimator under low SNR by phase unwrapping. *Mathematical Problems in Engineering*, 2019, 2019.

[20] S. Tretter. Estimating the frequency of a noisy sinusoid by linear regression (corresp.). *IEEE Transactions on Information theory*, 31(6):832–835, 1985.

[21] J. Wang, Y. Dong, and L. Lin. On the energy landscape of symmetric quantum signal processing. *Quantum*, 6:850, 2022.
